# Supplementary material for: Quantitative RNA spatial profiling using single-molecule RNA FISH on plant tissue cryosections
Source: Plant Commun. 2026 Jun 8;7(8):101943. doi: 10.1016/j.xplc.2026.101943 (PMC13477017; doi:10.1016/j.xplc.2026.101943)
Supplement: Supplementary file 1 [file mmc1.pdf]

Plant Communications, Volume 7

Supplemental information

Quantitative RNA spatial profiling using single-molecule RNA FISH on plant tissue cryosections

Xue Zhang, Alejandro Fonseca, Konstantin Kutashev, Martina Leso, Adrien Sicard, Susan Duncan, and Stefanie Rosa

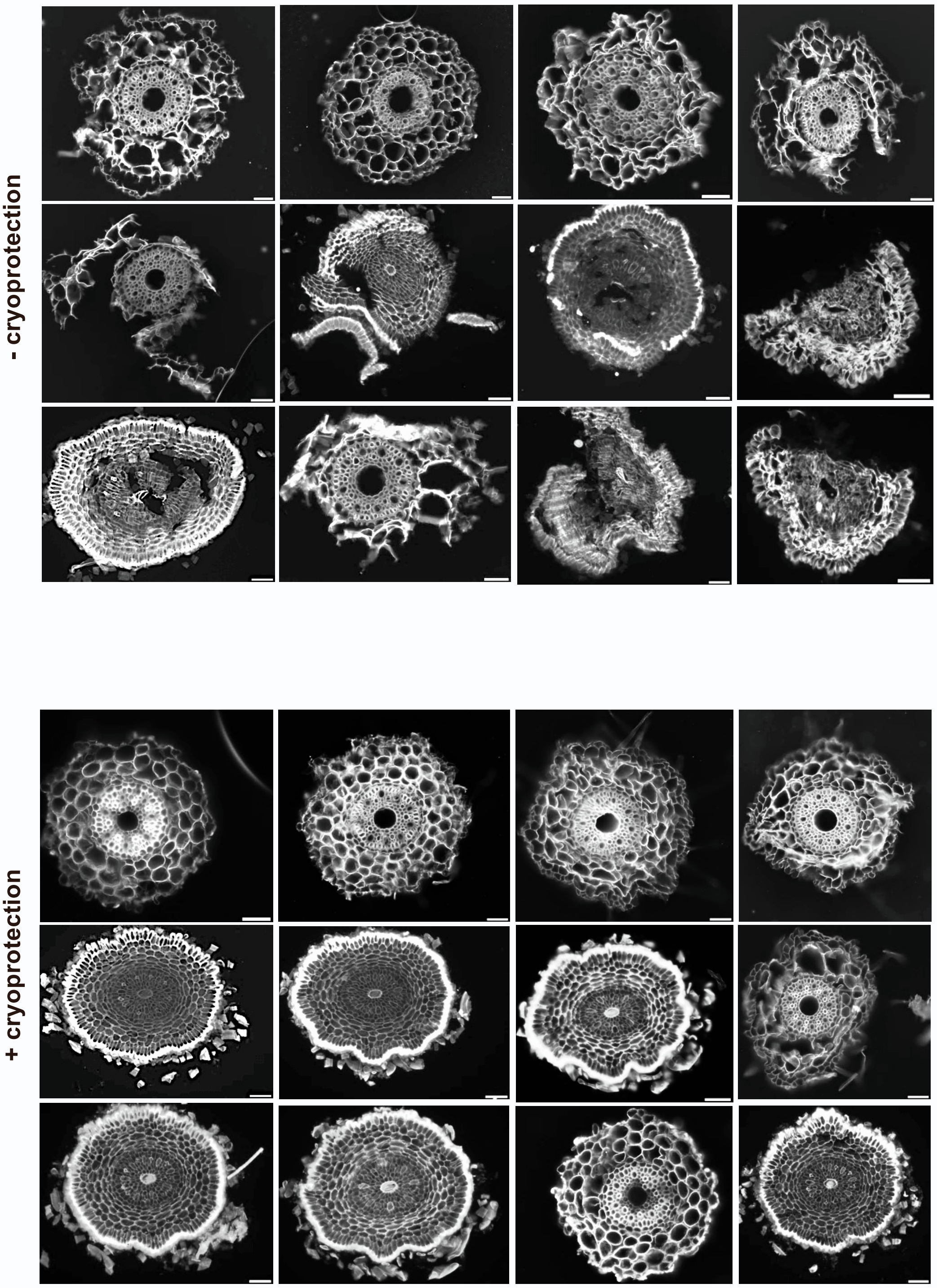

Figure S1. Integrity of barley root sections with and without cryoprotection. Representative widefield microscopy images of barley root cryosections with and without employment of a cryoprotection step. Root sections were stained with SR2200 dye. Scale bars, 10µm.

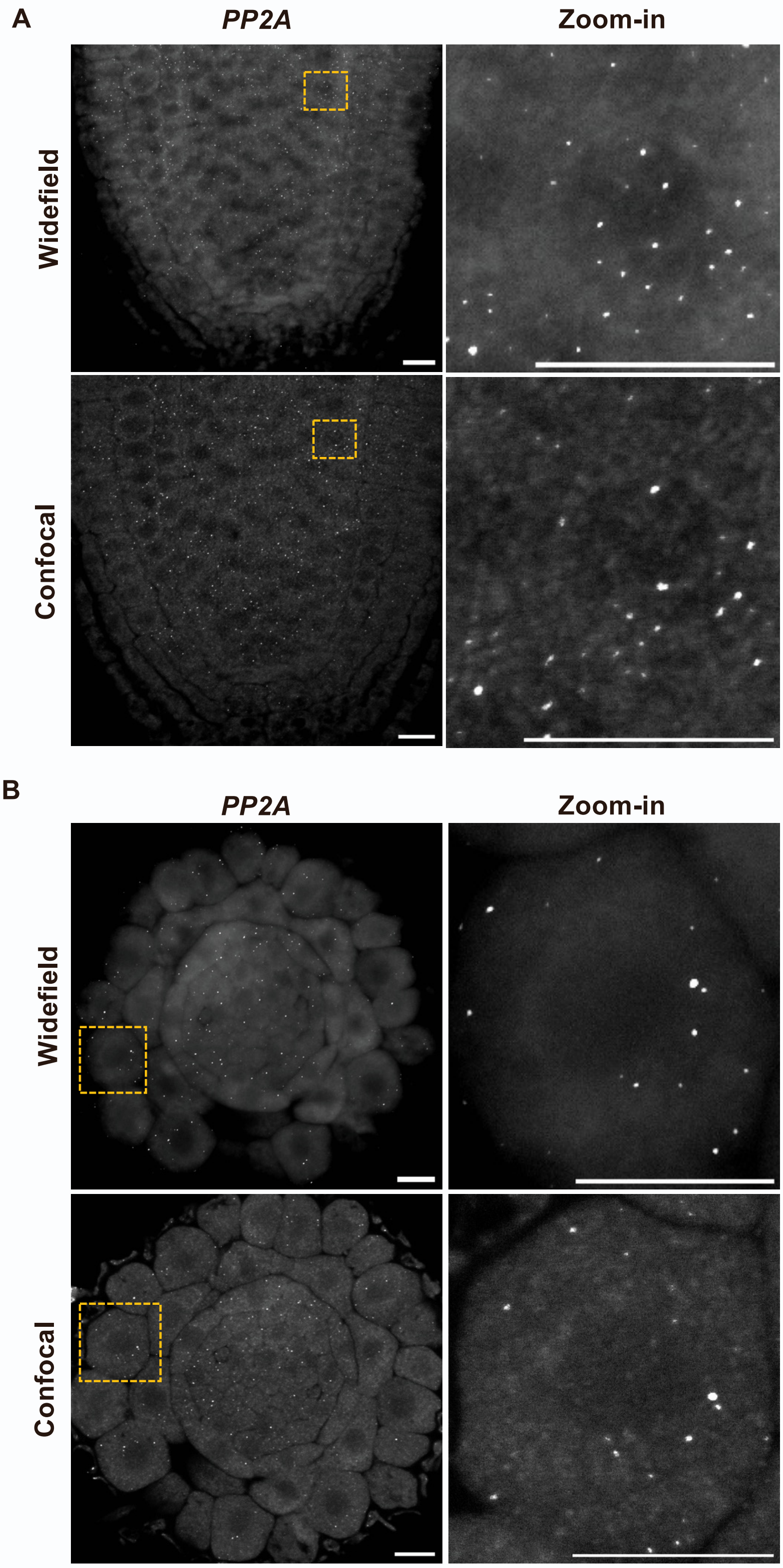

Figure S2. smFISH on *Arabidopsis* root longitudinal and cross-cryosections.
Detection of *PP2A* mRNA using smFISH on *Arabidopsis* root longitudinal (A) and cross-sections (B) using widefield and confocal (single- plane) microscopy. Zoom-in images from regions highlighted in yellow on the left panel. Discrete white dots above the diffuse background correspond to individual RNA molecules. Scale bars, 10µm.

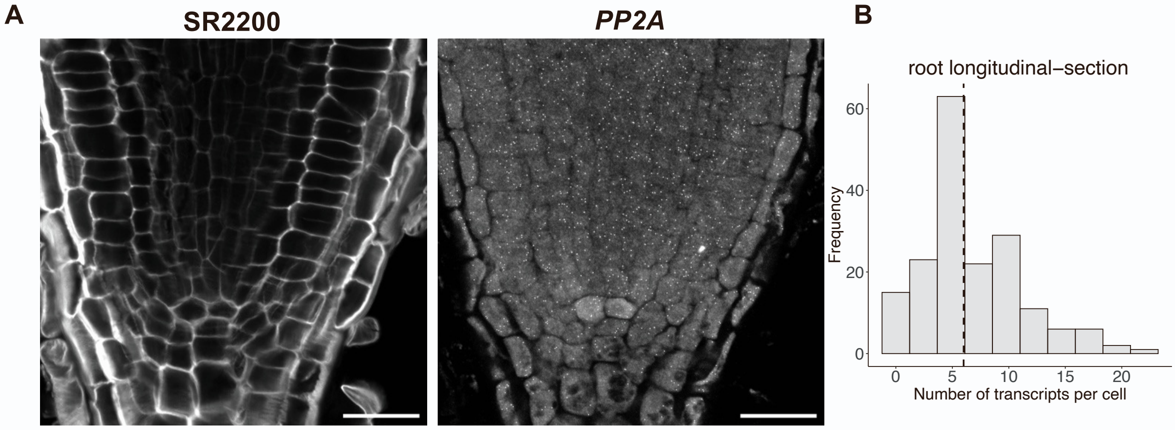

Figure S3. *PP2A* mRNA detection in *Arabidopsis* longitudinal root cryosections. (A) Representative single-plane confocal images of *PP2A* mRNA detected by cryo-smFISH on *Arabidopsis* root longitudinal-section. Left panel: an overview of the tissue using celll wall SR2200 dye; Right panel: *PP2A* cryo-smFISH signals (white dots) corresponding to individual mRNA molecules. Scale bars, 10µm. (C) Histogram showing the distribution of the number of transcripts per cell within the imaged volume detected on root longitudinal-section. The dashed lines mark the median value.

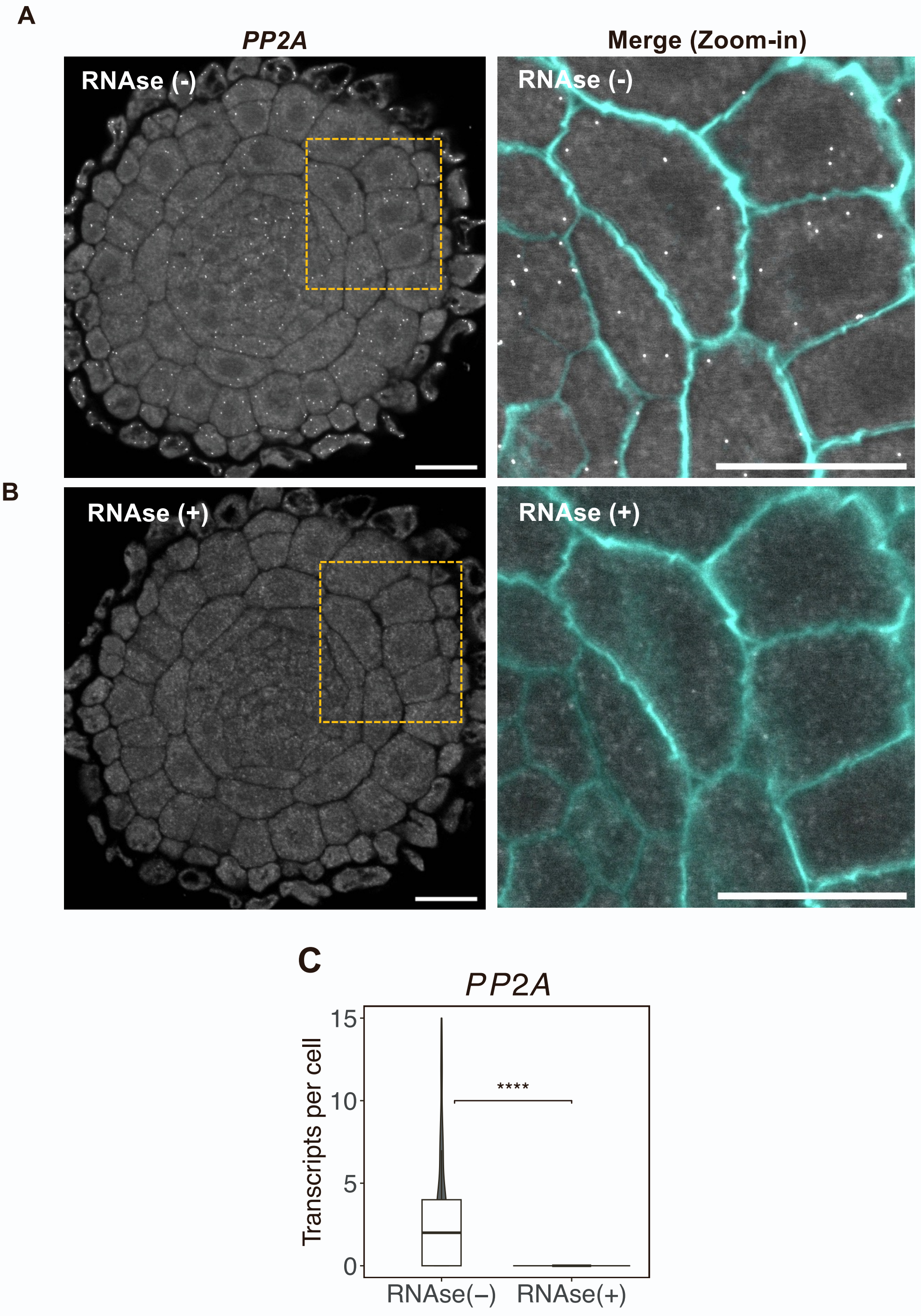

Figure S4. *PP2A* mRNA detection and quantification using cryo-smFISH on *Arabidopsis* root cross-cryosections. (A-B) Representative single-plane confocal images of cryo-smFISH using probes targeting *PP2A* mRNA in *Arabidopsis* root cross-cryosections before (A) and after RNAse treatment (B)**.** Zoom-in images (right panels) of regions highlighted in yellow on the left panel. Discrete white dots correspond to individual RNA molecules. Cell walls stained with SR2200 dye (cyan). Scale bars, 15µm. (C) Violin plot showing the number of transcripts per cell within the imaged volume detected before RNAse (-) and after RNAse (+) treatment. Boxes inside indicate the interquartile range (IQR 25-75%), depicting the median values as a horizontal line. Whiskers represent the ±1.58xIQR value. A two-sided t-test was performed to compare both conditions. p-value less than 0.0001 was denoted by ****.

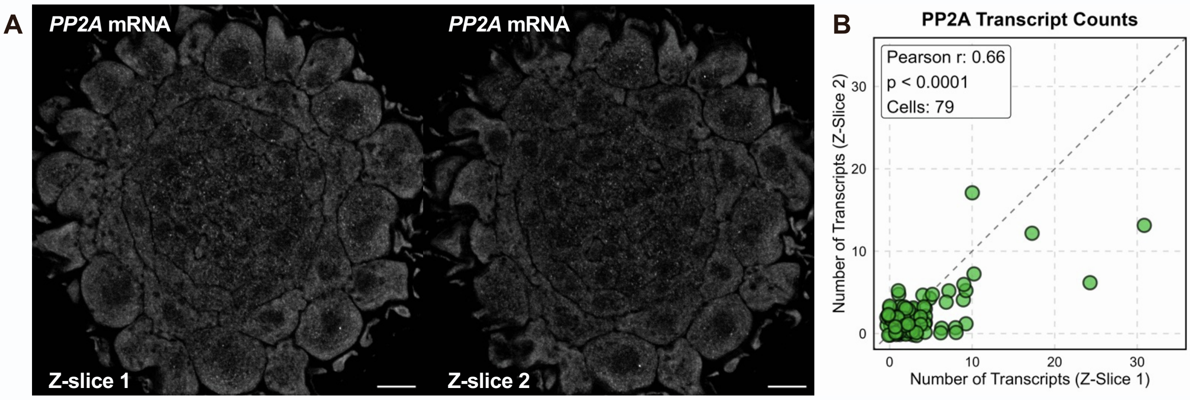

Figure S5. Correlation of *PP2A* transcripts counts across different optical sections. (A) Single-plane confocal images from cryo-smFISH and **(B)** Scatter plot comparing the detected number of *PP2A* transcripts within identical cells across two distinct Z-slice (separated by a 1 µm distance) of a single Arabidopsis root cross-section (each data point represents one cell). The dashed gray line represents a theoretical 1:1 perfect correlation. The Pearson correlation coefficient (r), its associated p-value, and the total number of analyzed cells are indicated in the top left corner of the plot.

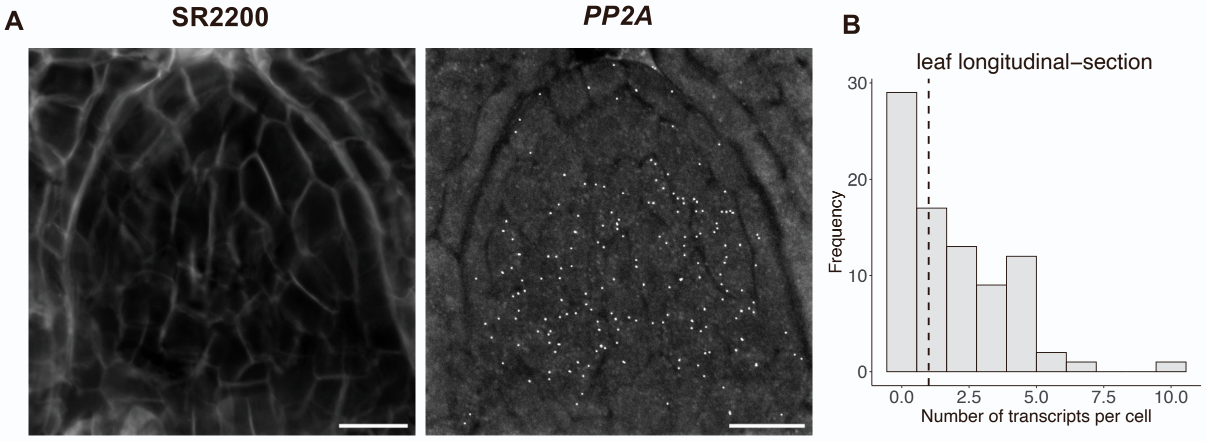

Figure S6. *PP2A* mRNA detection in *Arabidopsis* young leaf cryosections. (A) Representative single-plane confocal images of *PP2A* mRNA detected by cryo-smFISH on *Arabidopsis* young leaf section. Left panel: an overview of the tissue using celll wall SR2200 dye; Right panel: *PP2A* cryo-smFISH signals (white dots) corresponding to individual mRNA molecules. Scale bars, 10μm. (C) Histogram showing the distribution of the number of transcripts per cell within the imaged volume detected on the section. The dashed lines mark the median value.

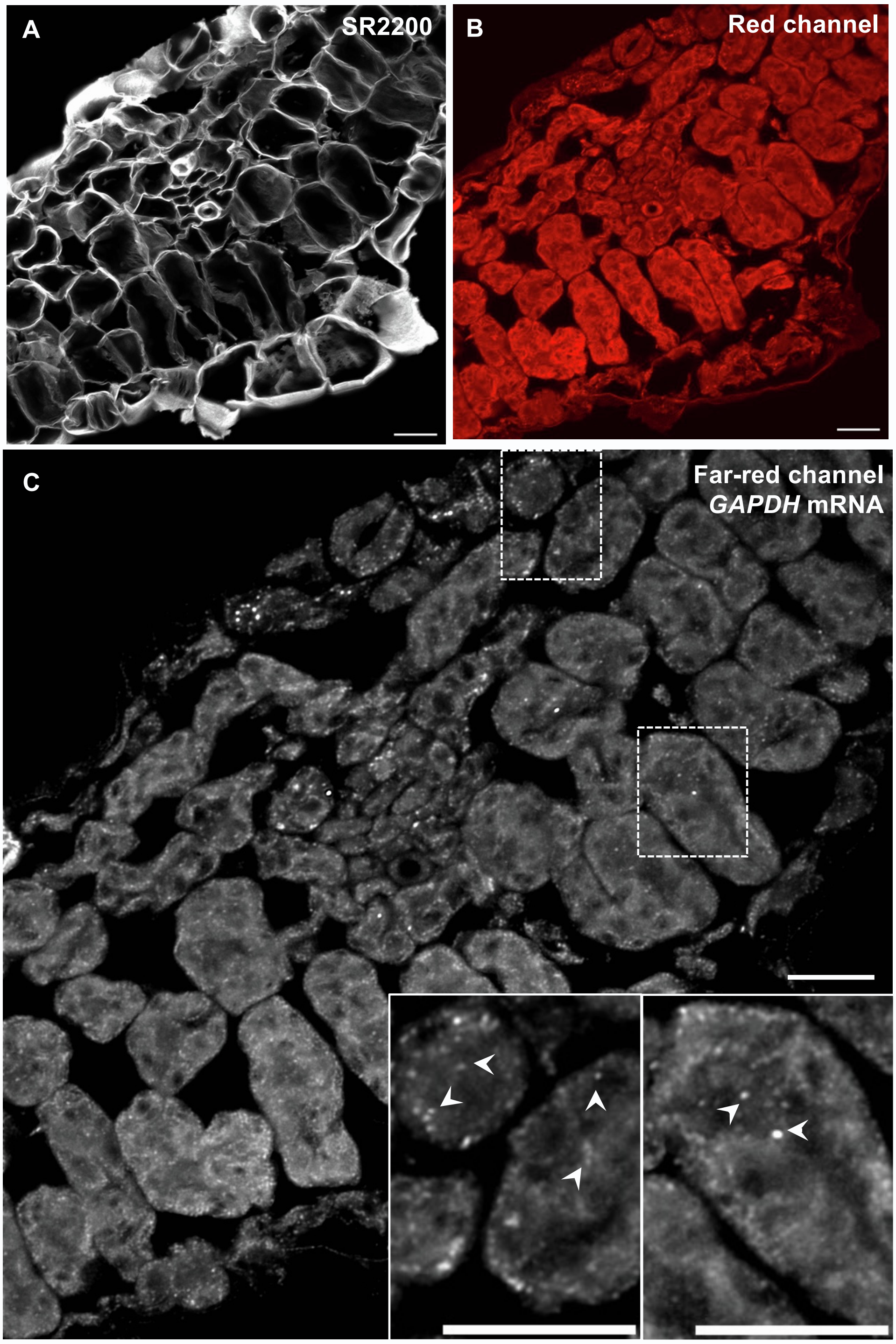

Figure S7. *GAPDH* mRNA detection in *Arabidopsis* cross sections from adult leaves. (A) Overview of the tissue with cell wall staining using SR2200 dye. (B) Autofluorescence in the red channel. (C) Detection of *GAPDH* using smFISH probes labelled with far-red dye (Quasar670). Images represent a maximum intensity projection of five Z-stacks with a Z-step of 0.5 µm. Raw images were deconvolved using the NIS-Elements 3D deconvolution algorithm prior to generating the projection. Arrows indicate mRNA detection. Inset: magnified view of the region highlighted. Scale bars, 10 µm.

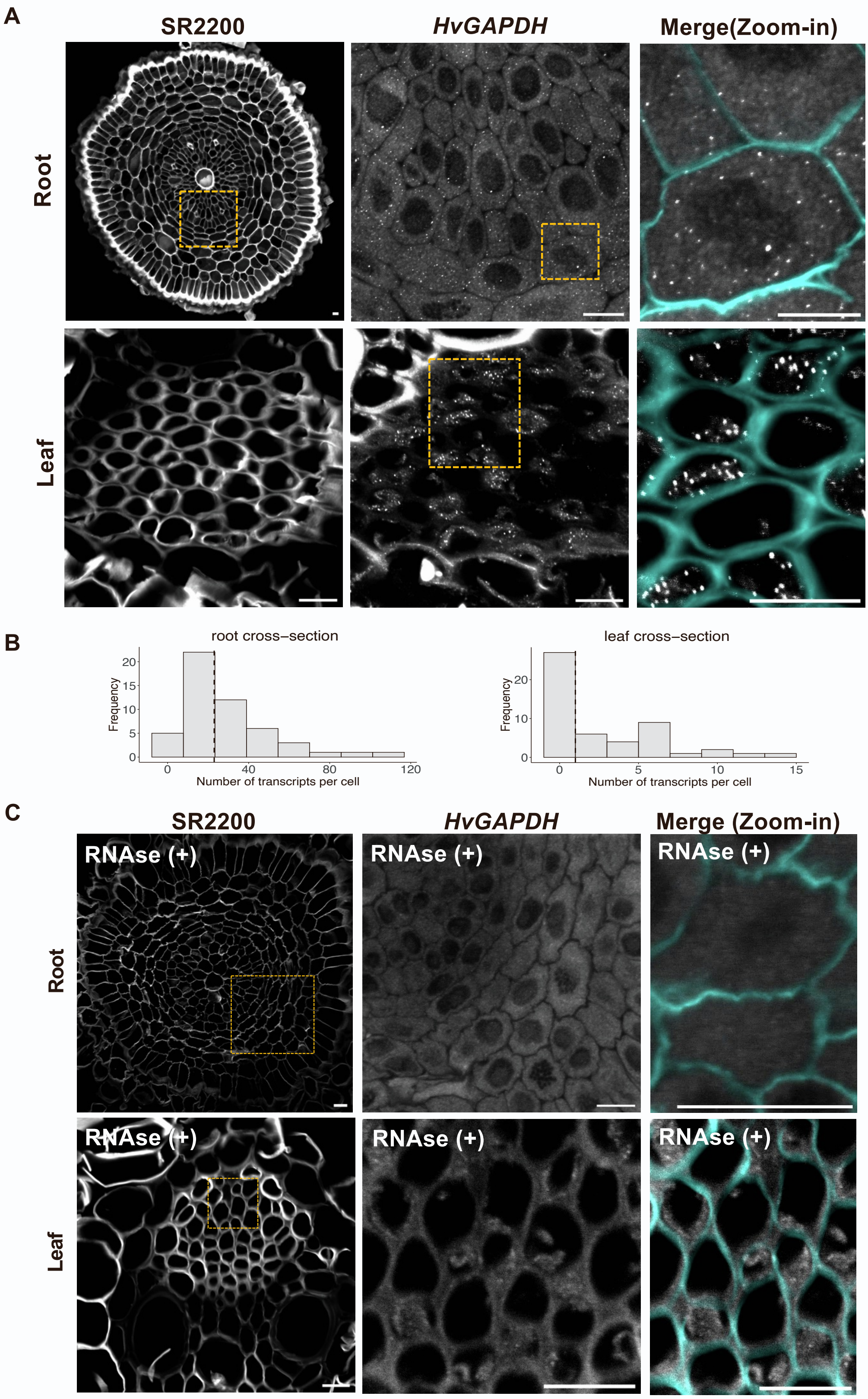

Figure S8. Detecting *HvGAPDH* mRNA in barley roots and leaves using cryo-smFISH.
(A, C) Representative single-plane confocal images of cryo-smFISH using probes targeting *HvGAPDH* mRNA in barley root and leaf cross-sections with (C) and without (A) RNAse treatment**.** Left panels: cell wall staining with SR2200 dye; Middle panels: cryo-smFISH channel; Right panels: zoomed-in images of regions highlighted in yellow in the left panels showing cryo-smFISH channel (white) and cell wall staining (cyan). Scale bars, 10µm. (B) Histograms showing the distribution of the number of transcripts per cell within the imaged volume detected in the root (left) and leaf cross-sections (right). Dashed lines indicate the median value. Experiments were repeated independently 2 times.

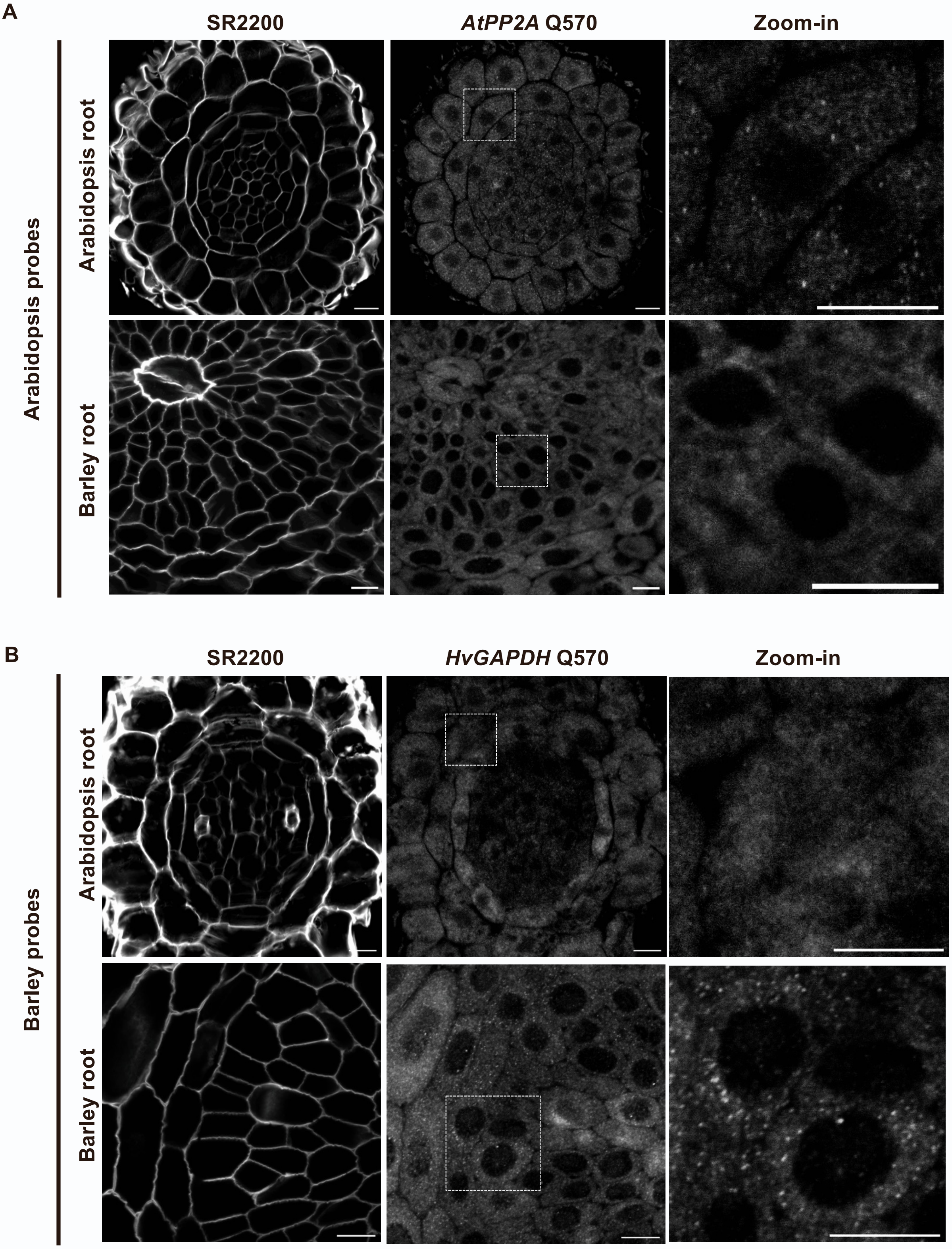

Figure S9. Cross-species validation of cryo-smFISH probe specificity.

(A) Representative single-plane confocal images of cryo-smFISH using probes designed to detect *PP2A* mRNA from *Arabidopsis.* Root cross-cryosections from Arabidopsis and barley were tested, showing signals exclusively in Arabidopsis samples. (B) Representative images of cryo-smFISH using probes designed to detect *GAPDH* mRNA from barley. Root cross-cryosections from *Arabidopsis* and barley were tested, showing signals exclusively in barley samples. Left panels: cell wall staining with SR2200 dye; Middle panels: smFISH channel; Right panels: zoomed-in images of smFISH channel. Scale bars, 10µm.

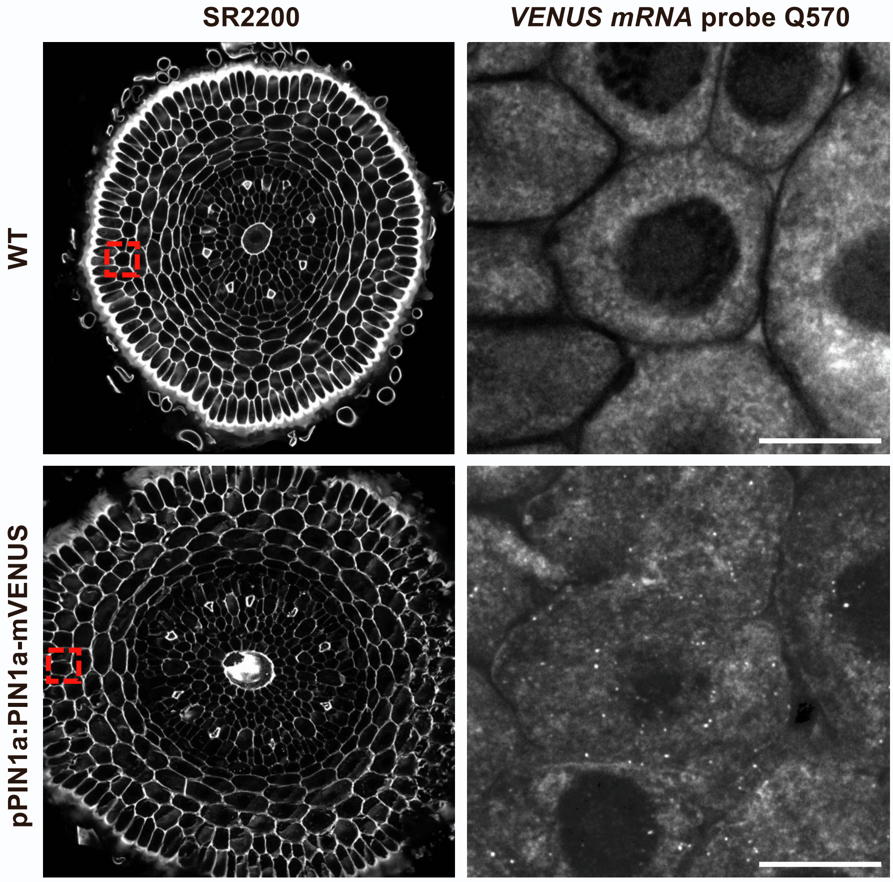

Figure S10. smFISH probe specificity control using *VENUS* mRNA probes
Representative single-plane confocal images of cryo-smFISH using Quasar570-probes designed to detect *VENUS* mRNA. Root cross-cryosections from barley were tested, showing signals exclusively in the transgenic line pPIN1a:PIN1a-mVENUS. Left panels: cell wall staining with SR2200 dye; Right panels: zoomed-in images of smFISH channel. Scale bars, 10µm.

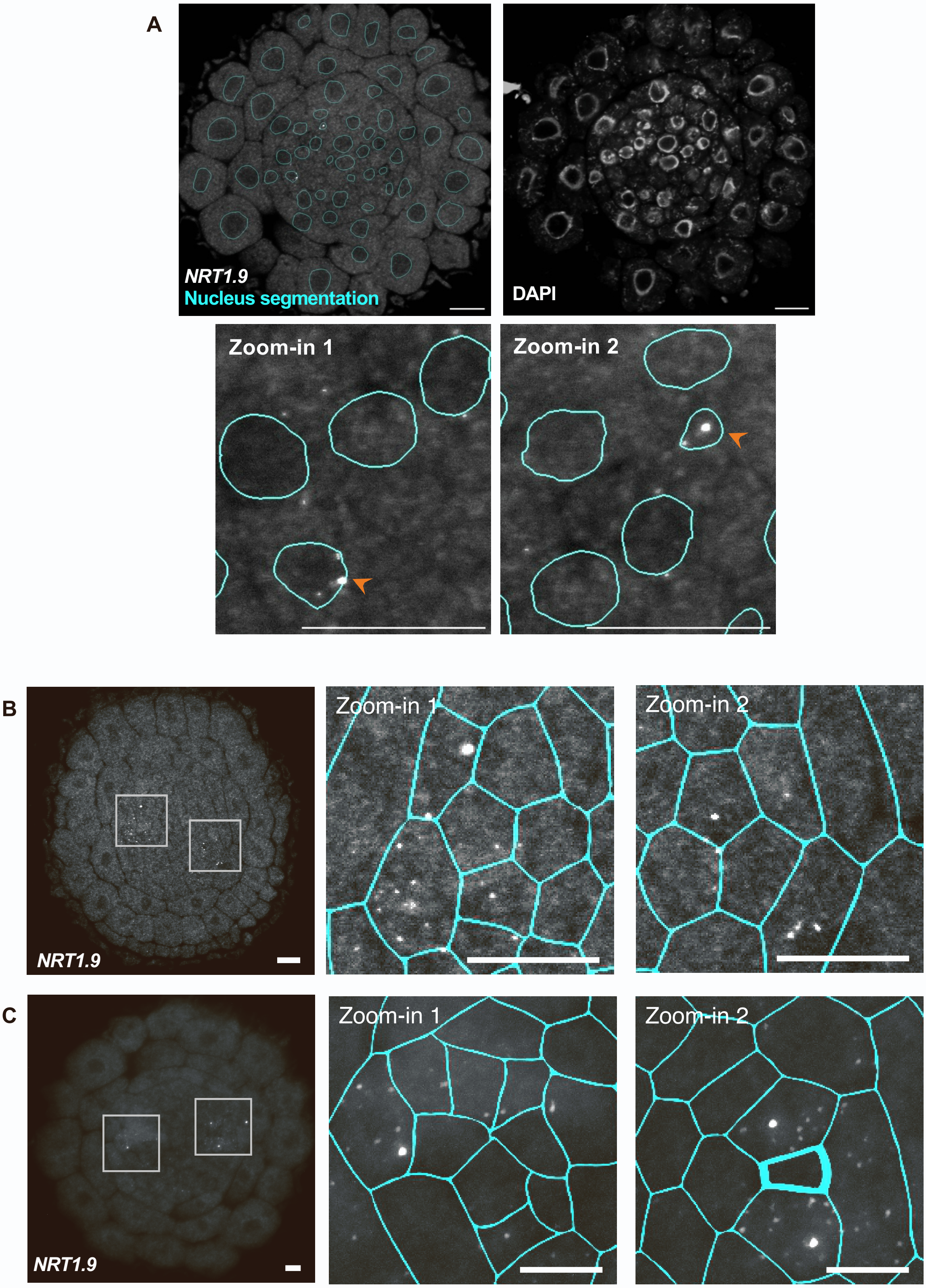

Figure S11. *NRT1.9* expression pattern visualized by cryo-smFISH.
(A-C) Representative images of cryo-smFISH using probes targeting *NRT1.9* mRNA in Arabidopsis root cross-cryosections. Images in (A) and (B) are single-plane confocal images, whereas the image in (C) is a maximum intensity projection from 17 Z-slices acquired with widefield microscopy. (A) Lower row: zoomed-in images showing *NRT1.9* transcripts (white dots). The orange arrows indicate *NRT1.9* RNA foci. Nucleus outline (cyan) obtained from segmentation using DAPI. (B-C) Middle and right panels: zoomed-in images showing *NRT1.9* transcripts (white dots). Cell outlines (cyan) obtained from segmentation using SR2200 dye. Scale bars, 10 µm.

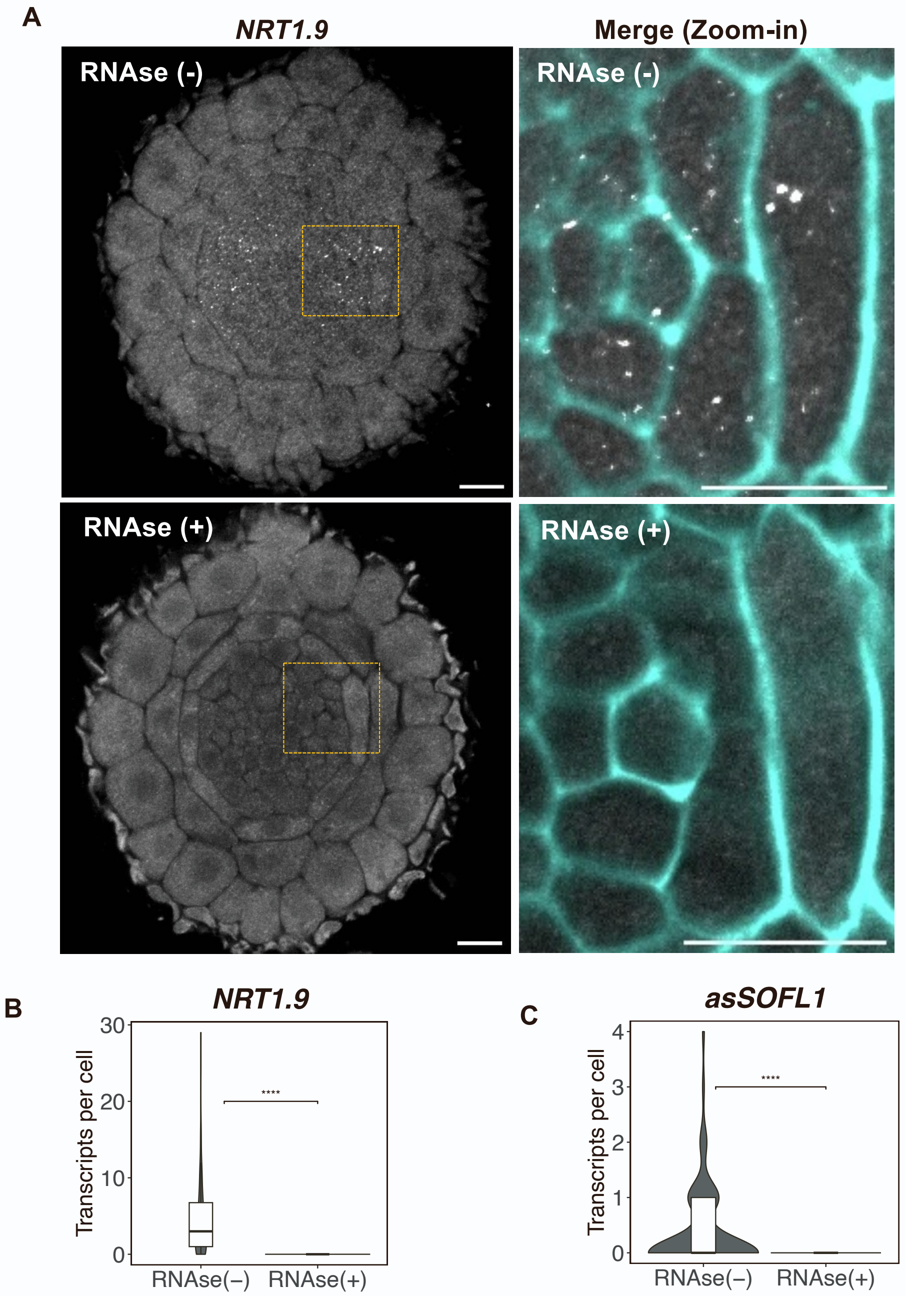

Figure S12. RNAse A controls for cryo-smFISH experiments on *NRT1.9* and *asSOFL1*.
(A) Representative single-plane confocal images of cryo-smFISH using probes targeting *NRT1.9* mRNA in *Arabidopsis* root cross-cryosections before (-) and after (+) RNAse treatment**.** Zoom-in images (right panels) of regions highlighted in yellow on the left panel. Discrete white dots correspond to individual RNA molecules. Cell walls are stained with SR2200 dye (cyan). Scale bars, 10µm. (B, C) Violin plots showing the distributions and quantification of the number of transcripts per cell within the imaged volume before RNAse (-) and after RNAse (+) treatment for *NRT1.9* and *asSOFL1*. The dashed lines in histograms indicate the median value. Violin plots: boxes inside indicate the interquartile range (IQR 25-75%), depicting the median values as a horizontal line. Whiskers represent the ±1.58xIQR value. A two-sided t-test was performed to compare both conditions. p-value less than 0.0001 was denoted by ****.

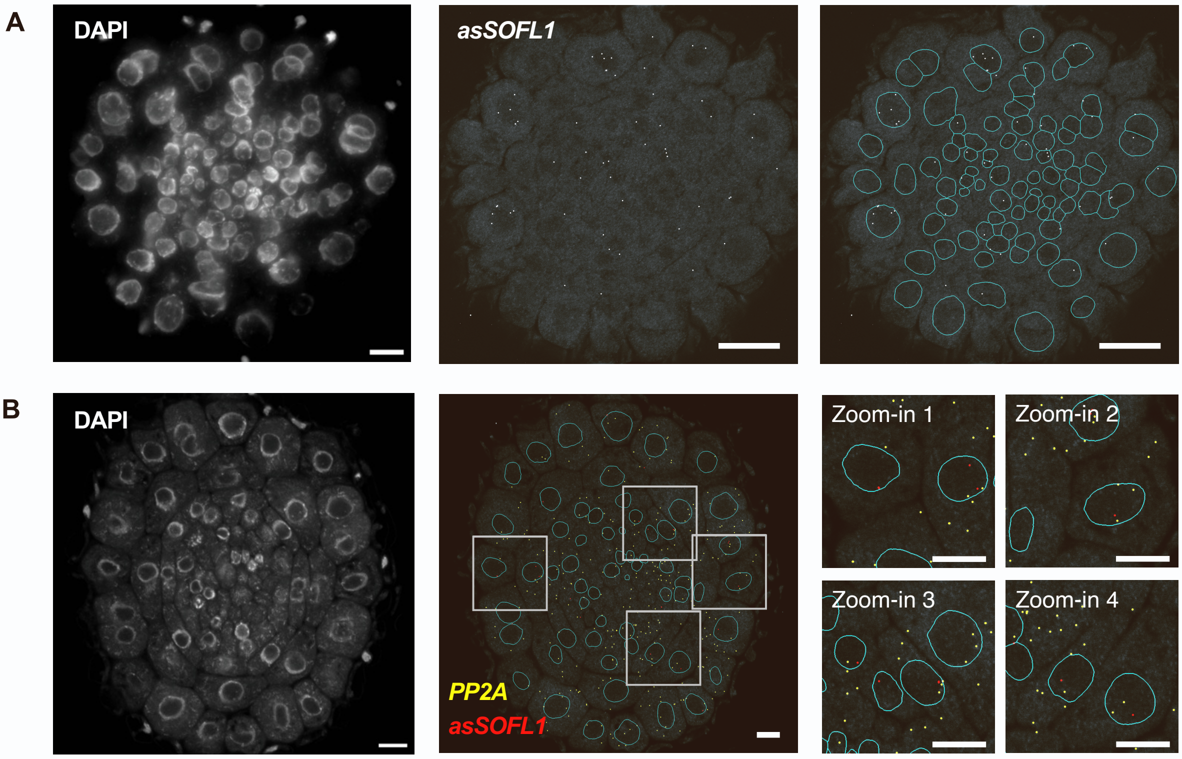

Figure S13. *asSOFL1* expression pattern visualized by cryo-smFISH.
(A) Representative Z project images of cryo-smFISH using probes targeting *asSOFL1* RNA in Arabidopsis root cross-cryosections. Right panel: image showing *asSOFL1* (white dots) transcripts within nucleus. Nucleus outlines obtained from segmentation using DAPI (blue). (B) Representative confocal image of cryo-smFISH using probes targeting *asSOFL1* (red dots) and *PP2A* (yellow dots) RNA in Arabidopsis root cross-cryosections. Right panel: zoom-in images. Scale bars, 10µm.

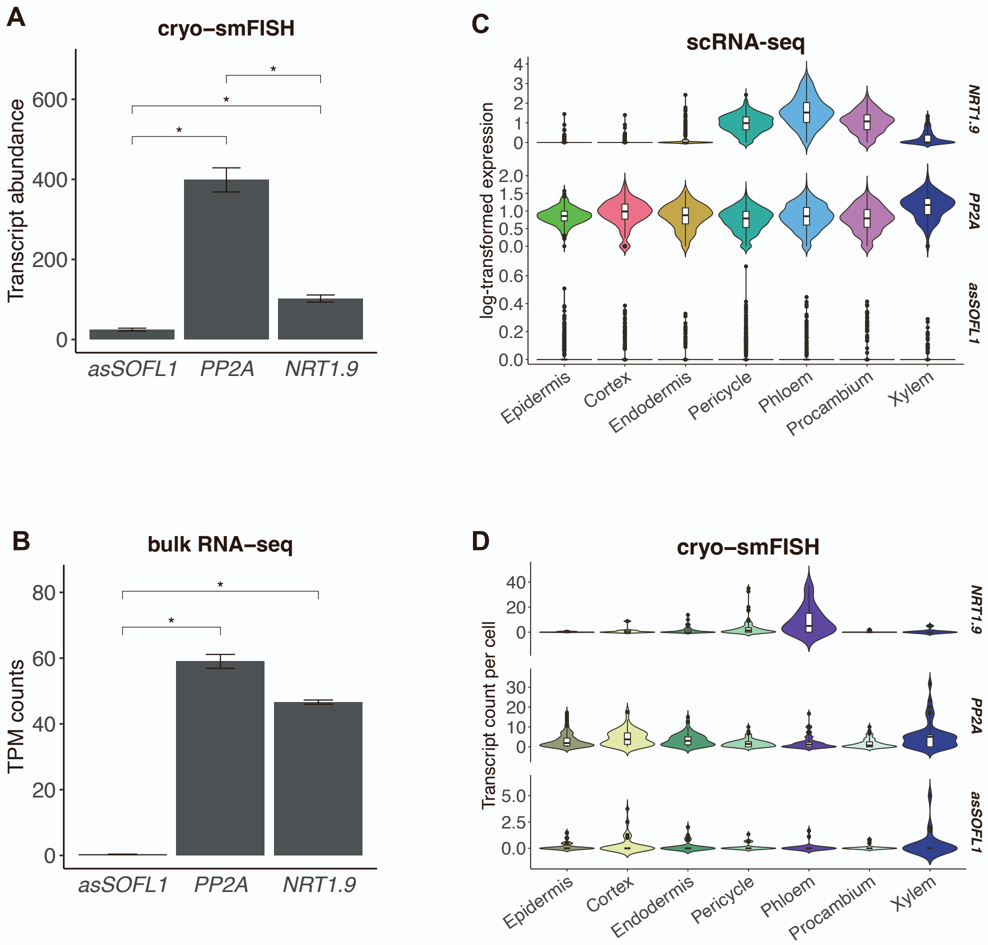

Figure S14: Comparison of *NRT1.9*, *PP2A* and *asSOFL1* expression between scRNA-seq, smFISH and bulk RNA-seq. (A, B) Bar plots showing the expression of *NRT1.9*, *PP2A*, and *asSOFL1* in the *Arabidopsis* root tip using cryo-smFISH (A) and bulk RNA-seq data (B). Error bars represent the mean ± SEM. Quantitative data for smFISH and bulk RNA-seq results originated from three replicates and two replicates, respectively. A two-sided t-test was performed to compare both conditions. A p-value greater than 0.05 indicates no statistical significance (not shown), while p-values less than 0.05, 0.001, and 0.0001 were denoted by*, **, and ***, respectively.(C, D) Violin plots showing the expression of *NRT1.9*, *PP2A,* and *asSOFL1* in individual cells detected by scRNA-seq (C) and cryo-smFISH (D). The number of transcripts is normalized by the number of cells within the cell type. The plotting area is scaled by width. Violin plots: boxes inside correspond to the interquartile range (IQR 25-75%), and horizontal lines indicate the median values. Whiskers show the ±1.58xIQR value. Statistical analyses were performed with one-way ANOVA followed by Tukey's honestly significant difference (HSD) tests.

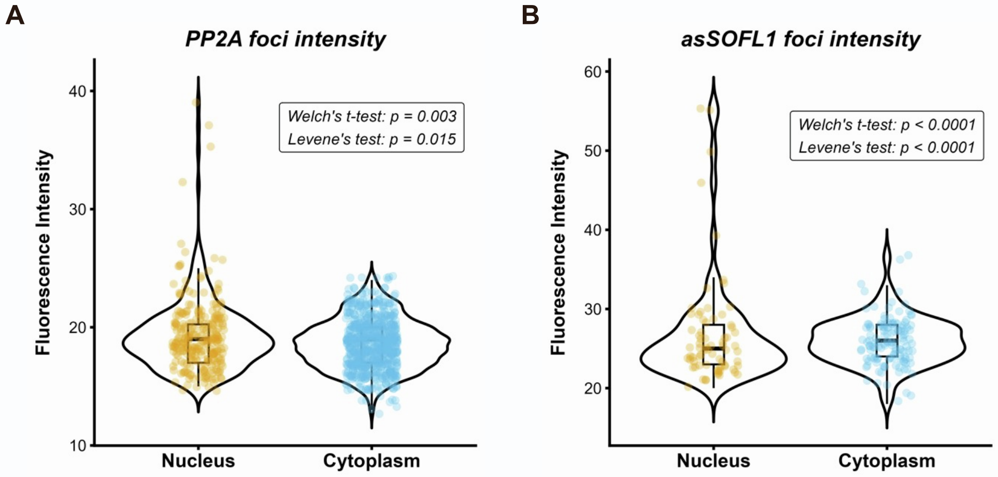

Figure S15. Subcellular smFISH signal intensity of *PP2A* and *asSOFL1* detected foci. Violin plots showing the raw fluorescence intensity of individual smFISH foci detected in the nucleus (orange) and cytoplasm (blue) for *asSOFL1* **(A)** and *PP2A* **(B)**. Data were pooled from 4 independent *Arabidopsis* root cross-sections, comprising a total of 953 detected transcripts for *PP2A* and 202 detected transcripts for *asSOFL1*. Individual data points are overlaid as jitter plots, where each dot indicates an individual detected spot. Boxes inside violin plots show the interquartile range (IQR 25-75%), indicating the median values as a horizontal line. Statistical analyses comparing the mean intensities between nuclear and cytoplasmic compartments were performed using Welch's t-test, and differences in variance were assessed using Levene's test. The respective p-values are indicated in the top right corner of each plot.

Table S1. Comparison of smFISH approaches in plants.

Feature	Cryo-smFISH	Whole-mount smFISH	Paraffin-section smFISH
**Main application**	Spatial detection and quantification of single RNA molecules in tissue sections	Spatial detection and quantification of single RNA molecules in intact tissues	Spatial detection of RNAs in embedded tissue sections
**Preservation of tissue architecture**	Moderate; can be challenging in fragile tissues	Good to very good	Good morphology after embedding and sectioning
**Suitability for fragile tissues**	Limited; requires optimization and careful handling	Often advantageous, as tissue remains intact	Can be better than cryosectioning for morphology, but embedding is laborious
**Preservation of 3D tissue context**	Limited to the section plane	Strong advantage; intact tissue context maintained	Limited to the section plane
**Suitability for thick specimens**	Advantageous, because sectioning reduces out-of-focus background	More challenging due to probe penetration and background in deeper layers	Suitable after sectioning, but embedding adds processing time
**Probe / reagent penetration**	Good in thin sections	More challenging, especially in dense tissues	Good in sections after embedding
**Compatibility with immunofluorescence**	Strong advantage; antibody penetration is generally easier in sections	More difficult due to limited antibody penetration	Possible, but antigen preservation may be affected by embedding/processing
**Autofluorescence issues**	Still challenging in some tissues; far-red dyes may help	Also challenging, sometimes exacerbated by tissue thickness	Depends on tissue and processing
**Imaging requirements**	Can be imaged with widefield or confocal microscopes	Often benefits from confocal imaging, especially in thicker tissues	Widefield or confocal depending on sample and signal
**Detection of short RNAs**	Limited for short targets because multiple probes are needed	Similar limitation	Similar limitation
**Technical difficulty**	Moderate; sectioning quality is critical	Moderate to high; penetration, clearing, and mounting can be demanding	High; embedding and sectioning are time-consuming

Table S2. Cryo-smFISH strenghts and limitations.

**Strengths**	- Detection and visualization of single molecules of RNAs with cellular and sub-cellular resolution in plant tissue sections;<br>- Easy to implement and relatively fast compared to other sectioning methods;<br>- Particularly advantageous for thick specimens, where sectioning reduces out-of-focus background and can improve signal-to-noise ratio compared with whole-mount preparations;<br>- Images can be acquired with widefield and confocal microscopes;<br>- More suitable than whole-mount preparations for combined smFISH and immunofluorescence, as antibody penetration is generally more efficient in cryosections.<br>- Can be used to validate single-cell RNA seq. No transgenic lines are needed;<br>- Can be implemented in non-model species.
**Limitations**	- Tissue preservation can be challenging in fragile tissues, such as Arabidopsis roots or leaves, and often requires optimization and practice; in such cases, whole-mount approaches may better preserve tissue integrity and overall morphology.<br>- Highly autofluorescent tissues, such as adult leaves, still present challenges for detection. The signal-to-noise ratio can be improved by selecting fluorophores with emission wavelengths that minimize tissue autofluorescence. In our study, we observed that leaf autofluorescence was generally lower in the far-red range, suggesting that Quasar670 may be a more suitable choice for these tissues. However, as plant tissues contain multiple autofluorescent compounds, the optimal fluorophore selection should be carefully adjusted based on the specific tissue and its autofluorescence profile.<br>- Cannot be used to study more than 4 genes at the same time due to fluorophore limitations (i.e., only a small number of colors can be used for microscopy);<br>- Cannot be used to study RNA that are shorted than ~600nt in length (probes are 18-22 mers DNA oligonucleotides and a minimum of 25-30 probes is required per target);<br>- The probes are particularly costly, which can limit their routine use.<br>- Cryosectioning provides only a section of the tissue, whereas whole-mount approaches may better retain three-dimensional tissue context.

Table S3. smFISH probe sequences used in this study and the corresponding dyes (Quasar570 or Quasar670). Probe Sequences (5’-3’).

Probe nr	*PP2A* (Quasar570)	*HvGAPDH* (Quasar570)	*NRT1.9* (Quasar670)	*asSOFL1* (Quasar670)	*VENUS* (Quasar570)
1	ccgagcgatctatcaatcag	ggttttataaagccaggagc	caaatgtctcatttcctacc	gaccagctgaattcagtct	cggtgaacagctcctcgc
2	gacatcctcaccaaaactca	agaggtggggagggtttaag	cagattcgatgaacttccaa	cgaatccagaggctttctt	cagctcgaccaggatggg
3	tcgggtataaaggctcatca	agtgtctggaagcggatatg	cagccgtgatgctcttcat	gcaacgaaagccaccaagt	ttgtggccgtttacgtcg
4	tagctcgtcgataagcacag	cgaaggagtggaactgacgc	ccgtagatgttgacgacttt	atcacaagcgacgtttcca	ctcgccggacacgctgaa
5	ccaagagcacgagcaatgat	cgatcttaatcttgcccatg	gatggtgccaaagttgcttg	aggtccacattaaacccac	ttgccgtaggtggcatcg
6	atcaactcttttcttgtcct	atccttccgaaaccgttgat	gagtcacagaggaaagcagc	gtttctcggaggcacaatc	gtggtgcagatcagcttc
7	catcgtcattgttctcacta	tgatgaaggggtcgttgacg	aagatagggtcttgtagcgg	tccttttgtctgattccat	agggtggtcacgagggtg
8	atagccaaaagcacctcatc	catgtacgtcatgtactcgg	agaaagcaagcgatcatggc	atccacagccacacacaaa	aagcactgcaggccgtag
9	atacagaataaaaccccca	cgtgaactgtgtcgtacttg	cgaacccttttgcatcaaaca	cgagtttcttcgtaccgat	catgtggtcggggtagcg
10	caagtttcctcaacagtgga	atgtcactgtgcttccaatg	gatgcaggggatgaatcaca	tgaagctactacattgccc	tgaagaagtcgtgctgct
11	tcatctgagcaccaattcta	agcgtcttgtcgtctttgag	tatttccttagcgcattgag	gttcactccatttctggat	acgtagccttcgggcatg
12	tagccagaggagtgaaatgc	cctgacgccaaagacagtaa	tggcccattgcacacgctt	tgcttgtttgcatcactca	agaagatggtgcgctcct
13	cattcaccagctgaaagtcg	gtggactccacaacgtaatc	gaaacatgatttgtccgatg	atcctctgctgatcacatg	tcttgtagttgccgtcgt
14	ggaaaatcccacatgctgat	ttgtccttgtcagtgaagac	accagcaaaaccattgctcc	accattcctgaattgcagg	tcgaacttcacctcggcg
15	atattgatcttagctccgtc	cagagatgaccaccttcttg	atggcctaatcccaccggct	gagtcttggactagccata	ctcgatgcggttcaccag
16	attggcatgtcatcttgaca	aaacataggggcatctttgc	gatcagcaccaaatggaag	agatgtgcacgttcggtag	tgaagtcgatgcccttca
17	aaattagttgctgcagctct	ttgtcctcattgacaccaac	ttcttttgtcttcggatcga	ttcctgctagttacaagct	caggatgttgccgtcctc
18	gctgattcaattgtagcagc	atgttaacatccgaggtgta	aaactctcaatccctcgttt	aatagcatgtgcacgttcc	ttgtactccagcttgtgc
19	ccgaatcttgatcatcttgc	gtgcagctagcatttgagac	gtgaaggtgaagaaatacc	cccaaccttcacgaagatt	agacgttgtggctgttgt
20	caaccctcaacagccaataa	taatgaccttagctagggga	cgatgagggttaacgacacc	tgttattctcaggtcgctt	tgcttgtcggcggtgata
21	ctccaacaatttcccaagag	tcagaccctcaataatacca	cagctcacgtttgactgaac	aagaggaagaccctcacgt	gaagttggccttgatgcc
22	caaccatataacgcacacgc	tgatggcatgaacagtggtc	agctgggatggctaaacca	acatagctggcaactttcc	cgatgttgtggcggatct
23	agtagacgagcatatgcagg	catcaacggtcttctgtgtg	tgcaaccaagtagcattag	gcatatcctgataagcgca	gtagtggtcggcgagctg
24	gaacttctgcctcattatca	gctggggatgatgttaaagc	gagcttagaaccagcgaaga	tcttcagagacttccttct	cgatgggggtgttctgct
25	cacagggaagaatgtgctgg	cattcagctcaggaagaacc	ccactagctttaaccttgac	agttatctgcacggttctc	ttgtcgggcagcagcacg
26	tgacgtgctgagaagagtct	ccggaaagacataccggtaa	acacgagttatgctgtgaa	ttttgtagaagctgtccca	ggactggtagctcaggta
27	cccattataactgatgccaa	aactgacacatccacagtgg	cgcctcttcttgatcgcgac	attgttttcttttgccgga	ttggggtctttgctcagg
28	tggttcacttggtcaagttt	cggttctaacagtgagatca	aagctcgttaggcccaacgg	agccatgtgtaggtagttg	catgtgatcgcgcttctc
29	tctacaatggctggcagtaa	catcatatgatgcagccttc	tggcccaatttcgagttct	caatcgtgttgtttgcctt	ggtcacgaactccagcag
30	cgattatagccagacgtact	ccttgatagccttcttgatg	ttagtacctgaactgctctg	gcaatcagcttccaaagca	tccatgccgagagtgatc
31	gactggccaacaagggaata	catgatacccttgagctttc	actaagagaagtagtgagg	gtgatgattccatggcttc	
32	catcaaagaagcctacacct	accaaatcttcctcaacgta	ttggattgccgatttgtcaa	agacgccttccatggaaat	
33	ttgcatgcaaagagcaccaa	tgtcaccaacgaagtcggtg	catctttgttcagcttgtcg	acagcagttgtgagtcagg	
34	acggattgagtgaaccttgt	agccttagcatcaaagatgc	gtttccatgcatccaccggc	gaggctctgaagaggagga	
35	cttcagattgtttgcagcag	cgaaatggtcgttcagagca	ctccacttgttgcatactg	ccatggagtctccgagaaa	
36	ggaccaaactcttcagcaag	tgtcataccacgagacaagc	acccggatcacgcatttaac	cttggttttgttttcctct	
37	ggaactatatgctgcattgc	cgacaacacggttgctgtaa	taaggcggccgagagccaaa	tatgaggcttcctataggc	
38	gtgggttgttaatcatctct	ggaaagcagaacgctctact	gttgaatgtaagctaggtag	ggttgccatgtggtgaaac	
39	tgcacgaagaatcgtcatcc	catagacaaaggggcacctc	ctgaaagattgtgtaggttg	tttgtctggtagtgcttca	
40	ttactggagcgagaagcgat	gacaccatccacatttattc	aggcgtctatcggattgaag	gaagggaggcactaacctt	
41	gaacatgtgatctcggatcc	aacatgaaccaggcgtctag	ggatctggaaacttcctgga	tgtttgttcatgtgggtct	
42	ctctgtctttagatgcagtt	cagatggtaactcatgtcca	aagacggtataagaaccggc	ggcctccactgaaagaaca	
43	catcattttggccacgttaa	acttgactagcaactcggtg	tgaatatcgtcattccaagc	tgatttgcaagctgtcacc	
44	cgtatcatgttctccacaac	cacttactcaggcaaacaga	tacaaggacgcggtcataga	caaattctacacgcccact	
45	atcaacatctgggtcttcac	caaacacttactcaggctgt	gcctgtatactttctaagga	gagtccaactccattcttt	
46	ttggagagcttgatttgcga	ggatcgcagcatcaaacatc	tctctgcagttgtgtgatac	gtgacattccacagtcaca	
47	acacaattcgttgctgtctt	gacgggagtagtactcaaca	atgcatagaaacagtcctgc	cccgatgatgctttcgaat	
48	cgcccaacgaacaaatcaca	cgctccgtcctataatataa	tgcagacaccatcatactt	caagttccatccttagtct	
