# Supplementary material for: Quantitative RNA spatial profiling using single-molecule RNA FISH on plant tissue cryosections
Source: Plant Commun. 2026 Jun 8;7(8):101943. doi: 10.1016/j.xplc.2026.101943 (PMC13477017; doi:10.1016/j.xplc.2026.101943)
Supplement: Supplementary file 2 [file mmc2.pdf]

Methods S1. Step-by-step protocol of smFISH on cryosections.

Cryo-smFISH Protocol:

I. Tissue fixation and cryoprotection (bench time: 2h-4h)

1. Dissected tissues by razor blade were placed into a small glass dish (Watch Glass, Square, 1 5/8 in., Carolina, Cat.#742300) containing freshly prepared 4% PFA.
2. For root tips, after 30 mins fixation at RT, we rinse samples 3 times with 1xpbs and incubate with 34% sucrose for 45 mins at RT. For green tissues, both fixation and sucrose incubation were carried out using vacuum infiltration. PFA fixation and sucrose incubation last 2x30mins and 45 mins respectively.
3. Samples have overnight incubation with mild shaking in a mixture of equal parts 34% sucrose in 1xPBS and optimal cutting temperature compound (OCT; Leica tissue freezing medium Ref#14020108926) liquid at 4°C.

II. Sample embedding and cryosectioning (bench time: 5h-6h)

1. Replace the mixture with pre-chilled OCT and continue incubation at 4°C for 30mins-1hour.
2. Pre-cool down cryostat (Leica CM1850) to -20°C (if experiment continues step 5)
3. Transfer samples gently with forceps into 3D printed Cryomolds (1cm x 1cm x 1cm) filled with pre-chilled OCT, either insert or lie root tips in the OCT solution for generating cross or longitudinal cryosections.
4. Cryosection chunk is made through contacting cryomolds with dry ice or indirectly contacting with liquid nitrogen. (optional: Cryosection chunk were then wrapped in foil and stored at −80°C until cryosectioning; can be kept for months)
5. Transfer chunk to pre-cooled cryostat and equilibrate the temperature block at cryostat for 30mins to 1hour.
6. Section tissue in 10um thickness and transfer cryosections to polysine adhesion slides (Polysine™ adhesion microscope slides Cat. 48382-117)
7. Air-dry for up to 20 mins and store slides in the glass jar in cryostat until the sectioning is complete. (Optional stored slides at -80°C for subsequent experiments).
8. Post-fix cryosections for 10mins with 4% PFA at RT followed by gentle three-time wash with 1xPBS.

9. Samples are subjected to permeabilization step with 30mins 100% methanol, 30mins 70% ethanol followed by overnight incubation with clearsee buffer. For green tissues, clearsee incubation can be extended to one day or longer.

III. Probe hybridization (bench time: less than 30 mins)

1. Slides from clearsee buffer are placed in square plate and rinsed three times with wash buffer.
2. add 0.5-1 µl of each required probe set stock solution to 100 µl of hybridization buffer (10% dextran sulfate, 2 × SSC and 10% formamide) to reach a final concentration of 250 nM.
3. Add 100 µl of the hybridization solution to each slide, cover with a plastic coverslip(LLG Labware, 47121701 Affaldsposer) to prevent evaporation and incubate in a humid chamber at 37 °C overnight in the dark.

IV. Post-hybridization and mounting (bench time: 2hour)

1. Plastic coverslips are gently removed and hybridization solution containing unbound probes was rinsed out with 2-time wash using washing buffer.
2. slides are immersed in coplin jars containing filling wash buffer over slides about 40-50ml for 30mins to 1 hour at 37 °C.
3. DAPI(1ug/ml in wash buffer)or SR2200 dye (1ul in 50ml wash buffer) as nuclei and cell wall staining are applied for 10-15 mins and 5-10 mins respectively.
4. After 5-10 mins washing with 2x SSC buffer, samples were mounted in freshly prepared GLOX mounting medium and sealed with nail polish.

V. Imaging

Samples can be imaged using either confocal or widefield microscopy. Note: Imaging should be done ideally immediately after mounting. Signals start to decay after 1 day.

smFISH followed by immuno-fluorescence (IF)

1. After imaging of smFISH signals, the coverslips were gently removed using forceps.
2. samples were then rinsed three times with 1xPBS solution and incubated with the enzyme mix (2% Driselase, 1% Cellulase, 2% Macerozyme in 1x PBS) for 15 min in a humid chamber at 37 °C.

3. after washing three times with 1xPBS, samples were then incubated with 5% BSA blocking buffer in a humid chamber for 30 min at 37 °C.
4. samples were incubated with the H4ac antibody diluted in 5% BSA in a humid chamber overnight at 4 °C.
5. The excess antibody was washed by incubating in Coplin jars with PBST buffer at 37 °C for 30 min.
6. Samples were subjected to the incubation with secondary rabbit antibody diluted 200 times in 5% BSA in a humid chamber at 37 °C for 2 hours.
7. The excess antibody was washed at 37 °C in the dark for 30 min.
8. Samples were rinsed with 1xPBS and mounted in a drop of Vectashield medium.

Note: To re-align smFISH images with IF images, we save the coordinates of the cells detected during smFISH acquisition, ensuring that the same cells and positions are recorded in the IF experiment. However, slight sample shape shifts due to handling may sometimes make perfect re-alignment challenging.

List of Reagents and Solution:

- Tris-EDTA (PH 8.0, sigma-Aldrich, Cat.#93283-500ML)
- Nuclease-free 10× Phosphate Buffered Saline (Thermo Scientific, Cat. #AM9624)
- Nuclease-free 20× saline-sodium citrate (20× SSC, Thermo Scientific, Cat. #AM9763)
- Invitrogen™ UltraPure™ DNase/RNase-Free Distilled Water (Qiagen, Cat.#10-977-015)
- Paraformaldehyde (Sigma-Aldrich, Cat.#P6148-500g)
- Deionized Formamide (Merck, Cat.#S4117)
- Dextran Sulphate (Sigma, Cat.#D4911-100g)
- Triton X-100 (Sigma-Aldrich, Cat.#T8787)
- 1M Tris buffer pH8 (Invitrogen, Cat.#AM9856)
- Glucose oxidase (Sigma,Cat.# G2133-10KU)
- Catalase from bovine liver(Sigma, Cat.# C3155-50MG)
- RNase A, Dnase and protease-free 10mg/ml (Sigma, Cat.#EN0531)
- Sucrose (Duchefa Biochemie, Cat.# 57-50-1)
- Tissue Freezing medium (Leica, Cat.# 14020108926)
- Xylitol powder (Sigma-Aldrich, Cat.# X3375)
- Sodium deoxycholate (Sigma-Aldrich, Cat.# D6750)
- Urea (Sigma-Aldrich, Cat.# U5378)
- Vectashield mounting medium (Bionordika, Cat.# VEH-1000)
- DAPI (4′, 6-Diamidino-2-phenylindole; Sigma Cat.# D9564)
- SR2200(Renaissance Chemicals Ltd, Unit 1 Blackwood Hall Business Park, North Duffield, Selby, UK. Contact: Howard Weaver, enquiries@renchem.co.uk.)

- smFISH probe stock: Probe sets were resuspended in Tris-EDTA to a finalconcentration of 25 µM.
- 4% PFA: 2g Paraformaldehyde dissovled in 50ml 1xPBS
- 34% sucrose: 34%(m/v) sucrose dissolved in 1xPBS
- Clearsee solution: Xylitol powder10% (w/v), Sodium deoxycholate 15% (w/v) and Urea 25%(w/v) dissolved in RNase-Free Distilled Water
- Hybridization buffer(10m): 1g dextran sulfate dissolved in 1 ml nuclease free 20× SCC, 1 ml deionized formamide and nuclease free water up to 10 ml final volume.
- Wash buffer: 5 ml nuclease free 20× SSC, 5 ml deionized formamide, 50μl Triton X-100 and nuclease free water up to 50 ml final volume.
- 1ug/ml DAPI solution: 1μl 1mg/ml DAPI diluted in 1ml wash buffer.
- SR2200 solution: 1μl SR2200 in 50ml wash buffer.
- Anti-fade GLOX buffer minus enzymes(1ml): 40µl 10 % glucose in nuclease-free water, 10 µl 1 M Tris–HCl, pH 8.0 and 100 µl 20× SCC was mixed with 850 µl nuclease-free water.
- Anti-fade GLOX buffer containing enzymes(100 µl):1 µl glucose oxidase and 2 µl mildy vortexed catalase suspension added to 100 µl GLOX minus enzyme solution.
