# Supplementary material for: Quantitative RNA spatial profiling using single-molecule RNA FISH on plant tissue cryosections
Source: Plant Commun. 2026 Jun 8;7(8):101943. doi: 10.1016/j.xplc.2026.101943 (PMC13477017; doi:10.1016/j.xplc.2026.101943)
Supplement: Supplementary file 3 [file mmc3.pdf]

**Methods S2. Summary of Cryo-smFISH image analysis pipeline**

| Step | Goal | Software | Input | Output | Additional notes |
|---|---|---|---|---|---|
| **1)** Separate channels into individual images and create Z-projections | Transform raw images into standard image format | Fiji | Raw image (in the format of your microscope, for instance .czi, .lsm, .lif, .nd2, etc) | I. Single-channel multiple Z-stacks (.tiff)<br>II. Single-channel maximum Z-projection (.tiff) | • Use Fiji Macro to automate this step (suppl. )<br>• If misalignments between channels are observed, use BigWarp tool in Fiji for corrections |
| **2)** Training (optional, highly recommended) | Train a new model for tissue-specific segmentation | Cellpose | I. Pre-trained model<br>II. Single-channel maximum Z-projection [From step 1] | Model specific to tissue of interest (suppl. ) | • Perform manual annotations with 3- 5 single channel .tiff images via cellpose GUI<br>• Train a model for a batch of images through either cellpose GUI, or cellpose CLI<br>• More information can be found in Cellpose user guide |
| **3)** Cell and nuclear segmentation | Define cell and nuclear areas | Cellpose | I. Single-channel maximum Z-projection [From step 1]<br>II. Model specific for tissue of interest [From step 2] | Binary mask image (.png) | Use the cellpose CLI for batch segmentation (suppl. ) |
| **4)** Extraction of the cell outline coordinate information | Create a text file with the cell and nuclear outline coordinates for FISHquant | FISHquant (Cell segmentation tool) | Binary mask image [From step 3] | Outline text file (.txt) | |
| **5)** Pre-detection of smFISH signal | Define the parameters for image filtering and dot detection in one representative image | FISHquant | Outline text file [From step 4] and single-channel multiple Z-stacks [From step 1] | Detection setting text file (.txt) | We recommend adapting these parameters for each fluorophore, gene, and tissue. An example file for the parameters used in our study is available (suppl.) |

| | | | | | |
|---|---|---|---|---|---|
| **6)** Batch detection of smFISH signal | Apply the previously defined settings for the batch image analysis | FISHquant (Batch analysis tool) | Detection setting text file [From step 5] and single-channel multiple Z-stacks [From step 1] | Summary table containing the number of detected RNA molecules per cell per image (.txt)<br><br>Table containing the coordinates of each detected dot per image (.txt) | |
| **7)** Image visualization with detected RNA dots | Create images with the cell outlines that clearly display the detected RNA dots | Python script 1, CellProfiler, Adobe Illustrator, Fiji | Binary mask image [From step 3] and dot coordinates table [From step 6] | Overlay image | • Python script for creating the image is available (suppl.)<br>• CellProfiler pipeline available (suppl.) |
| **8)** Protein detection | Measure the fluorescence intensity per cell | CellProfiler | Single-channel Z-projection [From step 1] and Binary mask PNG [From step 3] | Summary table containing the fluorescence intensity per cell per image | CellProfiler pipeline available (suppl.) |

**<u>Step-by-step guideline</u>**

1) Single-channel image separation
    a. Open Fiji
    b. *Plugins -> Macros -> Run…*
    c. Select the macro
    d. Select the folder containing the raw images
2) Training (optional) [We recommend following the developer's instructions at https://cellpose.readthedocs.io/en/latest/]
    a. Open Anaconda and activate the virtual environment where cellpose is installed
    b. Using cellpose GUI:
        I. Launch Cellpose GUI
        II. Apply one of the pretrained models that adjust better to the tissue structure

III. Correct manually the errors in the segmentation
IV. Train a new model (*Models -> Train new model with image+masks in folder*)
V. Repeat the process for 3-5 images until getting the desired segmentation accuracy

c. Alternatively, train via command line:
   I. Launch cellpose
   II. Run command line A

3) Cell and nuclear segmentation
   a. Open anaconda
   b. Launch cellpose
   c. Run the segmentation via command line B for batch analysis.

4) Cell outline file writing
   a. Open MATLAB
   b. Run FISHquant
   c. *Tools -> Cell segmentation*
   d. In the Cell profiler: generate FQ outlines from segmentation, fill the proper information:
      i. Define experimental parameters
      ii. Indicate file identifiers
      iii. Define folder
      iv. Create FQ outlines

5) Pre-detection of smFISH signal
   a. Open MATLAB
   b. Run FISHquant
   c. *[FQ] Main -> Load -> Cell outline* (choose the text file with the outline coordinates of a representative image)
   d. Define the filtering settings
      i. Recommended:
         1. Use the 3D_2xGauss method
         2. Kernel BGD: 5,Kernel SNR size: 0.5

         Note:

         - The Kernel BGD value indicates the degree to which the image will be blurred to detect and subtract the background. Increasing this value will subtract more background.

- The Kernel SNR value indicates the extent to which the remaining small signals in the image (after background subtraction) will be enhanced. A small value here is recommended when it is necessary to separate small dots that are close together.
- These parameters may need to be adjusted based on the microscope setup and sample. We recommend verifying the accuracy of detection from the next step on cells with dense RNA region or high background before proceeding with batch detection, and change again these parameters if needed. Keeping in mind that some errors in detection are normal due to nonspecific high-fluorescence signals or occasional missed signals caused by the shape of the detected dots.

e. Pre-detection
    i. Define the intensity threshold close to the elbow of the curve
    ii. Check the detection accuracy in the Visualization tool
    iii. Set a quality score
    iv. Apply the Pre-detection in all cells in the image
f. Fit spots
g. Threshold parameters if needed
    i. Note:
h. Thresholding the parameters (Sigma, Amplitude, Background, Pixel Intensity) can be done more easily after batch processing. The more dots that are detected, the easier it is to determine the normal distribution for each parameter. At this point, we recommend prioritizing the detection of all RNA molecules with clear signals, while keeping the number of false positives low. Save the detection settings

6) Batch detection of smFISH signal
    a. Open MATLAB
    b. Run FISHquant
    c. *Tools -> Batch processing*
    d. Define file with setting
        i. Press “Load” and choose the previously saved detection setting generated in step 5.
        ii. Add outlines. Add all the outline coordinate text file for all the images to analyze.
        iii. Press PROCESS button
        iv. Threshold the parameters based on normal distributions
        v. Export the results
            1. For count of molecules per cell: *[FQ] Batch -> Save -> Summary: mature mRNA*

    2. For dot coordinates per image: *[FQ] Batch -> Save -> Summary: of THRESHOLDED spots*

7) Image visualization with detected RNA dots
    a. Overlay detected RNA dots onto a blank image using python script 1
    b. Export cell or nuclear contour outlines using CellProfiler pipeline :
        I. Open CellProfiler
        *II.* Go to *File -> Import -> Pipeline from file*
        III. Drop the images into the program in “Images”
        IV. Check the strings for image detections in “NamesAndTypes”
        V. Press Analyze Images
    c. Vectorize the cell /nuclear outline image from step 6:
        I. Open the exported image in Adobe illustrator
        II. Use the Image Trace feature to vectorize the image
        III. Adjust the threshold settings to make the protophloem size element contour thicker for better visibility
    d. Overlay RNA and mask image using Fiji
8) Protein detection
    a. Open CellProfiler
    *b.* Go to *File -> Import -> Pipeline from file*
    c. Drop the images into the program in “Images”
    d. Check the strings for image detections in “NamesAndTypes”
    e. Press Analyze Images
