# Supplementary material for: Quantitative RNA spatial profiling using single-molecule RNA FISH on plant tissue cryosections
Source: Plant Commun. 2026 Jun 8;7(8):101943. doi: 10.1016/j.xplc.2026.101943 (PMC13477017; doi:10.1016/j.xplc.2026.101943)
Supplement: Supplementary file 4 [file mmc4.pdf]

Plant Communications
Resource article

CellPress Partner Journal

Quantitative RNA spatial profiling using single-molecule RNA FISH on plant tissue cryosections

Xue Zhang[1,3,4], Alejandro Fonseca[1,4], Konstantin Kutashev[1], Martina Leso[1], Adrien Sicard[1], Susan Duncan[2] and Stefanie Rosa[1,*]

[1]Department of Plant Biology, Swedish University of Agricultural Sciences (SLU), Uppsala, Sweden

[2]Department of Cell and Developmental Biology, John Innes Centre, Norwich Research Park, Norwich NR4 7UH, UK

[3]Present address: Science for Life Laboratory, Division of Genome Biology, Department of Medical Biochemistry and Biophysics, Karolinska Institute, Stockholm, Sweden

[4]These authors contributed equally to this article.

***Correspondence: Stefanie Rosa (stefanie.rosa@slu.se)**

https://doi.org/10.1016/j.xplc.2026.101943

ABSTRACT

Single-molecule fluorescence *in situ* hybridization (smFISH) has emerged as a powerful tool for studying gene expression dynamics with unparalleled precision and spatial resolution in a variety of biological systems. Recent advancements have expanded its application to encompass plant studies, yet there remains a need for a simple and robust smFISH method adapted to plant tissue sections. Here, we present an optimized smFISH protocol, termed cryo-smFISH, for visualizing and quantifying single mRNA molecules in plant tissue cryosections. This method exhibits remarkable sensitivity, enabling the detection of low-expression transcripts, including long non-coding RNAs. By integrating a deep learning-based algorithm into our image analysis pipeline, our method enables precise assignment of RNA abundance in nuclear and cytoplasmic compartments. The method also enables robust integration with immunofluorescence, as cryosectioning enhances antibody penetration. This allows for the sequential visualization and quantification of both RNAs and endogenous proteins within the same cells. Finally, this study demonstrates the use of smFISH to validate single-cell RNA sequencing (scRNA-seq) expression patterns in plant tissues. By extending smFISH to plant cryosections, plant scientists will be able to exploit the full potential of quantitative transcript analysis at cellular and subcellular resolution.

Key words: smFISH, scRNA-seq, smFISH–IF, tissue cryosections, quantitative gene expression

Zhang X., Fonseca A., Kutashev K., Leso M., Sicard A., Duncan S., and Rosa S. (2026). Quantitative RNA spatial profiling using single-molecule RNA FISH on plant tissue cryosections. Plant Comm. **7**, 101943.

INTRODUCTION

High-resolution analysis of spatiotemporal gene-expression dynamics requires precise quantification of RNA abundance and spatial distribution within cells and tissues. RNA *in situ* hybridization (ISH) is a classical technique that enables researchers to map RNA molecules within intact cells or tissues with spatial precision. This is achieved through the use of probes containing nucleic acid sequences that are complementary to a specific target RNA sequence (Pardue and Gall, 1969; Cox et al., 1984). ISH probes can be labeled with a variety of detectable molecules, such as radioisotopes (Harrison et al., 1973), biotin (Hutchison et al., 1982; Brigati et al., 1983), digoxigenin (Heiles et al., 1988; Panoskaltsis-Mortari and Bucy, 1995), and fluorescent dyes (Bauman et al., 1980; Singer and Ward, 1982). However, traditional ISH techniques suffer from limited spatial resolution and are primarily qualitative in nature.

Single-molecule fluorescence *in situ* hybridization (smFISH) is a variant of FISH that enables single-molecule resolution (Femino et al., 1998; Raj et al., 2008). It uses a set of 25–48 short DNA oligonucleotides (18–22-mers) to bind specific sequences in the target RNA, enabling the visualization and quantification of individual RNA molecules within intact cells and tissues at high spatial resolution. While smFISH has been successfully used in a variety of model organisms, including bacteria (Skinner et al., 2013), *C. elegans* (Raj et al., 2008), and mammalian cells (Lyubimova et al., 2013), its application to diverse plant tissues has been constrained by technical complexities arising from inherent autofluorescence and light-scattering properties of cell walls and vacuole-rich tissues. The adaptation of smFISH to the plant model species *Arabidopsis thaliana* in 2016 marked a pivotal development, enabling the imaging of individual RNA molecules within fixed, squashed root meristem cells (Duncan et al., 2016; 2017). Since then, additional smFISH protocols and other

 © 2026 The Author(s). Published by Elsevier Inc. on behalf of CAS Center for Excellence in Molecular Plant Sciences, Chinese Academy of Sciences, and Chinese Society for Plant Biology. This is an open access article under the CC BY license (http://creativecommons.org/licenses/by/4.0/).

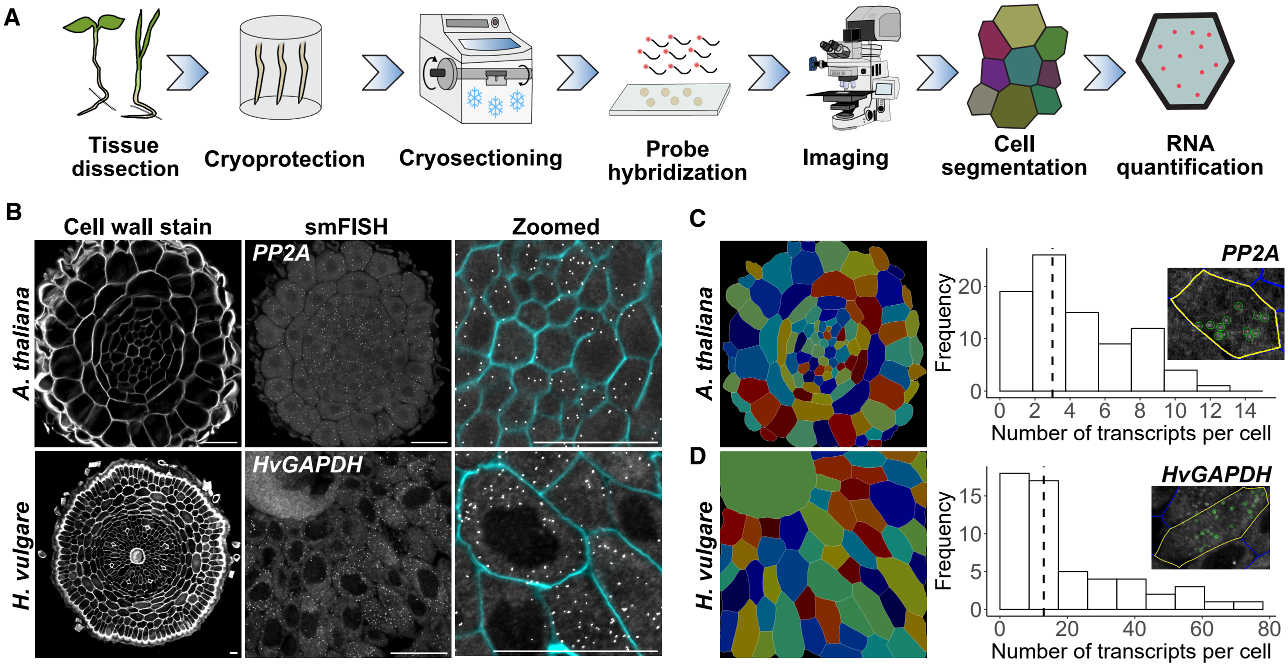

Figure 1. RNA detection and quantification in *Arabidopsis* and barley roots using cryo-smFISH.
(A) Schematic illustration of the cryo-smFISH detection and quantification workflow.
(B) Single-plane confocal images of root cross-sections showing the detection of *PP2A* mRNA in *Arabidopsis* and *HvGAPDH* mRNA in barley. Left: cell contours visualized by cell wall staining with Renaissance 2200; middle: smFISH images with discrete white dots corresponding to individual RNA molecules; right: zoomed-in image showing detected transcripts (white dots) and cell walls (cyan). Scale bars, 20 μm.
(C and D) Segmented cell masks for *Arabidopsis* **(C)** and barley **(D)** cryosections generated by Cellpose (left). Histograms show FISH-quant-based quantification of cryo-smFISH signals per cell within the imaged volume for *PP2A* **(C)** and *HvGAPDH* **(D)**, corresponding to the images shown in **(B)**. The dashed line indicates the median of the distribution. The inset images show examples of RNA detection in individual cells.

newly developed ISH-based methods have gradually emerged for studying gene expression in plants (Solanki et al., 2020; Yang et al., 2020; Huang et al., 2023; Nobori et al., 2023). We recently optimized smFISH for whole-mount intact plant tissues (Zhao et al., 2023). Despite the success of whole-mount smFISH in several tissues, this method is not ideal for very thick specimens, and physical sectioning may be required for thick plant samples. smFISH has been applied to plant tissue sections from paraffin-embedded samples (Huang et al., 2020). However, because paraffin embedding involves several steps and can be time-consuming, a more rapid and streamlined smFISH method for plant tissue sections would be beneficial.

In this study, we present an optimized smFISH protocol for the visualization and quantification of single RNA molecules in plant tissue cryosections. This method enables quantitative analysis of weakly expressed transcripts, such as long non-coding RNAs (lncRNAs), and provides precise information on transcript distribution across diverse cell types. By incorporating DAPI staining and a deep learning-based cell-segmentation pipeline, we achieved segmentation of subcellular compartments, allowing us to distinguish and quantify nuclear and cytoplasmic transcripts. Additionally, performing smFISH and immunofluorescence (IF) sequentially enables the visualization and quantification of RNAs and endogenous proteins within individual cells. Notably, this study demonstrates the application of smFISH for single-cell RNA sequencing (scRNA-seq) validation in plants, enabling the investigation of spatial gene-expression patterns in relation to cell identity. In summary, our study illustrates how smFISH can be used for scRNA-seq validation and for the simultaneous detection of endogenous RNAs and proteins.

RESULTS

Single-molecule RNA detection and quantification in *Arabidopsis* and barley tissue cryosections

Imaging plant tissues presents specific challenges because of their inherent autofluorescence. Although clearing techniques have greatly improved our ability to observe plant tissues in whole-mount settings (Zhao et al., 2023), their application remains limited in some plant species due to tissue thickness. In this context, tissue sectioning represents a valuable alternative. In contrast to optical sectioning, physical sectioning allows thicker specimens to be imaged without sacrificing the signal-to-noise ratio. The first step in developing our smFISH protocol for cryosections (hereafter referred to as cryo-smFISH) involved obtaining high-quality sections that preserve both plant tissue morphology and RNA integrity. To accomplish this, we adapted previously published protocols (Anjam et al., 2016; Stapel et al., 2018) for use in *A. thaliana* (*Arabidopsis*) root cryosections (Figure 1A). This included a cryoprotection step before cryosectioning to preserve tissue morphology (Supplemental Figure 1) and a re-fixation step to ensure RNA preservation. These adjustments, combined with the use of 5–10-μm-thick sections, enabled us to obtain well-preserved samples consisting of

a single layer of cells. Although *Arabidopsis* roots are not thick specimens, they are extremely fragile, posing significant challenges for preserving tissue structure. Therefore, optimizing a cryosectioning protocol tailored to *Arabidopsis* roots provides a valuable initial step in developing a robust method that can be applied to other tissue types. To optimize and validate our cryo-smFISH protocol in *Arabidopsis* root cryosections, we focused on detecting transcripts from the housekeeping gene *Protein Phosphatase 2A* (*PP2A*), as previously reported (Duncan et al., 2016; 2017; 2023; Zhao et al., 2023). The resulting cryo-smFISH images from both longitudinal and transverse cryosections revealed distinct bright puncta corresponding to *PP2A* mRNA molecules, visible by both epifluorescence and confocal microscopy (Supplemental Figure 2), with confocal imaging providing clearer cell outlines. *PP2A* mRNA molecules displayed a ubiquitous distribution across various root cell types in the root meristem region, consistent with previous reports (Duncan et al., 2016; Zhao et al., 2023). To enable precise allocation of transcripts to individual cells, we stained cryosections with the cell wall dye Renaissance 2200 (SR2200) (Musielak et al., 2016; Zhao et al., 2023). Combined with our integrated image analysis pipeline (Zhao et al., 2023), this enabled the precise quantitative assignment of *PP2A* mRNA molecules to individual cells (Figures 1B and 1C; Supplemental Figure 3). Treatment with RNase A confirmed that the observed signals corresponded to true mRNA molecules (Supplemental Figure 4). It is important to note that cryosections capture only a fraction of a cell's total 3D volume. Because many plant cells are highly elongated, we do not infer absolute "whole-cell" transcript numbers; instead, we report the number of transcripts detected per segmented cell within the imaged section as a proxy for relative expression. We empirically validated this two-dimensional (2D) sampling approach by demonstrating a highly significant correlation between transcript counts in adjacent optical slices of the same cells (Supplemental Figure 5). Nevertheless, if transcripts are unevenly distributed within cells, especially in cells longer than 10 μm, cross-sectional analyses should be complemented with longitudinal sections to accurately inspect transcript distributions.

We then assessed the applicability of our cryo-smFISH protocol in *Arabidopsis* leaves. Despite the challenges associated with autofluorescence in plant tissues, we were able to detect *PP2A* RNA signals in young *Arabidopsis* leaves (Supplemental Figure 6). We also attempted detection in adult *Arabidopsis* leaves, where high autofluorescence resulting from abundant chloroplasts, together with difficulties in preserving the spongy mesophyll structure, makes the process considerably more difficult. To mitigate autofluorescence, we used probes in the far-red region (Quasar670) and applied extended clearing steps (2 weeks with ClearSee). Under these conditions, RNA signals were detectable (Supplemental Figure 7); however, signal detection was inconsistent across cells, highlighting the technical limitations of the method in this tissue. We further sought to expand the application of cryo-smFISH to other plant species. Specifically, we applied our approach to the monocot *Hordeum vulgare* (barley) by detecting the housekeeping gene *HvGAPDH* in cryosections of roots and young leaves. *HvGAPDH* transcripts were clearly detected and quantifiable in both tissues (Figures 1B and 1D; Supplemental Figures 8A and 8B). Consistent with our earlier analyses, treatment with RNase A confirmed the specificity of the RNA signals (Supplemental Figure 8C). We further validated the specificity of the assay through cross-probe control experiments, using probes designed to detect *PP2A* from *Arabidopsis* and *GAPDH* from barley in both sample types (Supplemental Figure 9), and by using probes against VENUS mRNA in a *VENUS* transgenic line compared with wild type (Supplemental Figure 10). These results demonstrate that cryo-smFISH can be successfully used to detect and quantify specific RNA molecules with high resolution in cryosections obtained from both model and crop plant tissues.

Mapping the cell type distribution of transcripts in *Arabidopsis* roots using cryo-smFISH

scRNA-seq has emerged as a powerful genomic approach for the detection and quantitative analysis of messenger RNA molecules at single-cell resolution. Early scRNA-seq studies using high-throughput droplet-based technologies have revolutionized our understanding of plant biology by revealing the transcriptional states of many different cell types (Denyer et al., 2019; Zhang et al., 2019; Wendrich et al., 2020; Shaw et al., 2021; Otero et al., 2022; Shahan et al., 2022). However, gene expression estimates derived from scRNA-seq are not absolute but are influenced by capture efficiency, normalization strategies, and computational inference. Hence, complementary methods are needed to validate scRNA-seq results. Furthermore, scRNA-seq does not preserve spatial information, as tissue is disintegrated into individual cells before analysis (Shaw et al., 2021; Bawa et al., 2022). Complementary techniques, such as transgenic reporter lines and traditional ISH methods, are often utilized for scRNA-seq validation. Yet, these methods lack quantitative precision, fail to capture RNAs with particularly low expression, cannot visualize active transcription events, and are unsuitable for determining kinetic transcriptional parameters. These limitations highlight the need for complementary techniques that can validate scRNA-seq findings while retaining cellular spatial information. Here, we demonstrate the use of cryo-smFISH to map the spatial distribution of transcripts in plant tissues as a method suitable for scRNA-seq validation. As a proof-of-principle, we chose to study the nitrate transporter gene *NRT1.9* (*AT1G18880*), which has been implicated in facilitating nitrate loading into phloem cells in *Arabidopsis* roots (Wang and Tsay, 2011). To investigate the cell-type-specific expression of this gene, we reanalyzed scRNA-seq data from 10-day-old *Arabidopsis* root tips, as previously described (Wendrich et al., 2020). Following clustering and cell type annotation, we subset the dataset to specifically investigate cell types readily distinguishable in root cryosections: epidermis, cortex, endodermis, pericycle, phloem, procambium, and xylem (Figure 2A). Analysis of differential gene expression (DGE) among these cell types indicated that *NRT1.9* is preferentially expressed in the phloem, pericycle, and procambium (Figures 2A and 2B). To confirm these results, we designed a mixed smFISH probe set targeting both exonic and intronic regions of NRT1.9 RNA and performed cryo-smFISH on *Arabidopsis* root cross-sections (Figures 2C and 2D). The cryo-smFISH results closely aligned with the scRNA-seq expression pattern for *NRT1.9* (Figures 2B–2F). However, a discrepancy was observed in the procambium, where scRNA-seq indicated

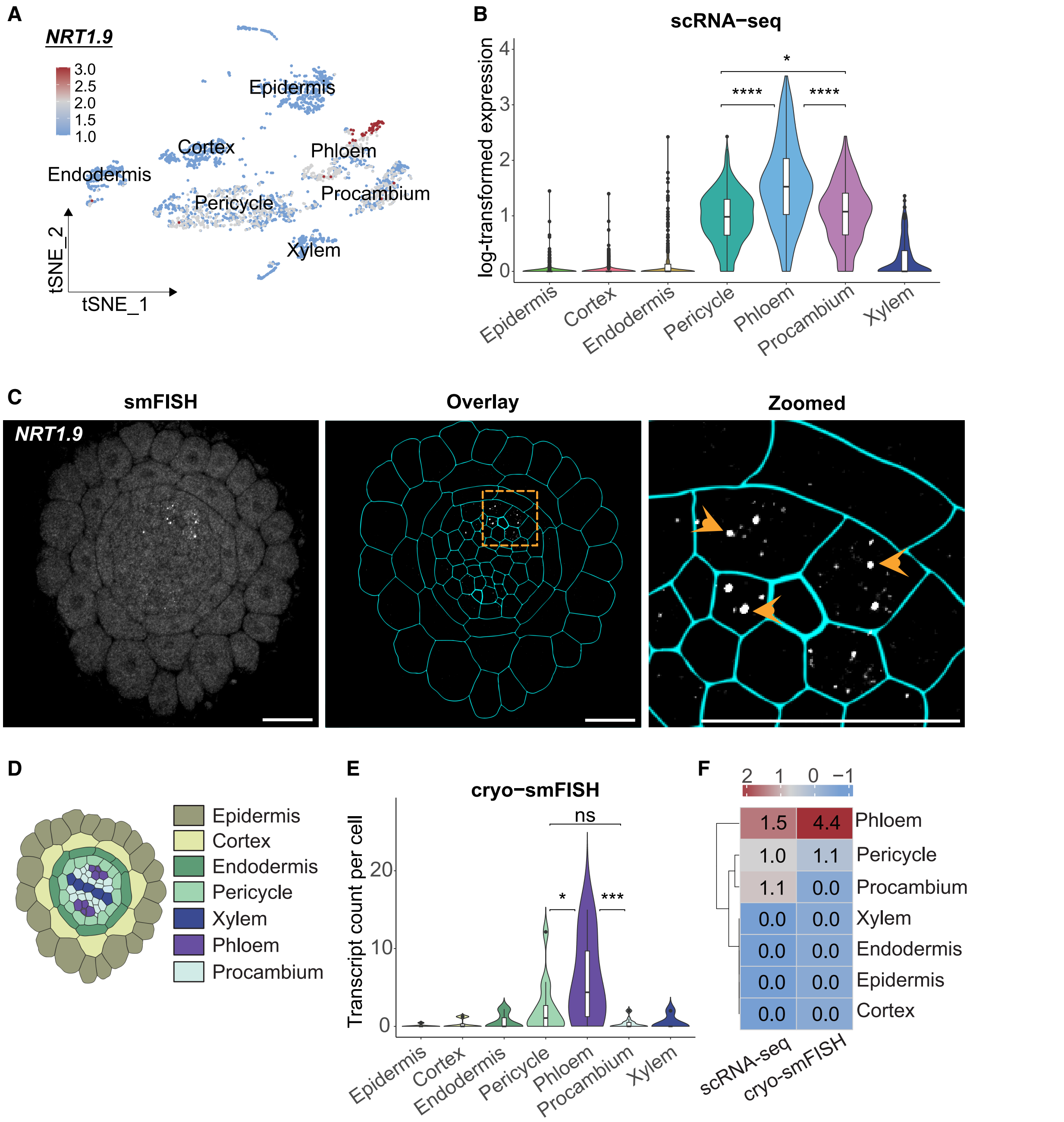

Figure 2. Mapping the cellular distribution of NRT1.9 transcripts in *Arabidopsis* roots using scRNA-seq and cryo-smFISH.
(A) t-SNE (t-distributed stochastic neighbor embedding) visualization of cell clusters and *NRT1.9* expression patterns detected by scRNA-seq (GEO: GSE141730).
(B) Quantification of NRT1.9 transcript levels per cell across different cell types based on scRNA-seq data (GEO: GSE141730).
(C) Representative single-plane confocal images of cryo-smFISH showing *NRT1.9* transcripts in an *Arabidopsis* root cross-section. Left: single-plane confocal image of cryo-smFISH for *NRT1.9*; middle: cell outlines obtained by segmenting the SR2200-stained cell wall signal (cyan) and NRT1.9 RNA smFISH signals (white); right: zoomed-in image showing *NRT1.9* transcripts (white dots) and cell walls (cyan). Orange arrows indicate bright NRT1.9 RNA foci. Scale bars, 20 μm.
(D) Schematic representation of cell types in the *Arabidopsis* root cross-section shown in **(C)**.
(E) The violin plot illustrates the quantification of transcripts per cell within the imaged volume from the cryo-smFISH image shown in **(C)** ($n = 86$ cells). Four replicates from the same developmental stage showed similar results.

(legend continued on next page)

higher levels of expression. Nevertheless, expression trends across the other cell types were highly similar between the two approaches, with phloem cells consistently exhibiting higher levels of expression in both scRNA-seq and cryo-smFISH analyses (Figure 2F). Detailed inspection of the images revealed RNA foci of higher intensity (Figure 2C, indicated by orange arrows). Through DAPI staining, we confirmed that these brighter foci were predominantly present in the nucleus (Supplemental Figure 11). Importantly, treatment with RNase A once again confirmed the specificity of the detected RNA signals (Supplemental Figures 12A and 12B). Overall, these results highlight the valuable application of cryo-smFISH as a method to validate and complement scRNA-seq findings at both qualitative and quantitative levels.

Detection of long non-coding RNA distribution with subcellular resolution

Long noncoding RNAs (lncRNAs) are a class of RNA molecules longer than 200 nucleotides that have garnered increasing interest due to their potential regulatory roles in various biological processes (Jha et al., 2020; Lucero et al., 2020; Statello et al., 2021). In contrast to coding RNAs, lncRNAs are recognized for their pronounced tissue specificity and, often, relatively lower abundance (Djebali et al., 2012; Wang et al., 2014; Cabili et al., 2015; Rosa et al., 2016; Zhao et al., 2018). Despite their predicted contributions to multiple aspects of gene regulation, with subcellular localization being key to their function (Statello et al., 2021), the study of lncRNAs, especially at the single-cell level, remains limited (He et al., 2023). Here, we tested whether our cryo-smFISH method could be used to detect an uncharacterized low-abundance lncRNA. *asSOFL1* is the antisense lncRNA of *SOFL1* (Kim et al., 2022). We reanalyzed bulk RNA-seq data from wild-type *Arabidopsis* root tips (Choe et al., 2017). Among other genes, *asSOFL1* RNA was found to be weakly expressed but detectable in root tips. We next designed smFISH probes targeting *asSOFL1* transcripts and performed cryo-smFISH on *Arabidopsis* cross-sections. The results revealed bright dots corresponding to *asSOFL1* transcripts (Figure 3A and Supplemental Figure 13). Counting transcripts per cell revealed that this lncRNA is expressed at low levels, with only a few transcripts detected per segmented cell within the section (Figure 3B). Despite its low expression, *asSOFL1* transcripts were found ubiquitously across various cell types within the root (Figure 3C). We then employed RNase A treatment, which confirmed that the bright spots observed corresponded to true RNA signals (Supplemental Figure 12C). We additionally compared the expression profiles of *asSOFL1* across root cell types obtained from scRNA-seq, bulk RNA-seq, and cryo-smFISH data (Supplemental Figure 14). This comparative analysis revealed that the results obtained by cryo-smFISH are consistent with those from the other two methods, with similar longitudinal trends also observed for *NRT1.9* and *PP2A*.

Subcellular localization patterns of lncRNAs can provide insight into their function (Cabili et al., 2015). To elucidate the subcellular localization of *asSOFL1* transcripts, we incorporated DAPI staining into our cryo-smFISH protocol. Visual inspection of the images revealed that *asSOFL1* transcripts were predominantly localized in the nucleus (Figures 3D and 3E). To obtain a quantitative description of both nuclear and cytoplasmic compartments, we modified the cell-segmentation step in our image-analysis pipeline. This was achieved using custom-trained Cellpose models (Stringer et al., 2021), a deep learning-based algorithm for cellular segmentation. Using two models trained specifically for segmenting cells and nuclei in *Arabidopsis* and barley tissue cryosections, we created cell masks and nuclear masks required as input files for subsequent transcript quantification using the FISH-quant pipeline. With this adapted image-analysis pipeline, we were able to automatically assign transcripts to either nuclear or cytoplasmic compartments. Our cryo-smFISH results showed that most *asSOFL1* transcripts were localized in the nucleus (Figures 3F and 3H; Supplemental Figure 13). In contrast, over 70% of *PP2A* transcripts were found in the cytoplasm, where translation occurs, as expected for a coding gene (Figure 3G). To further characterize these subcellular populations, we quantified the raw fluorescence intensity of individual smFISH foci. For both genes, cytoplasmic signals displayed a uniform intensity distribution characteristic of single, mature RNA molecules. Conversely, nuclear foci showed significantly higher variance (Levene's test, $p < 0.05$) and a distinct subpopulation distribution (Welch's test, $p < 0.05$), with high-intensity outliers corresponding to putative transcription sites where multiple nascent transcripts accumulate (Supplemental Figure 15). Together, these results illustrate that our cryo-smFISH protocol can be used to detect low-abundance transcripts, such as lncRNAs, and provide important data on their quantitative expression at the cellular and subcellular levels.

Simultaneous RNA and protein detection using a sequential cryo-smFISH/IF protocol

Examining the subcellular localization of proteins in conjunction with gene expression quantification and mRNA localization can shed light on the complex interplay between transcriptional and post-translational regulatory processes. To establish the proof-of-principle technical compatibility between cryo-smFISH and IF, we adapted a sequential smFISH-IF protocol as previously described (Rosa et al., 2016). Using probes against *PP2A* mRNA, we first performed cryo-smFISH, followed by IF using an antibody against the histone marker H4Ac. The results revealed that antibody staining was compatible with the cryo-smFISH procedure, with uniform signals across all cells and good tissue preservation, allowing straightforward overlay of cryo-smFISH and IF images (Figure 4A). This dual approach allowed us to visualize *PP2A* mRNA and H4Ac in the same cells and to quantify their levels per cell within the section (Figures 4B and 4C), as well as to assess their abundance

(F) Heatmap showing that both scRNA-seq and cryo-smFISH identified *NRT1.9* as highly expressed in phloem cells. The values displayed in the heatmap represent the median transcript levels within each cell type, normalized by cell number.

Box plots within the violin plots show the interquartile range (IQR; 25%–75%), with the median indicated by a horizontal line. Whiskers show the ±1.58 × IQR value. Statistical analyses were performed using one-way ANOVA followed by Tukey's honestly significant difference (HSD) tests. A p value greater than 0.05 indicates no statistical significance (ns), while p values less than 0.05, 0.001, and 0.0001 are denoted as *, ***, and ****, respectively. In **(B) and (E)**, only statistical comparisons among pericycle, phloem, and procambium are shown.

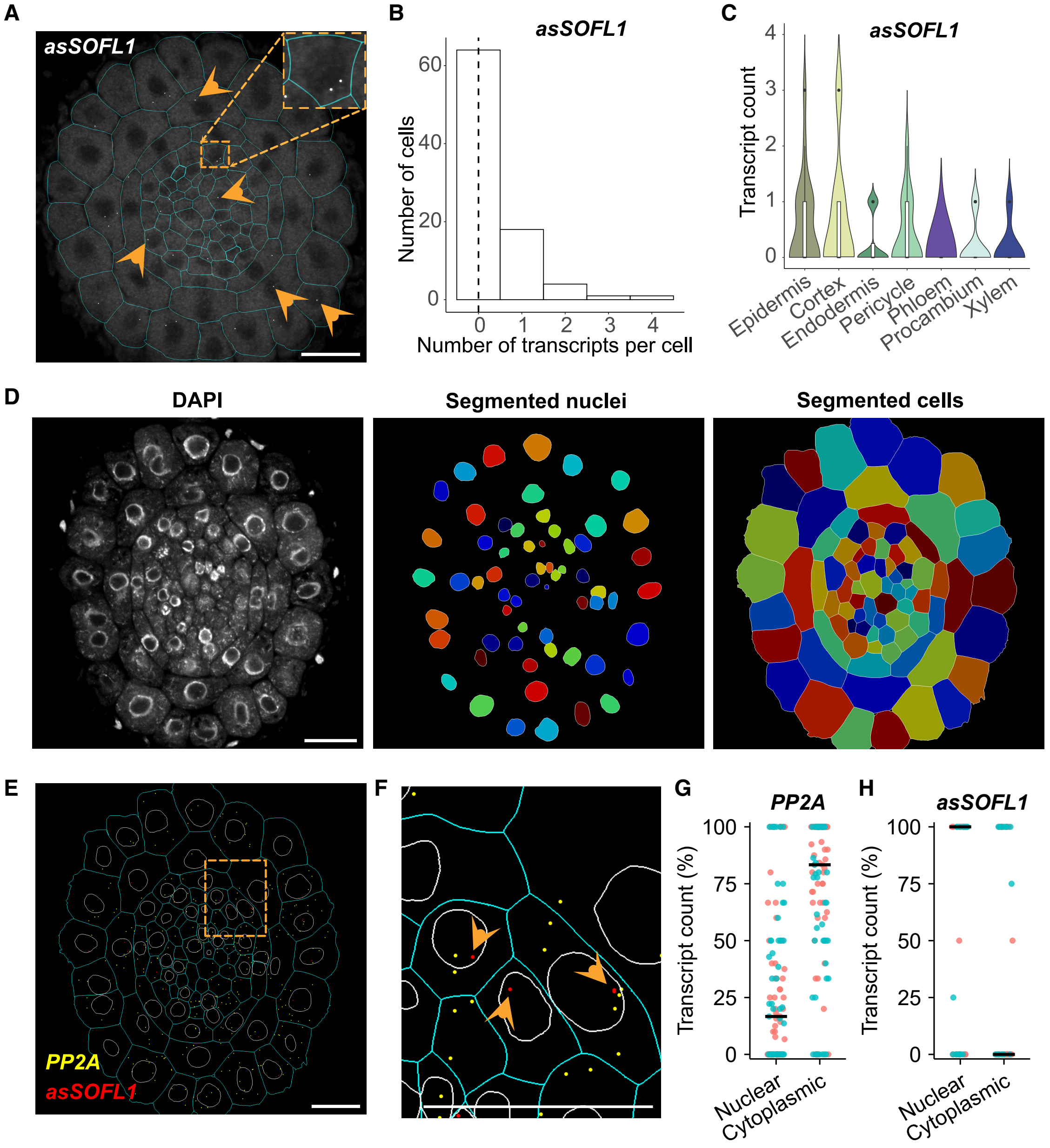

Figure 3. Cryo-smFISH enables detection and quantification of long non-coding RNAs with subcellular resolution.

(A) Representative single-plane confocal image showing *asSOFL1* transcripts (white) in an *Arabidopsis* root cross-section, with cell outlines (cyan) obtained by segmenting the signal from the cell wall dye SR2200. Orange arrowheads indicate *asSOFL1* RNA signals.

(B) Histogram showing the distribution of *asSOFL1* transcript numbers per cell within the imaged volume, derived from the image shown in **(A)**. The dashed line indicates the median transcript number.

(C) Violin plot showing the quantification of *asSOFL1* transcripts across different cell types, derived from the image shown in **(A)**. Experiments were repeated independently five times. Statistical differences between cell types were evaluated using a one-way ANOVA followed by Tukey's honestly significant difference (HSD) test, with no statistically significant differences observed ($p > 0.05$). Boxes inside the violin plots show the interquartile range (IQR, 25%–75%), with median values represented by horizontal lines. Whiskers denote $\pm 1.58 \times$ IQR.

(D) Representative example of image segmentation using the DAPI signal (left) to generate nuclear masks (middle) and cellular masks (right) with a custom Cellpose model.

(E) Detection of *asSOFL*1 (red dots) and *PP2A* mRNA (yellow dots) using our image analysis pipeline. Cyan and white lines represent cellular and nuclear outlines, respectively.

(F) Zoomed-in image of the boxed region in **(E)**. Orange arrowheads indicate *asSOFL1* RNA signals.

(G and H) Jitter plots showing the percentage of *PP2A* (G, $n = 107$ cells) and *asSOFL1* (H, $n = 35$ cells) transcripts localized to nuclear *vs.* cytoplasmic regions. Each dot represents an individual cell, with colors distinguishing the two independent replicates. Black horizontal lines represent median values. Scale bars, 20 μm.

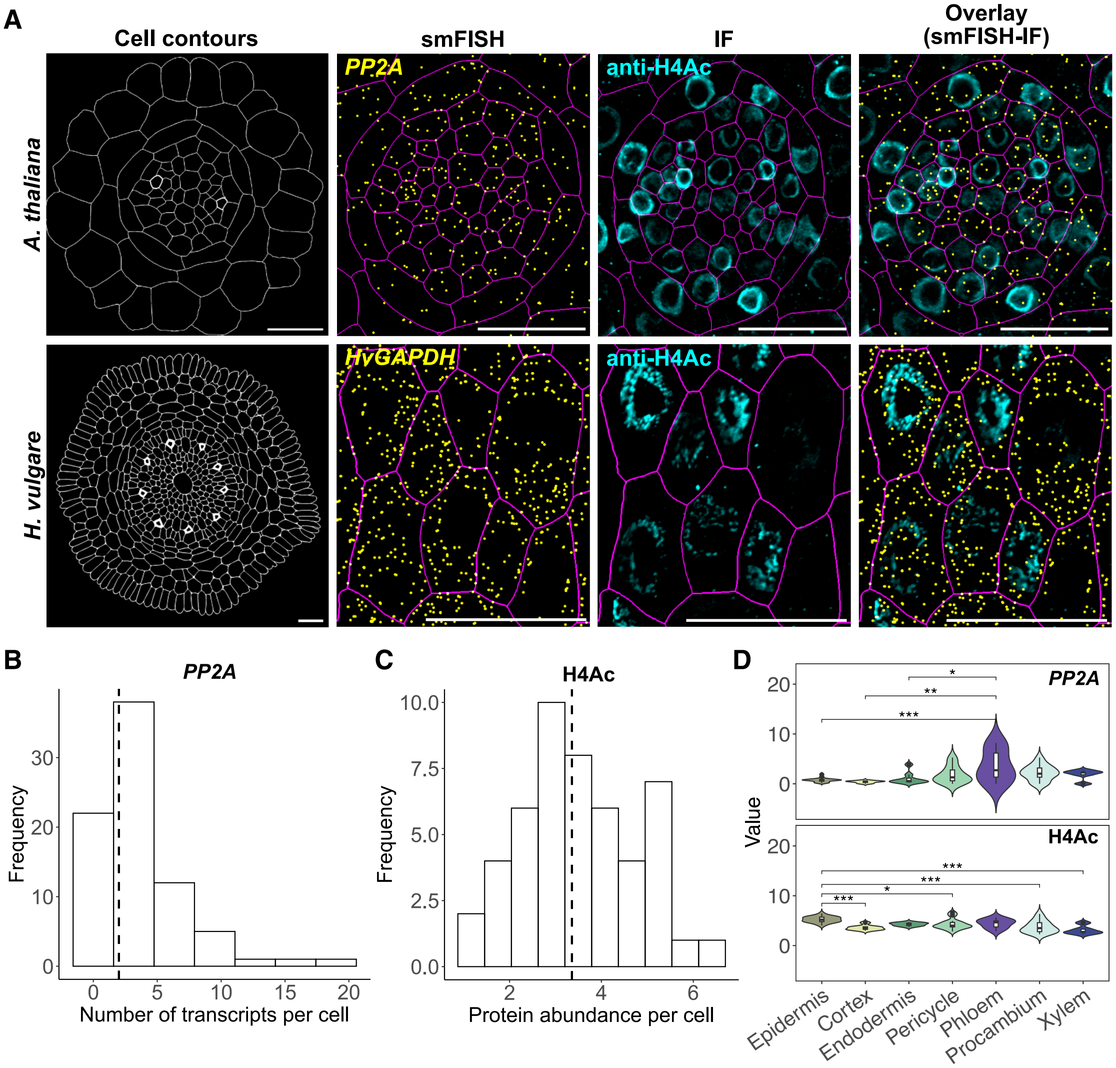

Figure 4. A sequential cryo-smFISH–IF protocol enables the simultaneous detection and quantification of RNAs and endogenous proteins in single cells.

(A) Representative single-plane confocal images obtained using a sequential cryo-smFISH–IF protocol on *Arabidopsis* and barley cross-sections. Shown are cell wall outlines (white or magenta) obtained by segmenting the signal from the cell wall dye SR2200; detected *PP2A* and *HvGAPDH* transcripts (yellow); and acetylated histone H4 (H4Ac) signal (cyan). Scale bars, 20 μm.

(B and C) Histograms showing the number of *PP2A* transcripts **(B)** and the abundance of endogenous H4Ac protein **(C)** per cell within the imaged volume in *Arabidopsis* root cross-sections. The dashed lines indicate median values.

(D) Violin plots show the distribution of *PP2A* mRNA and H4Ac protein levels across cell types from the same *Arabidopsis* root cross-section. Values represent transcript number/protein intensity normalized by cell size. Boxes inside the violin plots show the interquartile range (IQR, 25%–75%), with median values represented by horizontal lines. Whiskers denote ±1.58 × IQR. Statistical analyses were performed using one-way ANOVA followed by Tukey's honestly significant difference (HSD) tests. A p value greater than 0.05 indicates no statistical significance (ns), whereas p values less than 0.05, 0.01, and 0.001 are indicated by *, **, and ***, respectively. Only significant differences are labeled. Experiments were repeated independently four times.

within the same cells and across different cell types (Figure 4D). Importantly, this sequential cryo-smFISH/IF protocol can be extended to crop species, such as barley (Figure 4A). The development of this protocol provides a valuable tool for colocalization studies, especially in species for which transgenesis has not been achieved.

DISCUSSION

In this study, we introduce an optimized smFISH protocol designed specifically for plant tissue cryosections. Our cryo-smFISH method provides an effective approach for precisely quantifying the spatial distribution of RNA with single-cell and single-molecule resolution in plant tissue sections. Compared with paraffin-embedding workflows, cryosectioning can reduce sample-processing time and avoid dehydration/clearing steps. While cryosections can be more sensitive to handling and may require careful optimization to preserve tissue morphology, they generally provide strong preservation of biomolecules, including protein epitopes and nucleic acids (Hira et al., 2019). Relative to our previous smFISH protocol for whole-mount tissue (Zhao et al., 2023), the choice of method will depend on the type of

material and the final objective of the study. While whole-mount smFISH performs well in preserving tissue structure, it may yield inferior signal-to-noise ratios for thicker specimens. Furthermore, if the goal is to combine RNA detection with protein detection through immunofluorescence, cryosectioning offers a distinct advantage, as antibody penetration is notably more efficient in tissue cryosections. The use of thin sections also allows for the use of widefield microscopes, which can be invaluable when confocal microscopes are not readily available. Moreover, widefield systems for cryo-smFISH samples can significantly expedite image acquisition compared with confocal imaging, while still allowing for excellent segmentation results. A structured comparison of cryo-smFISH with whole-mount and other section-based smFISH workflows is provided in Supplemental Table 1.

The complexity of vascular tissue structure and development has historically made high-resolution gene expression profiling challenging. Detailed gene expression profiling in plant vascular tissues has predominantly been explored through scRNA-seq (Wendrich et al., 2020; Chen et al., 2021; Otero et al., 2022; Kułak et al., 2023). Here, we employed cryo-smFISH to physically map and quantify the vascular tissue-specific gene *NRT1.9* within individual cells in a transgene-free manner. Our analysis, combining scRNA-seq with cryo-smFISH, highlights smFISH as a powerful method for validating scRNA-seq data while complementing it with cellular and subcellular spatial information. This further reinforces the importance of expanding the use of smFISH across several plant tissues and preparations, particularly as the demand for scRNA-seq validation methods continues to rise.

Lastly, cryo-smFISH is sensitive enough to detect low-abundance transcripts, such as lncRNAs, thereby expanding our ability to study RNA function at both cellular and subcellular levels. However, several limitations should also be noted. While some tissues, such as barley roots, yield good cryosections relatively easily, others, such as leaves or *Arabidopsis* roots, can be more challenging because tissue integrity is difficult to maintain and may require careful optimization and practice. Detection in highly autofluorescent tissues also remains challenging and requires optimization for each specific tissue type. This limitation is especially evident in adult *Arabidopsis* leaves, where high background fluorescence strongly limits robust RNA detection. Under our conditions, detectable signals were obtained only with Quasar670-labeled probes in the far-red range, where tissue autofluorescence is lower, underscoring the difficulty of applying cryo-smFISH reliably in this tissue. Additional optimizations, such as prolonged clearing treatments, may be necessary for different samples to reduce background autofluorescence and improve signal detection. Additionally, like most smFISH methods, our protocol has limitations in detecting RNAs shorter than 600 nucleotides and in co-detecting multiple RNA species (see Supplemental Table 2 for additional strengths and limitations of cryo-smFISH). Several technical advances could further improve the sensitivity and versatility of plant smFISH. For example, sensitivity could be enhanced through probe-design strategies that increase fluorescence output per transcript, such as increasing the number or density of probes targeting the same RNA (Wang and Guo, 2021). In parallel, other RNA-imaging approaches, including live-cell RNA-labeling systems such as MS2, provide complementary approaches for studying transcriptional dynamics in plants (Tantale et al., 2016; Alamos et al., 2021; Hani et al., 2021).

In summary, our cryo-smFISH method represents a valuable tool for precisely quantifying single RNA molecules in plant tissue sections with high spatial resolution. This innovative approach enables plant biologists to investigate RNA abundance and localization at both cellular and subcellular resolution, thereby greatly expanding the experimental toolkit for spatial studies of gene expression in plants.

METHODS

Plant materials and growth conditions

Arabidopsis thaliana Col-0 and barley (*Hordeum vulgare*) were used in this study. Barley (*Hordeum vulgare*) seeds were a gift from Silvana Moreno (Department of Plant Biology, SLU, Uppsala, Sweden). The pHvpPIN1a:HvPIN1a-mVENUS barley line was a gift from Prof. Rüdiger Simon (Heinrich Heine University, Germany). Seeds were surface-sterilized in 1 ml of 70% ethanol in 2 ml Eppendorf tubes on a rotating wheel for 5–10 min, followed by three washes with 95% ethanol. The ethanol was then removed, and the seeds were allowed to dry in a laminar-flow hood.

After stratification for 2–3 days, seeds were sown on Murashige and Skoog (MS) agar plates. The plates were then transferred to a growth chamber under a 16-h light/8-h dark photoperiod, with temperatures of 22°C during the day and 20°C during the night. *Arabidopsis* root tips were collected 7 days after germination. Barley roots were collected 4 days after germination.

smFISH probe design

Probes targeting the genes of interest were designed using the online Stellaris Probe Designer from Biosearch Technologies: https://www.biosearchtech.com/stellaris-designer. For mRNA detection, the coding sequence of the target gene was entered into the program, which automatically generated a set of probes complementary to the target mRNA.

The sequences of the probes were then subjected to quality control using an automated local blast R script, available on GitHub at: https://github.com/xuezhang911/zhang_et_al_smFISH_cyrosections/tree/main/smFISHprobes. The smFISH probes used in this study and their respective fluorophores are shown in Supplemental Table 3. The probes were diluted in Tris-EDTA buffer to a final stock concentration of 25 μM.

Cryo-smFISH

Sample preparation

Tissues were dissected using a razor blade and fixed for 30 min for roots or 1 h for green tissues in 4% (w/v) paraformaldehyde (PFA) solution in nuclease-free 1× PBS buffer (pH 8.0; Sigma–Aldrich, cat. # AM9624). Following fixation, the tissues were cryoprotected in 34% sucrose in 1× PBS, followed by overnight incubation with mild shaking in a 1:1 mixture of 34% sucrose in 1× PBS and liquid optimal cutting temperature compound (OCT; Leica Biosystems, cat. # 14020108926) at 4°C. Subsequently, the samples were incubated in pre-chilled liquid OCT for 30 min to 1 h with mild shaking before tissue blocks were prepared. Using tweezers, the tissues (roots or leaves) were gently placed into 3D-printed Cryomolds (1 cm × 1 cm × 1 cm) filled with pre-chilled OCT. These OCT-embedded tissue blocks were frozen either by contact with dry ice or by indirect contact with liquid nitrogen. The cryoblocks were then wrapped in foil and stored at −80°C until cryosectioning. Cryosections of 10 μm for *Arabidopsis* and barley roots or 5 μm for barley leaves were cut using a cryostat (Leica CM1850) and mounted onto Polysine adhesion microscope slides (cat. # 48 382-117), followed by air-drying for up to 20 min and post-fixation for 10 min with 4% PFA at room temperature (RT). Subsequently, after rinsing three times with 1× PBS, the slides containing sections were permeabilized sequentially in methanol for 30 min, ethanol for 30 min, and ClearSee solution containing 10% (w/v) xylitol

powder (Sigma–Aldrich, cat. # X3375), 15% (w/v) sodium deoxycholate (Sigma–Aldrich, cat. # D6750), and 25% (w/v) urea (Sigma–Aldrich, cat. # U5378) dissolved in RNase-free distilled water (Qiagen, cat. # 10-977-015). The slides were then stored at 4°C overnight until the subsequent smFISH procedure. To confirm RNA specificity, samples were incubated with RNase A (Sigma–Aldrich, cat. # R4642) for 1 h at 37°C in a humid chamber after the clearing step. Samples were then rinsed in 10 mM HCl for 5 min and washed twice in 2× saline-sodium citrate (SSC) for 5 min each before proceeding with hybridization.

Hybridization

The procedure was performed as previously described, with slight modifications (Duncan et al., 2016; Zhao et al., 2023). Briefly, slides prepared with cryosectioned samples were first rinsed 2–3 times with wash buffer containing 10% (v/v) nuclease-free 20× SSC (Thermo Scientific, cat. # AM9763), 10% (v/v) deionized formamide (Merck, cat. #S4117), and 0.1% (v/v) Triton X-100 (Sigma–Aldrich, cat. # T8787). A total volume of 100 μl of hybridization solution containing 10% (w/v) dextran sulfate (Sigma–Aldrich, cat. # RES2029D), 10% (v/v) deionized formamide, and probes at a final concentration of 250 nM was added to each slide. Plastic coverslips were placed over the samples to prevent buffer evaporation, and the probes were hybridized in a humid chamber at 37°C overnight in the dark.

Post-hybridization and mounting

Plastic coverslips were gently removed, and the hybridization solution containing unbound probes was removed by rinsing the slides twice with wash buffer. Subsequently, the slides were immersed in Coplin jars containing wash buffer for up to 1 h at 37°C. DAPI (1:10,000) or SR2200 dye (1:50,000; Renaissance Chemicals Ltd.; see Musielak et al., 2016 for recipes) prepared in wash buffer was applied for 10–15 min or 5–10 min, respectively. After washing for 5–10 min with 2× SSC buffer, the samples were mounted in freshly prepared GLOX mounting medium (0.4% glucose; 10 nM Tris–HCl, pH 8.0 [Invitrogen, cat. # AM9856]; and 2× SSC; supplemented with 1/100 [v/v] glucose oxidase [Sigma, cat. # G2133-10KU] and 2/100 [v/v] gently vortexed bovine liver catalase [Sigma, cat. # C3155-50MG]) and sealed with nail polish.

A step-by-step protocol for cryo-smFISH can be found in the Supplemental Information (Supplemental Methods 1).

smFISH followed by immuno-fluorescence (IF)

We conducted cryo-smFISH and IF sequentially; the cryo-smFISH protocol for RNA detection was performed first as described above, followed by immunofluorescence after cryo-smFISH image acquisition. After imaging, the coverslips were gently removed, and the samples were rinsed three times with 1× PBS. Samples were then incubated with enzyme mix (2% Driselase [Sigma–Aldrich, cat. # D8037], 1% cellulase [Sigma–Aldrich, cat. # C1184], and 2% macerozyme [Duchefa, cat. # M8002] in 1× PBS) for 15 min in a humid chamber at 37°C. After washing three times with 1× PBS, the samples were incubated with 5% BSA blocking buffer in a humid chamber for 30 min at 37°C. Subsequently, the samples were incubated with an anti-H4Ac antibody (Bio-Rad, AHP418) diluted 1:100 in 5% BSA in a humid chamber overnight at 4°C. The samples were then washed with PBST (0.1% Tween in 1× PBS) by incubation in Coplin jars at 37°C for 30 min. The samples were incubated with a secondary antibody (Agrisera, AS09633) diluted 1:200 in 5% BSA in a humid chamber at 37°C for 2 h. Excess antibody was removed, and the samples were washed with PBST in Coplin jars at 37°C in the dark for 30 min. The samples were then rinsed with 1× PBS and mounted in a drop of Vectashield mounting medium (Bionordika, cat. # VEH-1000). A step-by-step protocol for cryo-smFISH/IF can be found in the Supplemental Information (Supplemental Methods 1).

Image acquisition

A Zeiss LSM800 inverted confocal microscope controlled by ZEN Black software was used for imaging with a 63× water-immersion objective (1.20 NA). We acquired z-stacks with 0.2-μm spacing. The following channel settings were used: DAPI/SR2200, 353 nm excitation and 420–470 nm emission; Quasar570, 548 nm excitation and 570–640 nm emission; Quasar670, 650 nm excitation and 665–715 nm emission; and H4Ac, 488 nm excitation and 500–550 nm emission.

For widefield microscopy, we acquired 10–40 z-stack images with a cooled quad-port charge-coupled device (CCD) ZEISS Axiocam 503 mono camera using a 63× water-immersion objective (1.20 NA). The following channel settings were used: Quasar570, 533–558 nm excitation and 561 nm emission; Quasar670, 625–655 nm excitation and 673 nm emission; and DAPI/SR2200, 335–383 nm excitation and 465 nm emission.

Image analysis

To quantify transcript number and protein levels per cell within the cut section in an unbiased manner, we adapted the automated computational workflow used by Zhao et al. (2023). Our workflow integrates functions from four freely available software programs: Fiji (Schindelin et al., 2012), Cellpose (Stringer et al., 2021), FISH-quant (Mueller et al., 2013; Imbert et al., 2022), and CellProfiler (Carpenter et al., 2006; Stirling et al., 2021), together with a custom Python script. Analyses were performed using separate TIF images for each channel: SR2200, DAPI, smFISH, or IF. Shifts and misalignments between channels and images acquired from the same section were corrected using the BigWarp tool in Fiji (Bogovic et al., 2016). Maximum Z-projection or single-plane confocal or widefield images were used for analysis. A step-by-step image analysis protocol can be found in the supplemental information (Supplemental Methods 2).

Cell and nuclear segmentation

Image segmentation was performed using Cellpose-based algorithms (Stringer et al., 2021).

For cell segmentation, SR2200-channel images were used as input, and the ‘‘cyto’’ algorithm was selected as the pre-trained model for annotating individual cells within the tissue. To achieve accurate cell segmentation, semi-supervised training using SR2200-channel images was implemented. Following the annotation step, manual corrections and successive training cycles were applied until cell borders were precisely outlined. New models specifically designed to segment cells in *Arabidopsis* or barley tissue sections were generated and used to create cell masks for the subsequent transcript quantification step.

For nucleocytoplasmic-level analyses, we first trained a new segmentation model from the pre-loaded ‘‘nuclei’’ algorithm or from the cell segmentation model developed earlier from SR2200 images, using semi-supervised training as outlined above. As a result, we established two models specifically designed to segment cells with well-defined cell borders and nuclei within *Arabidopsis* or barley tissue sections. These two models were subsequently used to create cell and nucleus masks for the subsequent transcript quantification step.

The custom segmentation models are available on GitHub at: https://github.com/xuezhang911/zhang_et_al_smFISH_cyrosections/tree/main/smFISHprobes.

Image filtering and dot quantification

We employed the MATLAB version of FISH-quant v3 to quantify RNA dots in cryo-smFISH images (Mueller et al., 2013; Imbert et al., 2022). The software and its accompanying manual can be accessed on Bitbucket, as provided by Florian Mueller: https://bitbucket.org/muellerflorian/fish_quant/src/master/.

The ‘‘Cell Segmentation’’ tool was first used to generate text files containing FISH-quant-recognized nuclei or cell outline coordinates from the Cellpose-generated masks. After the outline text file for a given image

was imported, the loaded cryo-smFISH image was filtered by applying a dual-Gaussian filter. For widefield cryo-smFISH images in Supplemental Figure 9, a preprocessing deconvolution step was performed using the open-source software DeconvolutionLab2 (https://bigwww.epfl.ch/deconvolution/deconvolutionlab2/) (Sage et al., 2017). The filtered image was then smoothed with a Gaussian kernel. The Gaussian kernel ratio required adjustment for each gene and different tissue contexts.

Subsequently, we conducted dot pre-detection in one selected filtered image. This was performed in the filtered image by defining threshold intensity and quality scores that enabled accurate detection of fluorescent foci. Next, the detected foci were fitted using Gaussian fluorescence fitting based on the point-spread function (PSF). The settings were then applied to run the analysis in batch mode using the batch analysis tool.

False positives were removed by thresholding the sigma–XY, amplitude, and pixel-intensity parameters, following the developer's advice. The results were exported as tabulated files, indicating the number of transcripts per segmented cell or nucleus within each section for each object identifier.

Image visualization

Visualization Cells and nuclei: Cell and nuclear masks were created using Cellpose and imported into CellProfiler to generate cellular and nuclear outlines that trace the contours of individual cells and their nuclei. These outline images were then imported into Adobe Illustrator. The high-resolution images, while preserving fine detailed tissue structures, were finalized through image vectorization and manual adjustment of the outline thickness for phloem sieve elements from *Arabidopsis* and barley roots.

Cryo-smFISH dot visualization: An image displaying cryo-smFISH dots was generated either from the filtered image during the transcript quantification step or from manually plotted dots based on the coordinates provided by the final quantification output. This image was then overlaid with the original cryo-smFISH RNA channel to enhance visualization.

Protein quantification

The immunofluorescence images were analyzed using Cellprofiler (Carpenter et al., 2006; Stirling et al., 2021). Cellpose-generated masks were imported and used to identify each nucleus or cell as an independent object. From the IF images, we computed both the total intensity and the mean intensity for each object, allowing us to determine protein levels on a per-nucleus or per-cell basis. The results were exported as tabulated files.

Assignment of RNA or protein signals to individual cells within cell types

To assign gene transcript numbers to specific cell types, we first obtained the RNA mask and the cell/nuclei mask. For the RNA mask, we visualized RNA molecules by displaying their XY coordinates on a blank image. These coordinates, representing the centers of the dots, were obtained during the transcript detection step. For the cell/nuclei mask, we used Cellpose-generated masks. Subsequently, we imported both the RNA mask and the cell/nuclei mask into CellProfiler, and the number of RNA molecules per individual cell was counted and displayed on the cell/nuclei masks. Similarly, after we measured protein intensity with CellProfiler, the intensity within individual cells was quantified and displayed on cell masks. Consequently, images with well-defined tissue structures, cell/nuclei borders, and RNA masks as graphical representations of the single-cell results were produced.

scRNA-seq analysis

The normalized count matrix in HDF5 (.h5) format was downloaded from the Gene Expression Omnibus (GEO: GSE141730_aggregated_filtered_gene_bc_matrices.h5). Feature selection, dimensionality reduction, and clustering were performed as described in the original study (Wendrich et al., 2020). Cell types were manually annotated using the marker genes reported in that study. For downstream analyses, the full Seurat object was subsetted to include only epidermis, cortex, endodermis, pericycle, phloem, procambium, and xylem cells.

Bulk RNA-seq analysis

The WT FASTQ files were downloaded from the NCBI GEO study GSE96945. After rRNA removal with SortMeRNA (Kopylova et al., 2012), adapters were trimmed with Trimmomatic (Bolger et al., 2014). The raw gene-count matrix was obtained using the pseudoalignment software Kallisto (Bray et al., 2016). RNA-seq reads were normalized as transcripts per million (TPM).

DATA AND CODE AVAILABILITY

The supplemental information and all data underlying the graphs and heatmaps presented are available at https://github.com/xuezhang911/zhang_et_al_smFISH_cyrosections.

All custom code, including R/Python scripts and Cellpose segmentation models, is available at https://github.com/xuezhang911/zhang_et_al_smFISH_cyrosections.

FUNDING

This work was supported by Vetenskapsrådet (2023-03895), the Novo Nordisk Foundation (NFF24OC0093553 and NNF25OC0100533), and the Carl Tryggers Stiftelse (CTS 18- 325).

ACKNOWLEDGMENTS

We thank A. Menkis for initial technical support with cryostat operation and Alexandre Berr for scientific feedback. We also thank members of the Sicard and Rosa groups for discussions and comments on the article. No conflict of interest declared.

AUTHOR CONTRIBUTIONS

X.Z., A.S., and S.R. designed the research. X.Z. performed cryo-smFISH experiments. K.K. performed IF experiments. X.Z. analyzed the scRNA-seq and bulk RNA-seq data. A.F. and X.Z. performed image analysis. X.Z. and A.F. prepared the figures. S.D. contributed to method optimization. M.L. contributed to cryo-smFISH experiments during the revision. X.Z., A.F., and S.R. wrote the manuscript. All authors commented on and approved the final manuscript.

SUPPLEMENTAL INFORMATION

Supplemental information is available at *Plant Communications Online*.

Received: December 16, 2025
Revised: May 11, 2026
Accepted: June 4, 2026
Published: June 8, 2026

REFERENCES

Alamos, S., Reimer, A., Niyogi, K.K., and Garcia, H.G. (2021). Quantitative imaging of RNA polymerase II activity in plants reveals the single-cell basis of tissue-wide transcriptional dynamics. Nat. Plants **7**:1037–1049.

Anjam, M.S., Ludwig, Y., Hochholdinger, F., Miyaura, C., Inada, M., Siddique, S., and Grundler, F.M.W. (2016). An improved procedure for isolation of high-quality RNA from nematode-infected Arabidopsis roots through laser capture microdissection. Plant Methods **12**:25.

Bauman, J.G.J., Wiegant, J., Borst, P., and van Duijn, P. (1980). A new method for fluorescence microscopical localization of specific DNA sequences by in situ hybridization of fluorochrome-labelled RNA. Exp. Cell Res. **128**:485–490.

Bawa, G., Liu, Z., Yu, X., Qin, A., and Sun, X. (2022). Single-Cell RNA Sequencing for Plant Research: Insights and Possible Benefits. Int. J. Mol. Sci. **23**:4497.

Bogovic, J.A., Hanslovsky, P., Wong, A., and Saalfeld, S. (2016). Robust registration of calcium images by learned contrast synthesis. In 2016 IEEE 13th International Symposium on Biomedical Imaging (ISBI) (IEEE), pp. 1123–1126.

Bolger, A.M., Lohse, M., and Usadel, B. (2014). Trimmomatic: a flexible trimmer for Illumina sequence data. Bioinformatics **30**:2114–2120.

Bray, N.L., Pimentel, H., Melsted, P., and Pachter, L. (2016). Near-optimal probabilistic RNA-seq quantification. Nat. Biotechnol. **34**:525–527.

Brigati, D.J., Myerson, D., Leary, J.J., Spalholz, B., Travis, S.Z., Fong, C.K., Hsiung, G.D., and Ward, D.C. (1983). Detection of viral genomes in cultured cells and paraffin-embedded tissue sections using biotin-labeled hybridization probes. Virology **126**:32–50.

Cabili, M.N., Dunagin, M.C., McClanahan, P.D., Biaesch, A., Padovan-Merhar, O., Regev, A., Rinn, J.L., and Raj, A. (2015). Localization and abundance analysis of human lncRNAs at single-cell and single-molecule resolution. Genome Biol. **16**:20.

Carpenter, A.E., Jones, T.R., Lamprecht, M.R., Clarke, C., Kang, I.H., Friman, O., Guertin, D.A., Chang, J.H., Lindquist, R.A., Moffat, J., et al. (2006). CellProfiler: image analysis software for identifying and quantifying cell phenotypes. Genome Biol. **7**:R100.

Chen, H., Yin, X., Guo, L., Yao, J., Ding, Y., Xu, X., Liu, L., Zhu, Q.-H., Chu, Q., and Fan, L. (2021). PlantscRNAdb: A database for plant single-cell RNA analysis. Mol. Plant **14**:855–857.

Choe, J.e., Kim, B., Yoon, E.K., Jang, S., Kim, G., Dhar, S., Lee, S.A., and Lim, J. (2017). Characterization of the GRAS transcription factor SCARECROW-LIKE 28's role in Arabidopsis root growth. J. Plant Biol. **60**:462–471.

Cox, K.H., DeLeon, D.V., Angerer, L.M., and Angerer, R.C. (1984). Detection of mRNAs in sea urchin embryos by in situ hybridization using asymmetric RNA probes. Dev. Biol. **101**:485–502.

Denyer, T., Ma, X., Klesen, S., Scacchi, E., Nieselt, K., and Timmermans, M.C.P. (2019). Spatiotemporal Developmental Trajectories in the Arabidopsis Root Revealed Using High-Throughput Single-Cell RNA Sequencing. Dev. Cell **48**:840–852.e5.

Djebali, S., Davis, C.A., Merkel, A., Dobin, A., Lassmann, T., Mortazavi, A., Tanzer, A., Lagarde, J., Lin, W., Schlesinger, F., et al. (2012). Landscape of transcription in human cells. Nature **489**:101–108.

Duncan, S., Johansson, H.E., and Ding, Y. (2023). Reference genes for quantitative Arabidopsis single molecule RNA fluorescence *in situ* hybridization. P. Manavella, ed. **74**:2405–2415.

Duncan, S., Olsson, T.S.G., Hartley, M., Dean, C., and Rosa, S. (2016). A method for detecting single mRNA molecules in Arabidopsis thaliana. Plant Methods **12**:13.

Duncan, S., Olsson, T.S.G., Hartley, M., Dean, C., and Rosa, S. (2017). Single Molecule RNA FISH in Arabidopsis Root Cells. Bio. Protoc. **7**:e2240.

Femino, A.M., Fay, F.S., Fogarty, K., and Singer, R.H. (1998). Visualization of Single RNA Transcripts in Situ. Science **280**:585–590.

Hani, S., Cuyas, L., David, P., Secco, D., Whelan, J., Thibaud, M.-C., Merret, R., Mueller, F., Pochon, N., Javot, H., et al. (2021). Live single-cell transcriptional dynamics via RNA labelling during the phosphate response in plants. Nat. Plants **7**:1050–1064.

Harrison, P.R., Conkie, D., Paul, J., and Jones, K. (1973). Localisation of cellular globin messenger RNA by *in situ* hybridisation to complementary DNA. FEBS (Fed. Eur. Biochem. Soc.) Lett. **32**:109–112.

He, Z., Lan, Y., Zhou, X., Yu, B., Zhu, T., Yang, F., Fu, L.-Y., Chao, H., Wang, J., Feng, R.-X., et al. (2024). Single-cell transcriptome analysis dissects lncRNA-associated gene networks in Arabidopsis. Plant Commun. **5**:100717.

Heiles, H.B., Genersch, E., Kessler, C., Neumann, R., and Eggers, H.J. (1988). In situ hybridization with digoxigenin-labeled DNA of human papillomaviruses (HPV 16/18) in HeLa and SiHa cells. Biotechniques **6**:978–981.

Hira, V.V.V., De Jong, A.L., Ferro, K., Khurshed, M., Molenaar, R.J., and Van Noorden, C.J.F. (2019). Comparison of different methodologies and cryostat versus paraffin sections for chromogenic immunohistochemistry. Acta Histochem. **121**:125–134.

Huang, K., Batish, M., Teng, C., Harkess, A., Meyers, B.C., and Caplan, J.L. (2020). Quantitative Fluorescence In Situ Hybridization Detection of Plant mRNAs with Single-Molecule Resolution. In Methods in Molecular Biology. RNA Tagging, M. Heinlein, ed. (Springer US), pp. 23–33.

Huang, T., Guillotin, B., Rahni, R., Birnbaum, K.D., and Wagner, D. (2023). A rapid and sensitive, multiplex, whole mount RNA fluorescence in situ hybridization and immunohistochemistry protocol. Plant Methods **19**:131.

Hutchison, N.J., Langer-Safer, P.R., Ward, D.C., and Hamkalo, B.A. (1982). In situ hybridization at the electron microscope level: hybrid detection by autoradiography and colloidal gold. J. Cell Biol. **95**:609–618.

Imbert, A., Ouyang, W., Safieddine, A., Coleno, E., Zimmer, C., Bertrand, E., Walter, T., and Mueller, F. (2022). FISH-quant v2: a scalable and modular tool for smFISH image analysis. RNA **28**:786–795.

Jha, U.C., Nayyar, H., Jha, R., Khurshid, M., Zhou, M., Mantri, N., and Siddique, K.H.M. (2020). Long non-coding RNAs: emerging players regulating plant abiotic stress response and adaptation. BMC Plant Biol. **20**:466.

Kim, J.Y., Lee, J., Kang, M.H., Trang, T.T.M., Lee, J., Lee, H., Jeong, H., and Lim, P.O. (2022). Dynamic landscape of long noncoding RNAs during leaf aging in Arabidopsis. Front. Plant Sci. **13**:1068163.

Kopylova, E., Noé, L., and Touzet, H. (2012). SortMeRNA: fast and accurate filtering of ribosomal RNAs in metatranscriptomic data. Bioinformatics **28**:3211–3217.

Kułak, K., Wojciechowska, N., Samelak-Czajka, A., Jackowiak, P., and Bagniewska-Zadworna, A. (2023). How to explore what is hidden? A review of techniques for vascular tissue expression profile analysis. Plant Methods **19**:129.

Lucero, L., Ferrero, L., Fonouni-Farde, C., and Ariel, F. (2020). Functional classification of plant long noncoding RNAs: a transcript is known by the company it keeps. New Phytol. **229**:1251–1260.

Lyubimova, A., Itzkovitz, S., Junker, J.P., Fan, Z.P., Wu, X., and van Oudenaarden, A. (2013). Single-molecule mRNA detection and counting in mammalian tissue. Nat. Protoc. **8**:1743–1758.

Mueller, F., Senecal, A., Tantale, K., Marie-Nelly, H., Ly, N., Collin, O., Basyuk, E., Bertrand, E., Darzacq, X., and Zimmer, C. (2013). FISH-quant: automatic counting of transcripts in 3D FISH images. Nat. Methods **10**:277–278.

Musielak, T., Bürgel, P., Kolb, M., and Bayer, M. (2016). Use of SCRI Renaissance 2200 (SR2200) as a Versatile Dye for Imaging of Developing Embryos, Whole Ovules, Pollen Tubes and Roots. Bio-protocol **6**:e1935.

Nobori, T., Oliva, M., Lister, R., and Ecker, J.R. (2023). Multiplexed single-cell 3D spatial gene expression analysis in plant tissue using PHYTOMap. Nat. Plants **9**:1026–1033.

Otero, S., Gildea, I., Roszak, P., Lu, Y., Di Vittori, V., Bourdon, M., Kalmbach, L., Blob, B., Heo, J.O., Peruzzo, F., et al. (2022). A root

phloem pole cell atlas reveals common transcriptional states in protophloem-adjacent cells. Nat. Plants **8**:954–970.

Panoskaltsis-Mortari, A., and Bucy, R.P. (1995). In situ hybridization with digoxigenin-labeled RNA probes: facts and artifacts. Biotechniques **18**:300–307.

Pardue, M.L., and Gall, J.G. (1969). molecular hybridization of radioactive DNA to the dna of cytological preparations. Proc. Natl. Acad. Sci. USA **64**:600–604.

Raj, A., Van Den Bogaard, P., Rifkin, S.A., Van Oudenaarden, A., and Tyagi, S. (2008). Imaging individual mRNA molecules using multiple singly labeled probes. Nat. Methods **5**:877–879.

Rosa, S., Duncan, S., and Dean, C. (2016). Mutually exclusive sense–antisense transcription at FLC facilitates environmentally induced gene repression. Nat. Commun. **7**:13031.

Sage, D., Donati, L., Soulez, F., Fortun, D., Schmit, G., Seitz, A., Guiet, R., Vonesch, C., and Unser, M. (2017). DeconvolutionLab2: An open-source software for deconvolution microscopy. Methods **115**:28–41.

Schindelin, J., Arganda-Carreras, I., Frise, E., Kaynig, V., Longair, M., Pietzsch, T., Preibisch, S., Rueden, C., Saalfeld, S., Schmid, B., et al. (2012). Fiji: an open-source platform for biological-image analysis. Nat. Methods **9**:676–682.

Shahan, R., Hsu, C.-W., Nolan, T.M., Cole, B.J., Taylor, I.W., Greenstreet, L., Zhang, S., Afanassiev, A., Vlot, A.H.C., Schiebinger, G., et al. (2022). A single-cell Arabidopsis root atlas reveals developmental trajectories in wild-type and cell identity mutants. Dev. Cell **57**:543–560.e9.

Shaw, R., Tian, X., and Xu, J. (2021). Single-Cell Transcriptome Analysis in Plants: Advances and Challenges. Mol. Plant **14**:115–126.

Singer, R.H., and Ward, D.C. (1982). Actin gene expression visualized in chicken muscle tissue culture by using in situ hybridization with a biotinated nucleotide analog. Proc. Natl. Acad. Sci. USA **79**:7331–7335.

Skinner, S.O., Sepúlveda, L.A., Xu, H., and Golding, I. (2013). Measuring mRNA copy number in individual Escherichia coli cells using single-molecule fluorescent in situ hybridization. Nat. Protoc. **8**:1100–1113.

Solanki, S., Ameen, G., Zhao, J., Flaten, J., Borowicz, P., and Brueggeman, R.S. (2020). Visualization of spatial gene expression in plants by modified RNAscope fluorescent in situ hybridization. Plant Methods **16**:71.

Stapel, L.C., Broaddus, C., and Vastenhouw, N.L. (2018). Detection and Automated Analysis of Single Transcripts at Subcellular Resolution in Zebrafish Embryos. In Methods in Molecular Biology. RNA Detection, I. Gaspar, ed. (New York, NY: Springer), pp. 143–162.

Statello, L., Guo, C.-J., Chen, L.-L., and Huarte, M. (2021). Gene regulation by long non-coding RNAs and its biological functions. Nat. Rev. Mol. Cell Biol. **22**:96–118.

Stirling, D.R., Swain-Bowden, M.J., Lucas, A.M., Carpenter, A.E., Cimini, B.A., and Goodman, A. (2021). CellProfiler 4: improvements in speed, utility and usability. BMC Bioinf. **22**:433.

Stringer, C., Wang, T., Michaelos, M., and Pachitariu, M. (2021). Cellpose: a generalist algorithm for cellular segmentation. Nat. Methods **18**:100–106.

Tantale, K., Mueller, F., Kozulic-Pirher, A., Lesne, A., Victor, J.-M., Robert, M.-C., Capozi, S., Chouaib, R., Bäcker, V., Mateos-Langerak, J., et al. (2016). A single-molecule view of transcription reveals convoys of RNA polymerases and multi-scale bursting. Nat. Commun. **7**:12248.

Wang, H., Chung, P.J., Liu, J., Jang, I.-C., Kean, M.J., Xu, J., and Chua, N.-H. (2014). Genome-wide identification of long noncoding natural antisense transcripts and their responses to light in Arabidopsis. Genome Res. **24**:444–453.

Wang, Y.-S., and Guo, J. (2021). Multiplexed Single-Cell in situ RNA Profiling. Front. Mol. Biosci. **8**:775410.

Wang, Y.-Y., and Tsay, Y.-F. (2011). *Arabidopsis* Nitrate Transporter NRT1.9 Is Important in Phloem Nitrate Transport. Plant Cell **23**:1945–1957.

Wendrich, J.R., Yang, B., Vandamme, N., Verstaen, K., Smet, W., Van De Velde, C., Minne, M., Wybouw, B., Mor, E., Arents, H.E., et al. (2020). Vascular transcription factors guide plant epidermal responses to limiting phosphate conditions. Science **370**:eaay4970.

Yang, W., Schuster, C., Prunet, N., Dong, Q., Landrein, B., Wightman, R., and Meyerowitz, E.M. (2020). Visualization of Protein Coding, Long Noncoding, and Nuclear RNAs by Fluorescence in Situ Hybridization in Sections of Shoot Apical Meristems and Developing Flowers. Plant Physiol. **182**:147–158.

Zhang, T.-Q., Xu, Z.-G., Shang, G.-D., and Wang, J.-W. (2019). A Single-Cell RNA Sequencing Profiles the Developmental Landscape of Arabidopsis Root. Mol. Plant **12**:648–660.

Zhao, L., Fonseca, A., Meschichi, A., Sicard, A., and Rosa, S. (2023). Whole-mount smFISH allows combining RNA and protein quantification at cellular and subcellular resolution. Nat. Plants **9**:1094–1102.

Zhao, X., Li, J., Lian, B., Gu, H., Li, Y., and Qi, Y. (2018). Global identification of Arabidopsis lncRNAs reveals the regulation of MAF4 by a natural antisense RNA. Nat. Commun. **9**:5056.

Plant Communications, Volume 7

Supplemental information

Quantitative RNA spatial profiling using single-molecule RNA FISH on plant tissue cryosections

Xue Zhang, Alejandro Fonseca, Konstantin Kutashev, Martina Leso, Adrien Sicard, Susan Duncan, and Stefanie Rosa

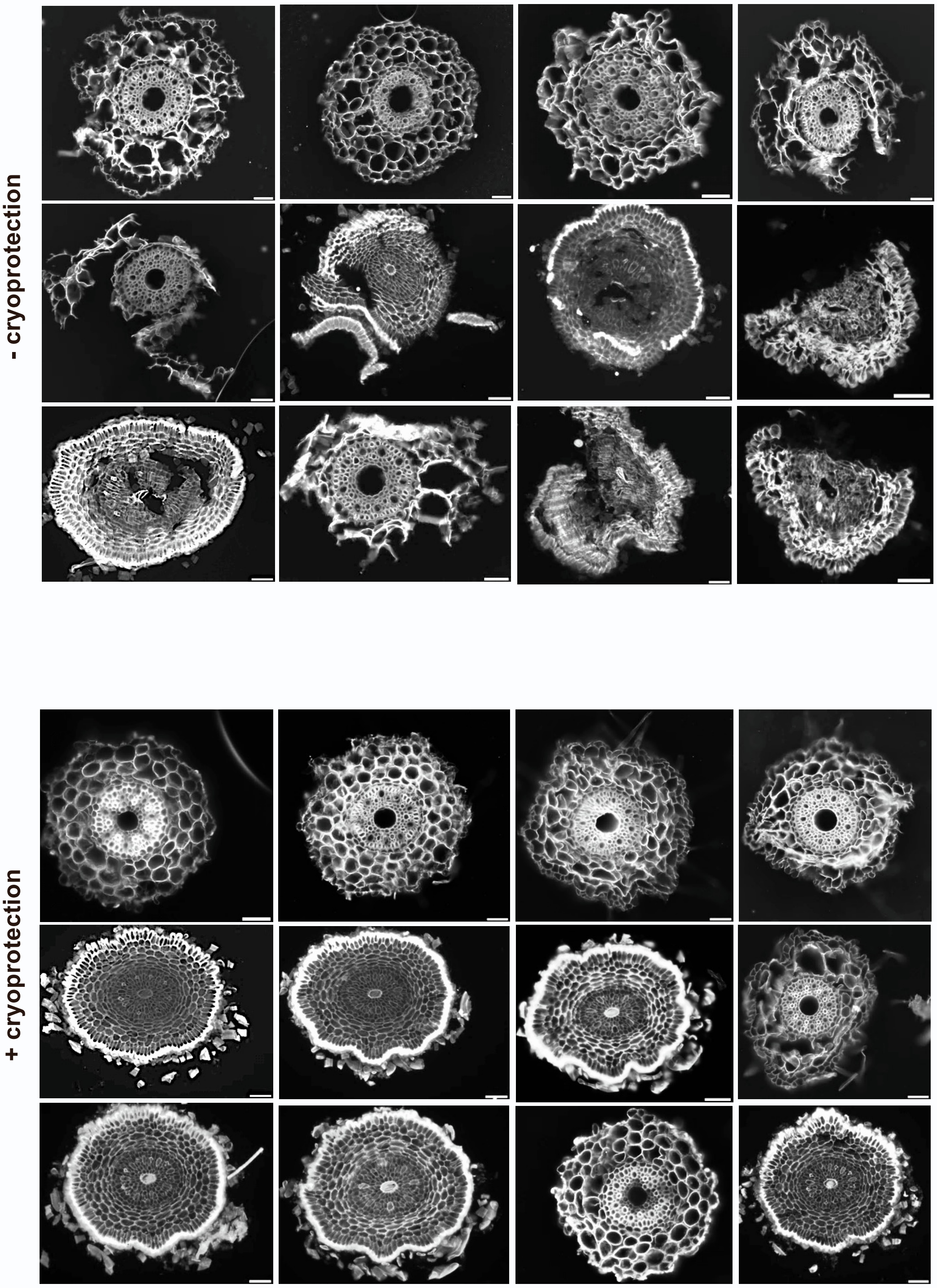

Figure S1. Integrity of barley root sections with and without cryoprotection. Representative widefield microscopy images of barley root cryosections with and without employment of a cryoprotection step. Root sections were stained with SR2200 dye. Scale bars, 10µm.

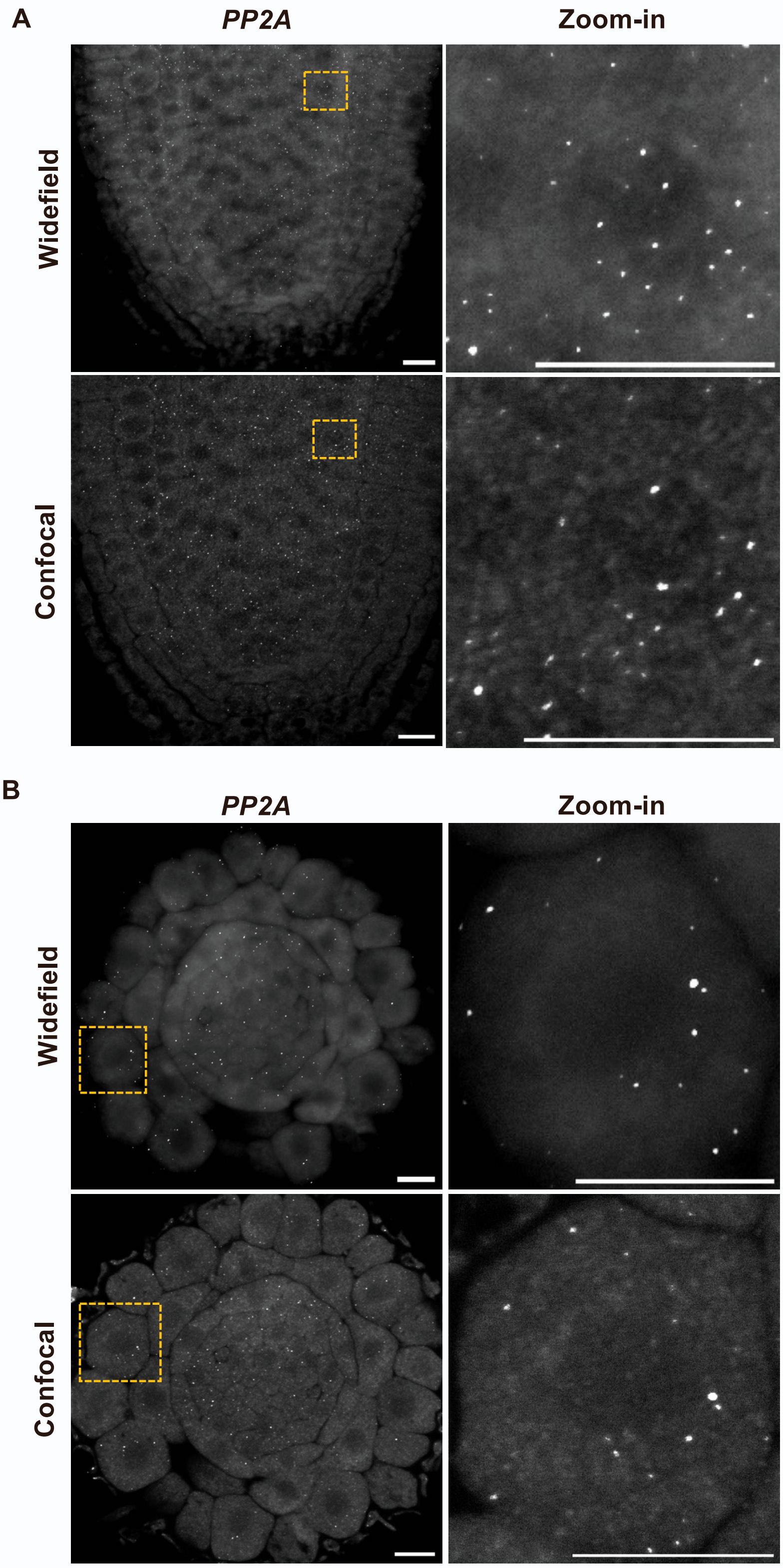

Figure S2. smFISH on *Arabidopsis* root longitudinal and cross-cryosections.
Detection of *PP2A* mRNA using smFISH on *Arabidopsis* root longitudinal (A) and cross-sections (B) using widefield and confocal (single- plane) microscopy. Zoom-in images from regions highlighted in yellow on the left panel. Discrete white dots above the diffuse background correspond to individual RNA molecules. Scale bars, 10µm.

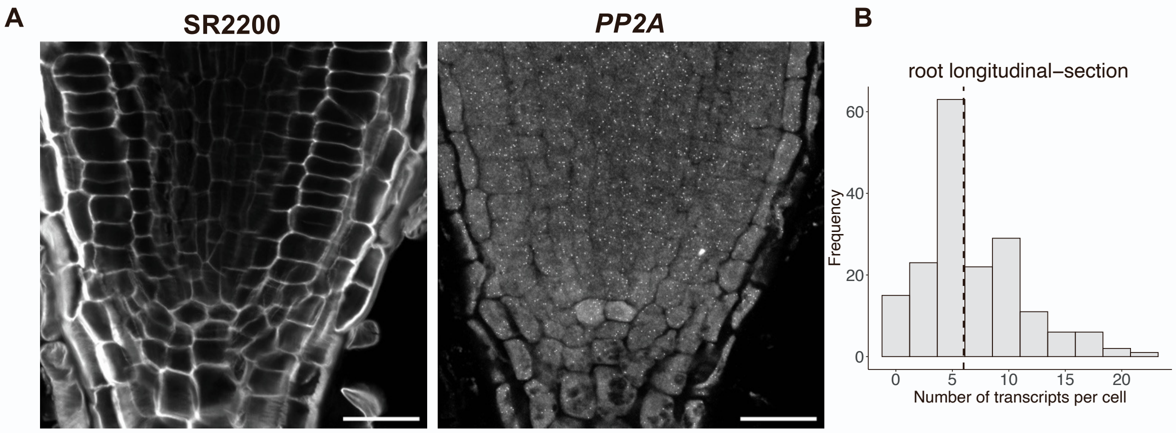

Figure S3. *PP2A* mRNA detection in *Arabidopsis* longitudinal root cryosections. (A) Representative single-plane confocal images of *PP2A* mRNA detected by cryo-smFISH on *Arabidopsis* root longitudinal-section. Left panel: an overview of the tissue using celll wall SR2200 dye; Right panel: *PP2A* cryo-smFISH signals (white dots) corresponding to individual mRNA molecules. Scale bars, 10µm. (C) Histogram showing the distribution of the number of transcripts per cell within the imaged volume detected on root longitudinal-section. The dashed lines mark the median value.

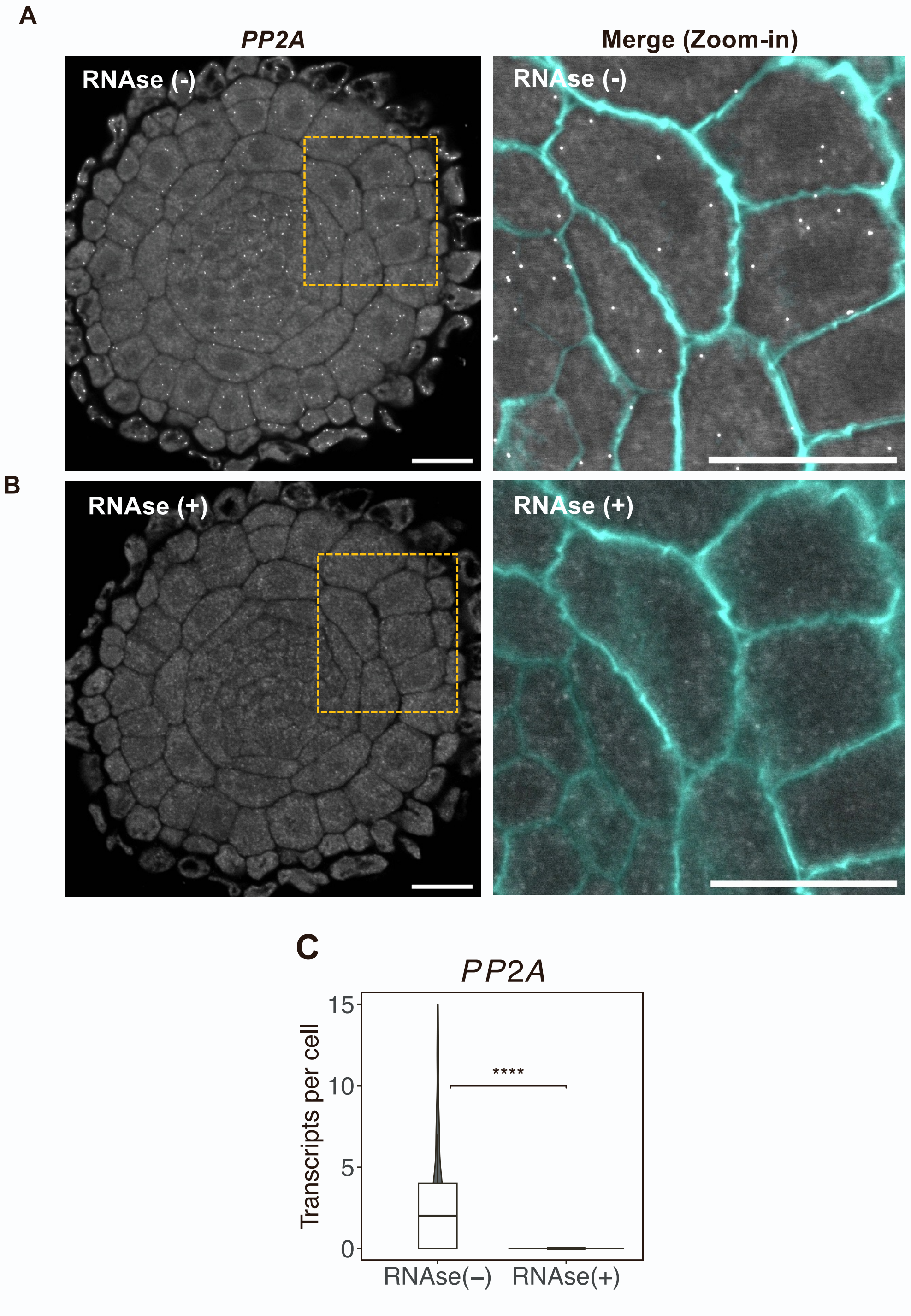

Figure S4. *PP2A* mRNA detection and quantification using cryo-smFISH on *Arabidopsis* root cross-cryosections. (A-B) Representative single-plane confocal images of cryo-smFISH using probes targeting *PP2A* mRNA in *Arabidopsis* root cross-cryosections before (A) and after RNAse treatment (B)**.** Zoom-in images (right panels) of regions highlighted in yellow on the left panel. Discrete white dots correspond to individual RNA molecules. Cell walls stained with SR2200 dye (cyan). Scale bars, 15µm. (C) Violin plot showing the number of transcripts per cell within the imaged volume detected before RNAse (-) and after RNAse (+) treatment. Boxes inside indicate the interquartile range (IQR 25-75%), depicting the median values as a horizontal line. Whiskers represent the ±1.58xIQR value. A two-sided t-test was performed to compare both conditions. p-value less than 0.0001 was denoted by ****.

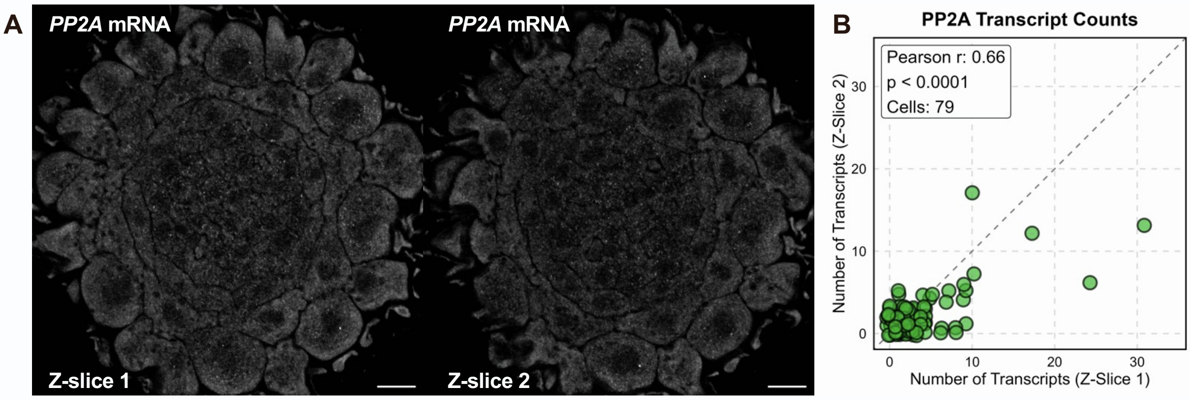

Figure S5. Correlation of *PP2A* transcripts counts across different optical sections. (A) Single-plane confocal images from cryo-smFISH and **(B)** Scatter plot comparing the detected number of *PP2A* transcripts within identical cells across two distinct Z-slice (separated by a 1 µm distance) of a single Arabidopsis root cross-section (each data point represents one cell). The dashed gray line represents a theoretical 1:1 perfect correlation. The Pearson correlation coefficient (r), its associated p-value, and the total number of analyzed cells are indicated in the top left corner of the plot.

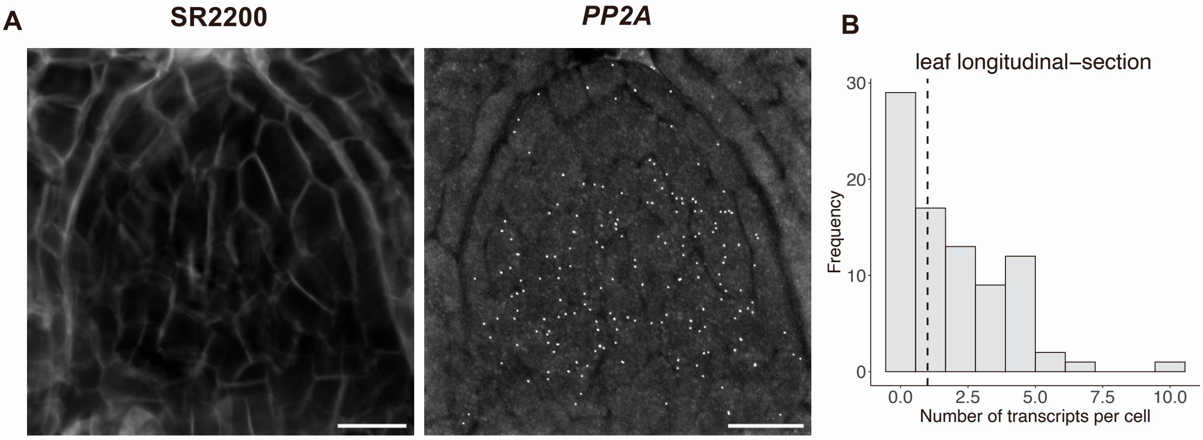

Figure S6. *PP2A* mRNA detection in *Arabidopsis* young leaf cryosections. (A) Representative single-plane confocal images of *PP2A* mRNA detected by cryo-smFISH on *Arabidopsis* young leaf section. Left panel: an overview of the tissue using celll wall SR2200 dye; Right panel: *PP2A* cryo-smFISH signals (white dots) corresponding to individual mRNA molecules. Scale bars, 10µm. (C) Histogram showing the distribution of the number of transcripts per cell within the imaged volume detected on the section. The dashed lines mark the median value.

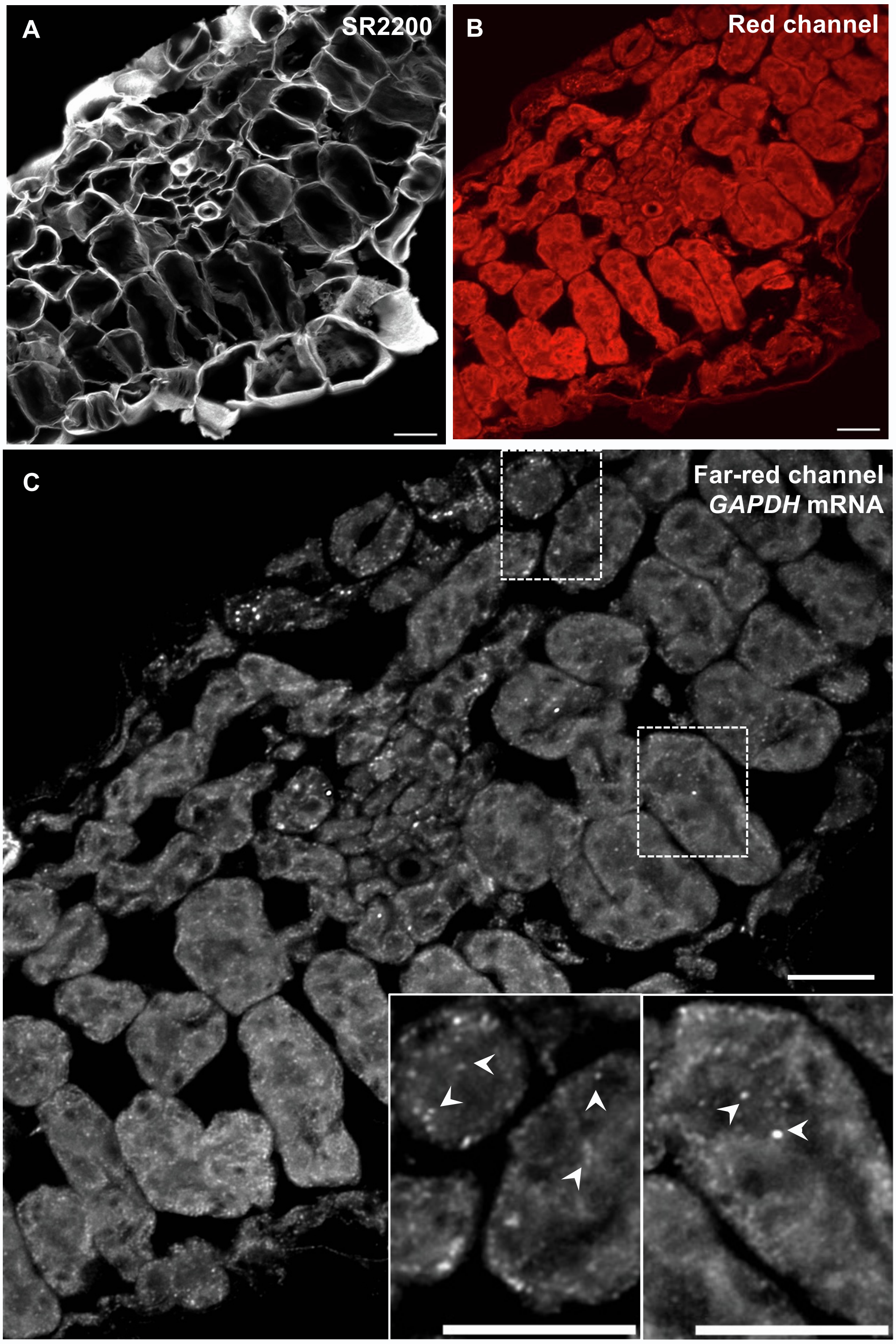

Figure S7. *GAPDH* mRNA detection in *Arabidopsis* cross sections from adult leaves. (A) Overview of the tissue with cell wall staining using SR2200 dye. (B) Autofluorescence in the red channel. (C) Detection of *GAPDH* using smFISH probes labelled with far-red dye (Quasar670). Images represent a maximum intensity projection of five Z-stacks with a Z-step of 0.5 µm. Raw images were deconvolved using the NIS-Elements 3D deconvolution algorithm prior to generating the projection. Arrows indicate mRNA detection. Inset: magnified view of the region highlighted. Scale bars, 10 µm.

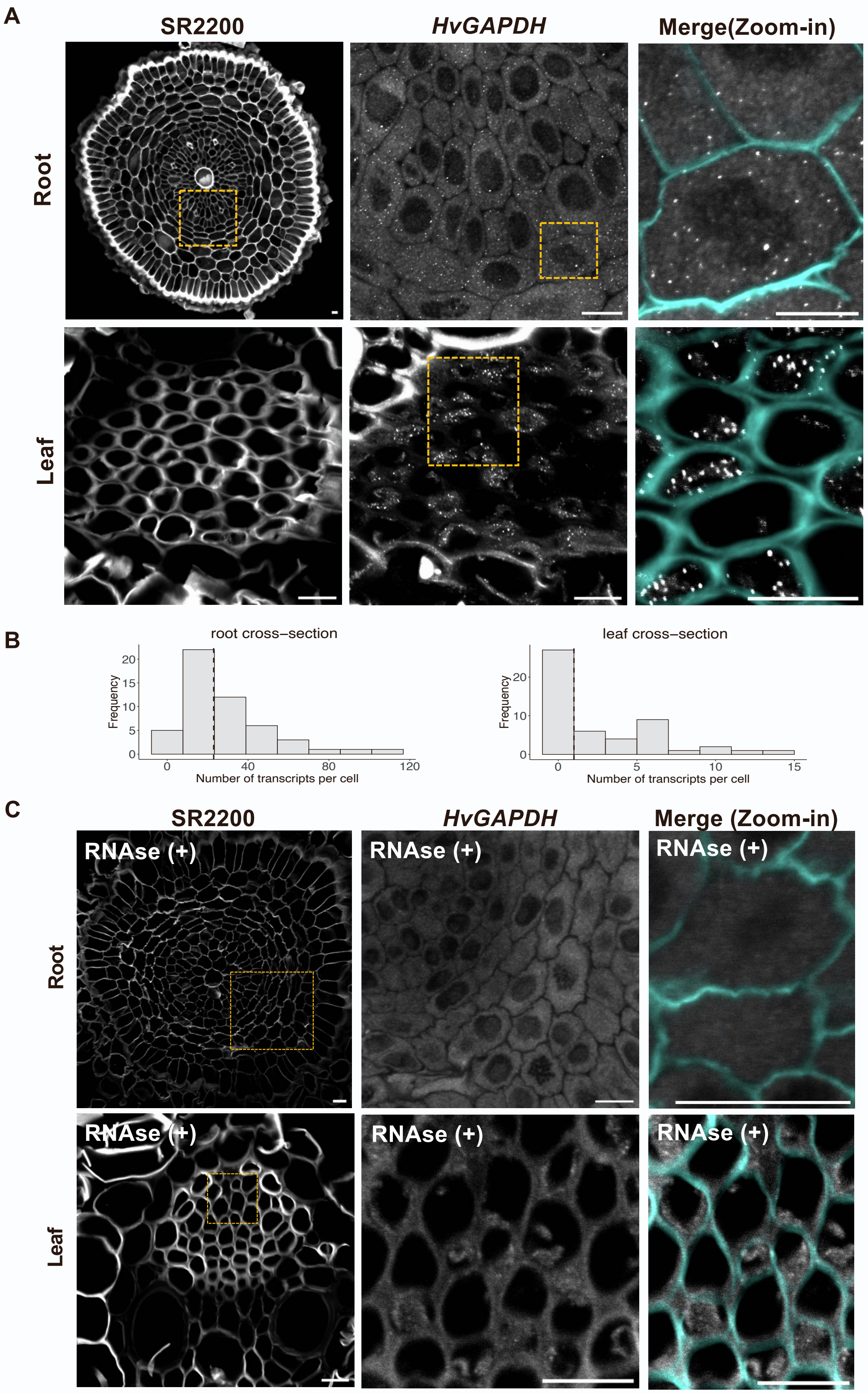

Figure S8. Detecting *HvGAPDH* mRNA in barley roots and leaves using cryo-smFISH.
(A, C) Representative single-plane confocal images of cryo-smFISH using probes targeting *HvGAPDH* mRNA in barley root and leaf cross-sections with (C) and without (A) RNAse treatment**.** Left panels: cell wall staining with SR2200 dye; Middle panels: cryo-smFISH channel; Right panels: zoomed-in images of regions highlighted in yellow in the left panels showing cryo-smFISH channel (white) and cell wall staining (cyan). Scale bars, 10µm. (B) Histograms showing the distribution of the number of transcripts per cell within the imaged volume detected in the root (left) and leaf cross-sections (right). Dashed lines indicate the median value. Experiments were repeated independently 2 times.

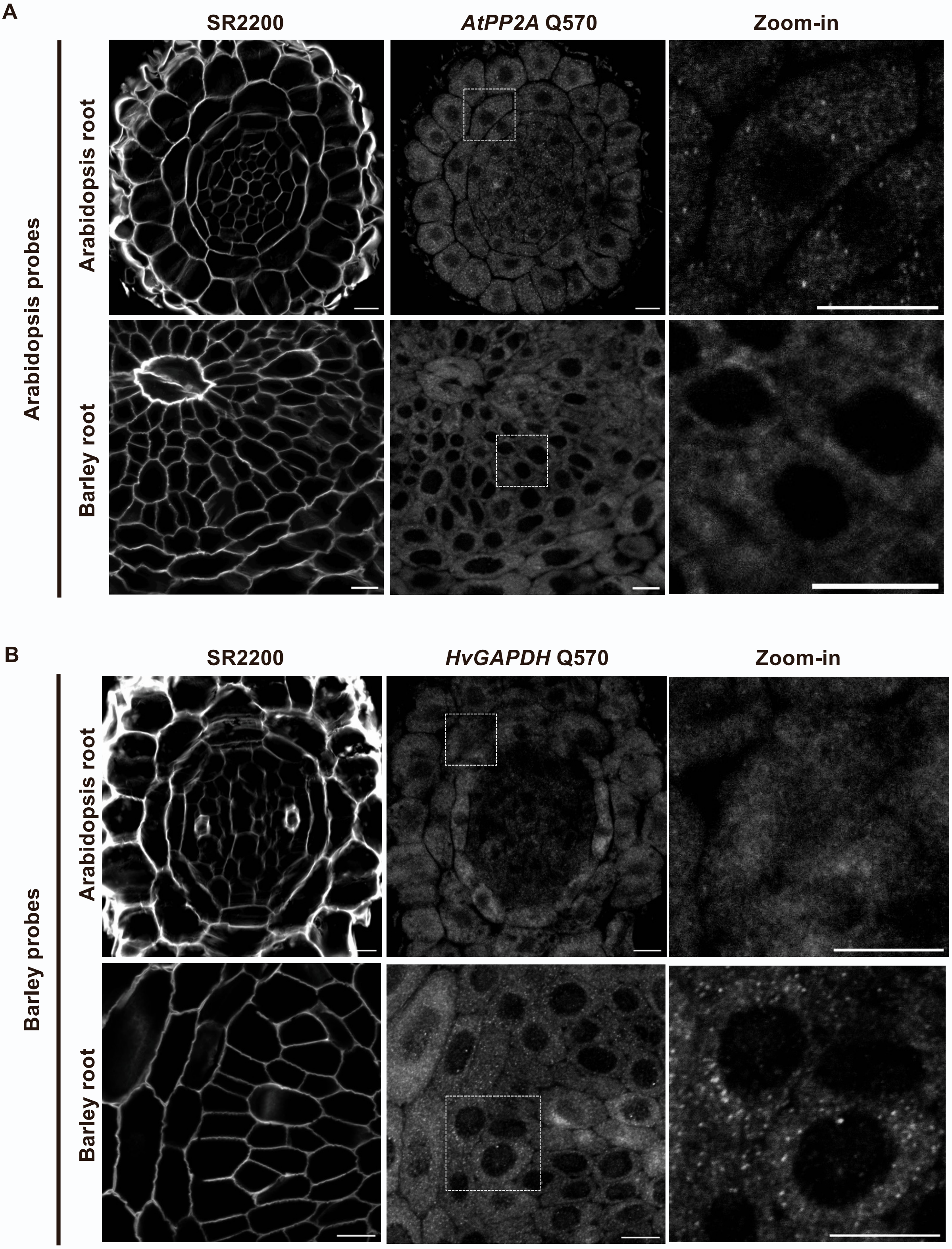

Figure S9. Cross-species validation of cryo-smFISH probe specificity.
(A) Representative single-plane confocal images of cryo-smFISH using probes designed to detect *PP2A* mRNA from *Arabidopsis.* Root cross-cryosections from Arabidopsis and barley were tested, showing signals exclusively in Arabidopsis samples. (B) Representative images of cryo-smFISH using probes designed to detect *GAPDH* mRNA from barley. Root cross-cryosections from *Arabidopsis* and barley were tested, showing signals exclusively in barley samples. Left panels: cell wall staining with SR2200 dye; Middle panels: smFISH channel; Right panels: zoomed-in images of smFISH channel. Scale bars, 10µm.

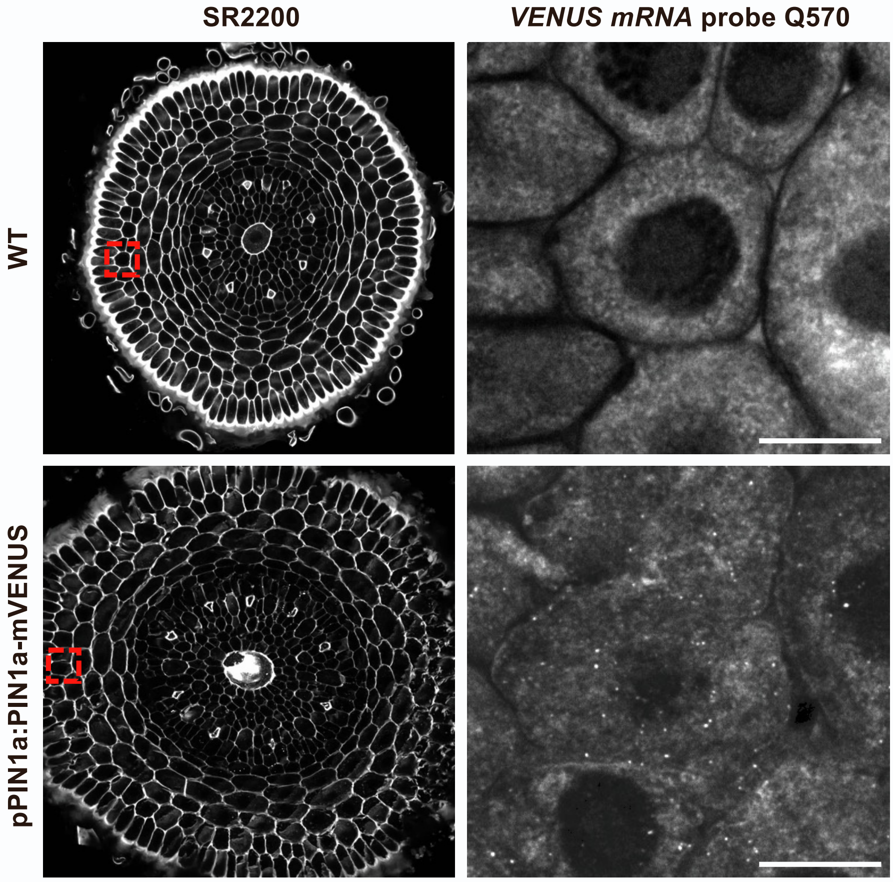

Figure S10. smFISH probe specificity control using *VENUS* mRNA probes
Representative single-plane confocal images of cryo-smFISH using Quasar570-probes designed to detect *VENUS* mRNA. Root cross-cryosections from barley were tested, showing signals exclusively in the transgenic line pPIN1a:PIN1a-mVENUS. Left panels: cell wall staining with SR2200 dye; Right panels: zoomed-in images of smFISH channel. Scale bars, 10µm.

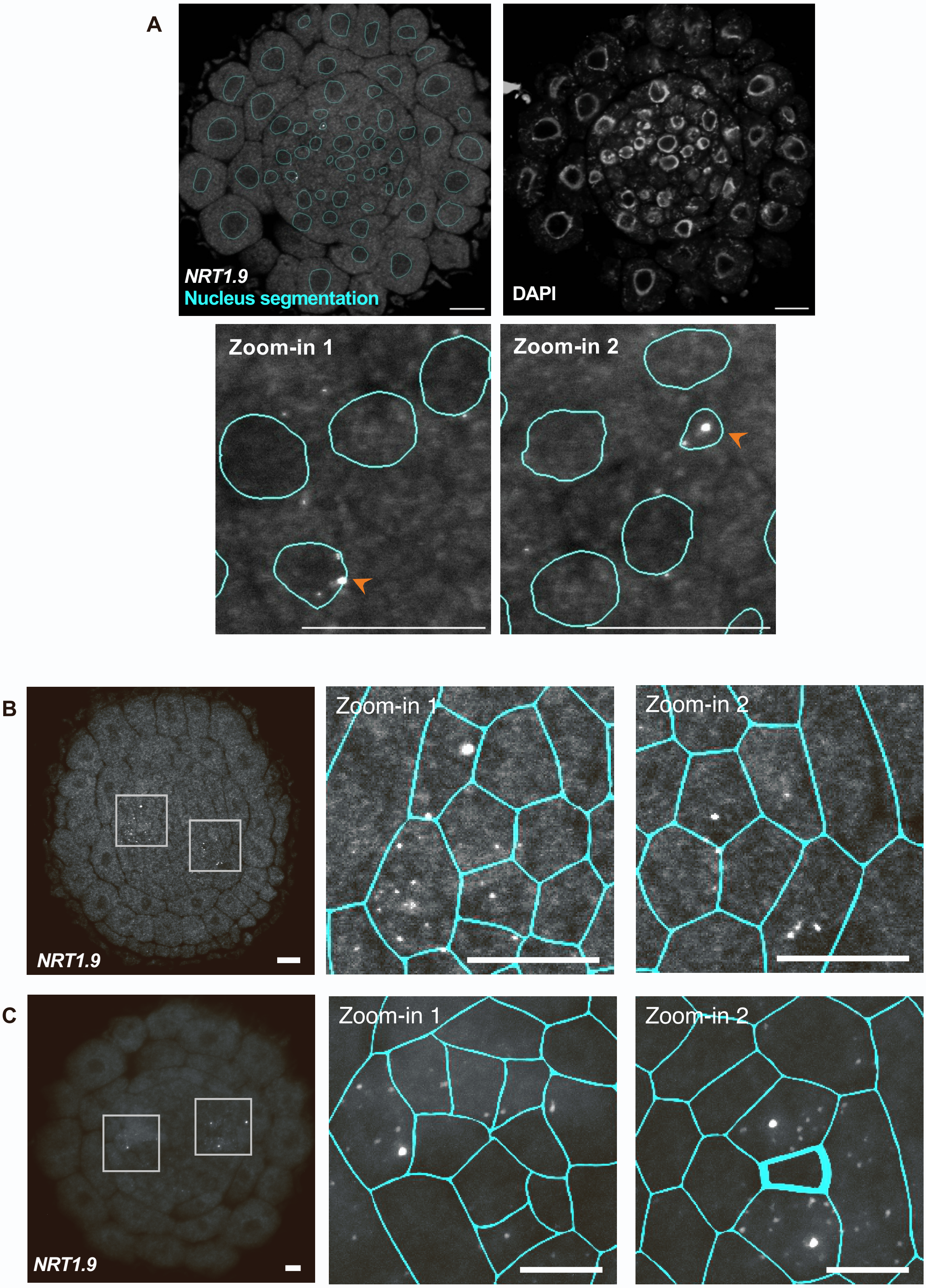

Figure S11. ***NRT1.9*** **expression pattern visualized by cryo-smFISH.**
(A-C) Representative images of cryo-smFISH using probes targeting *NRT1.9* mRNA in Arabidopsis root cross-cryosections. Images in (A) and (B) are single-plane confocal images, whereas the image in (C) is a maximum intensity projection from 17 Z-slices acquired with widefield microscopy. (A) Lower row: zoomed-in images showing *NRT1.9* transcripts (white dots). The orange arrows indicate *NRT1.9* RNA foci. Nucleus outline (cyan) obtained from segmentation using DAPI. (B-C) Middle and right panels: zoomed-in images showing *NRT1.9* transcripts (white dots). Cell outlines (cyan) obtained from segmentation using SR2200 dye. Scale bars, 10 µm.

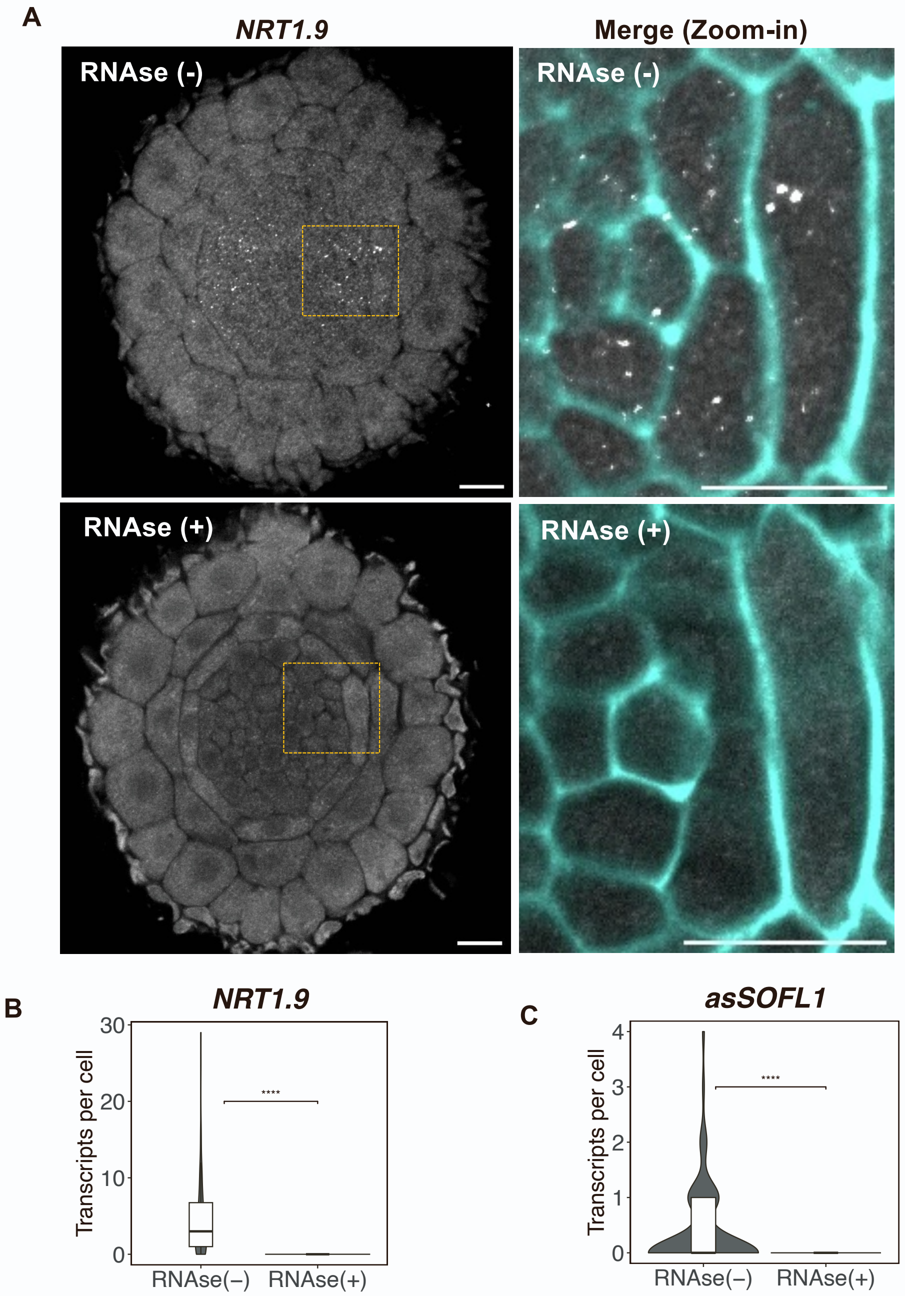

Figure S12. RNAse A controls for cryo-smFISH experiments on *NRT1.9* and *asSOFL1*.
(A) Representative single-plane confocal images of cryo-smFISH using probes targeting *NRT1.9* mRNA in *Arabidopsis* root cross-cryosections before (-) and after (+) RNAse treatment**.** Zoom-in images (right panels) of regions highlighted in yellow on the left panel. Discrete white dots correspond to individual RNA molecules. Cell walls are stained with SR2200 dye (cyan). Scale bars, 10µm. (B, C) Violin plots showing the distributions and quantification of the number of transcripts per cell within the imaged volume before RNAse (-) and after RNAse (+) treatment for *NRT1.9* and *asSOFL1*. The dashed lines in histograms indicate the median value. Violin plots: boxes inside indicate the interquartile range (IQR 25-75%), depicting the median values as a horizontal line. Whiskers represent the ±1.58xIQR value. A two-sided t-test was performed to compare both conditions. p-value less than 0.0001 was denoted by ****.

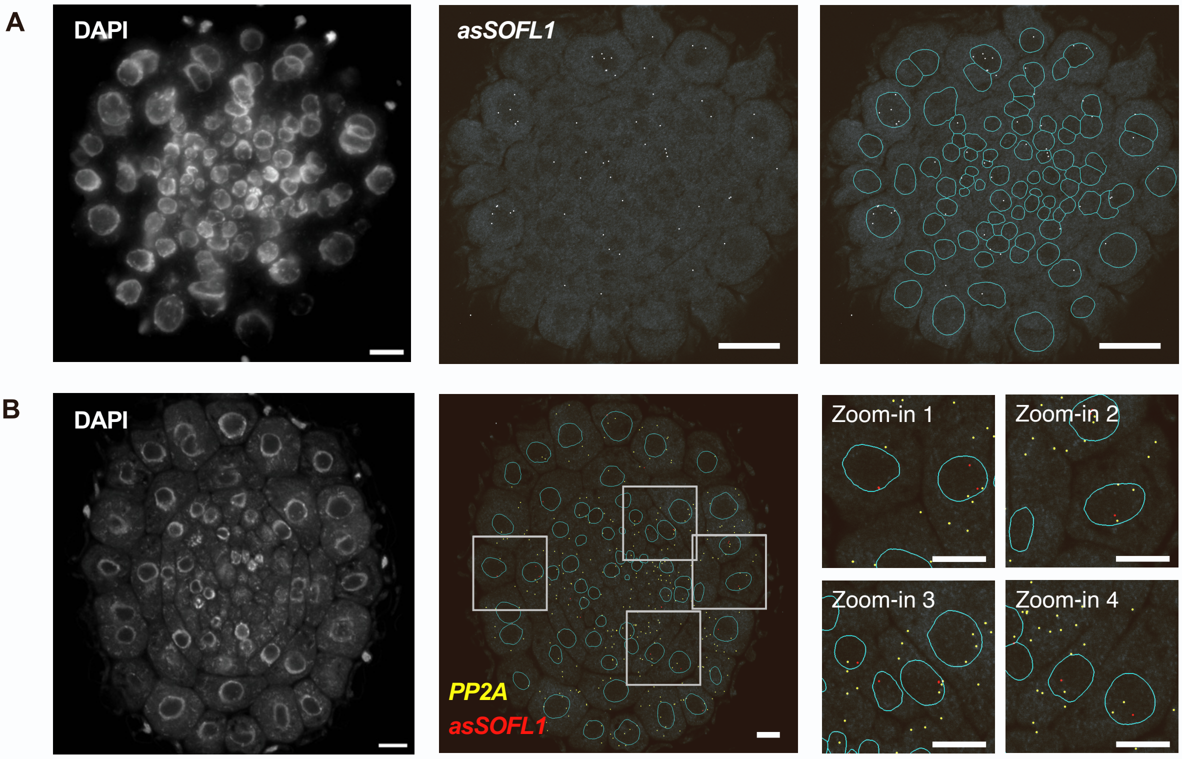

Figure S13. *asSOFL1* expression pattern visualized by cryo-smFISH.
(A) Representative Z project images of cryo-smFISH using probes targeting *asSOFL1* RNA in Arabidopsis root cross-cryosections. Right panel: image showing *asSOFL1* (white dots) transcripts within nucleus. Nucleus outlines obtained from segmentation using DAPI (blue). (B) Representative confocal image of cryo-smFISH using probes targeting *asSOFL1* (red dots) and *PP2A* (yellow dots) RNA in Arabidopsis root cross-cryosections. Right panel: zoom-in images. Scale bars, 10µm.

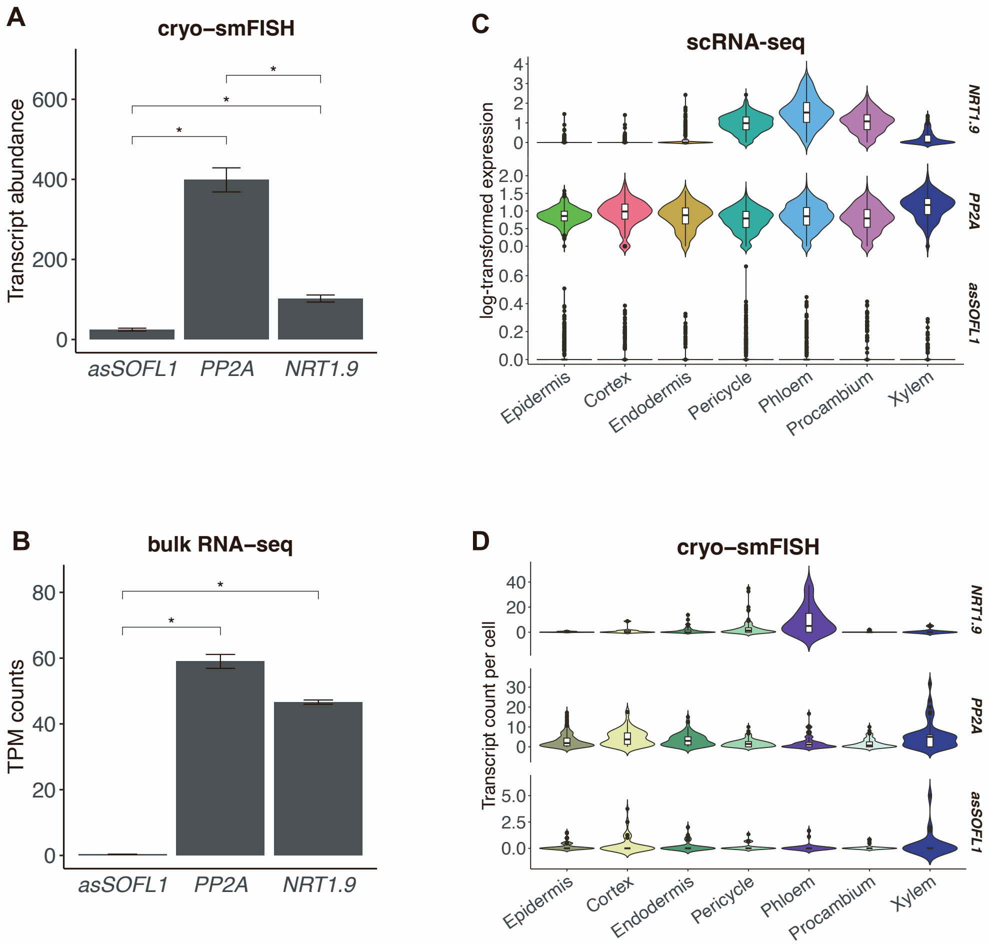

Figure S14: Comparison of *NRT1.9*, *PP2A* and *asSOFL1* expression between scRNA-seq, smFISH and bulk RNA-seq. (A, B) Bar plots showing the expression of *NRT1.9*, *PP2A*, and *asSOFL1* in the *Arabidopsis* root tip using cryo-smFISH (A) and bulk RNA-seq data (B). Error bars represent the mean ± SEM. Quantitative data for smFISH and bulk RNA-seq results originated from three replicates and two replicates, respectively. A two-sided t-test was performed to compare both conditions. A p-value greater than 0.05 indicates no statistical significance (not shown), while p-values less than 0.05, 0.001, and 0.0001 were denoted by*, **, and ***, respectively.(C, D) Violin plots showing the expression of *NRT1.9*, *PP2A,* and *asSOFL1* in individual cells detected by scRNA-seq (C) and cryo-smFISH (D). The number of transcripts is normalized by the number of cells within the cell type. The plotting area is scaled by width. Violin plots: boxes inside correspond to the interquartile range (IQR 25-75%), and horizontal lines indicate the median values. Whiskers show the ±1.58xIQR value. Statistical analyses were performed with one-way ANOVA followed by Tukey's honestly significant difference (HSD) tests.

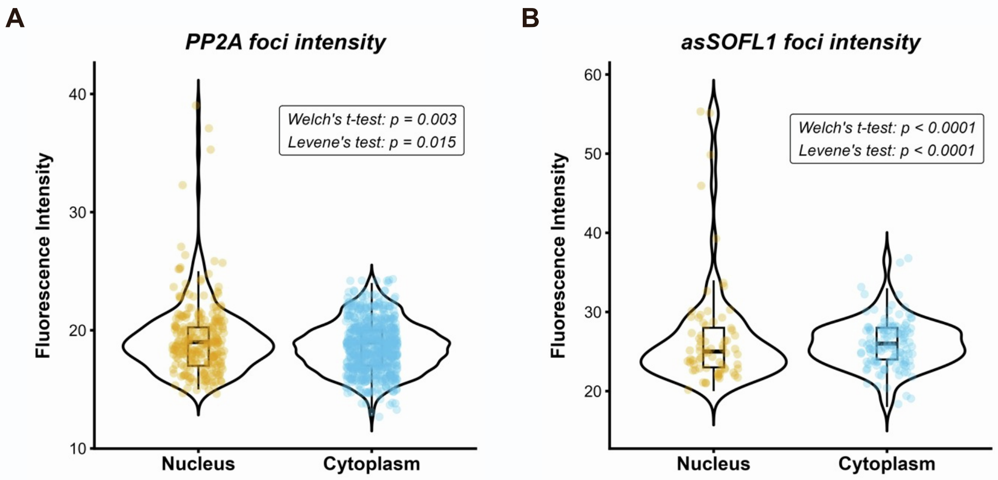

Figure S15. Subcellular smFISH signal intensity of *PP2A* and *asSOFL1* detected foci. Violin plots showing the raw fluorescence intensity of individual smFISH foci detected in the nucleus (orange) and cytoplasm (blue) for *asSOFL1* **(A)** and *PP2A* **(B)**. Data were pooled from 4 independent *Arabidopsis* root cross-sections, comprising a total of 953 detected transcripts for *PP2A* and 202 detected transcripts for *asSOFL1*. Individual data points are overlaid as jitter plots, where each dot indicates an individual detected spot. Boxes inside violin plots show the interquartile range (IQR 25-75%), indicating the median values as a horizontal line. Statistical analyses comparing the mean intensities between nuclear and cytoplasmic compartments were performed using Welch's t-test, and differences in variance were assessed using Levene's test. The respective p-values are indicated in the top right corner of each plot.

Table S1. Comparison of smFISH approaches in plants.

Feature	Cryo-smFISH	Whole-mount smFISH	Paraffin-section smFISH
**Main application**	Spatial detection and quantification of single RNA molecules in tissue sections	Spatial detection and quantification of single RNA molecules in intact tissues	Spatial detection of RNAs in embedded tissue sections
**Preservation of tissue architecture**	Moderate; can be challenging in fragile tissues	Good to very good	Good morphology after embedding and sectioning
**Suitability for fragile tissues**	Limited; requires optimization and careful handling	Often advantageous, as tissue remains intact	Can be better than cryosectioning for morphology, but embedding is laborious
**Preservation of 3D tissue context**	Limited to the section plane	Strong advantage; intact tissue context maintained	Limited to the section plane
**Suitability for thick specimens**	Advantageous, because sectioning reduces out-of-focus background	More challenging due to probe penetration and background in deeper layers	Suitable after sectioning, but embedding adds processing time
**Probe / reagent penetration**	Good in thin sections	More challenging, especially in dense tissues	Good in sections after embedding
**Compatibility with immunofluorescence**	Strong advantage; antibody penetration is generally easier in sections	More difficult due to limited antibody penetration	Possible, but antigen preservation may be affected by embedding/processing
**Autofluorescence issues**	Still challenging in some tissues; far-red dyes may help	Also challenging, sometimes exacerbated by tissue thickness	Depends on tissue and processing
**Imaging requirements**	Can be imaged with widefield or confocal microscopes	Often benefits from confocal imaging, especially in thicker tissues	Widefield or confocal depending on sample and signal
**Detection of short RNAs**	Limited for short targets because multiple probes are needed	Similar limitation	Similar limitation
**Technical difficulty**	Moderate; sectioning quality is critical	Moderate to high; penetration, clearing, and mounting can be demanding	High; embedding and sectioning are time-consuming

Table S2. Cryo-smFISH strenghts and limitations.

**Strengths**	- Detection and visualization of single molecules of RNAs with cellular and sub-cellular resolution in plant tissue sections;<br>- Easy to implement and relatively fast compared to other sectioning methods;<br>- Particularly advantageous for thick specimens, where sectioning reduces out-of-focus background and can improve signal-to-noise ratio compared with whole-mount preparations;<br>- Images can be acquired with widefield and confocal microscopes;<br>- More suitable than whole-mount preparations for combined smFISH and immunofluorescence, as antibody penetration is generally more efficient in cryosections.<br>- Can be used to validate single-cell RNA seq. No transgenic lines are needed;<br>- Can be implemented in non-model species.
**Limitations**	- Tissue preservation can be challenging in fragile tissues, such as Arabidopsis roots or leaves, and often requires optimization and practice; in such cases, whole-mount approaches may better preserve tissue integrity and overall morphology.<br>- Highly autofluorescent tissues, such as adult leaves, still present challenges for detection. The signal-to-noise ratio can be improved by selecting fluorophores with emission wavelengths that minimize tissue autofluorescence. In our study, we observed that leaf autofluorescence was generally lower in the far-red range, suggesting that Quasar670 may be a more suitable choice for these tissues. However, as plant tissues contain multiple autofluorescent compounds, the optimal fluorophore selection should be carefully adjusted based on the specific tissue and its autofluorescence profile.<br>- Cannot be used to study more than 4 genes at the same time due to fluorophore limitations (i.e., only a small number of colors can be used for microscopy);<br>- Cannot be used to study RNA that are shorted than ~600nt in length (probes are 18-22 mers DNA oligonucleotides and a minimum of 25-30 probes is required per target);<br>- The probes are particularly costly, which can limit their routine use.<br>- Cryosectioning provides only a section of the tissue, whereas whole-mount approaches may better retain three-dimensional tissue context.

Table S3. smFISH probe sequences used in this study and the corresponding dyes (Quasar570 or Quasar670). Probe Sequences (5’-3’).

Probe nr	*PP2A* (Quasar570)	*HvGAPDH* (Quasar570)	*NRT1.9* (Quasar670)	*asSOFL1* (Quasar670)	*VENUS* (Quasar570)
1	ccgagcgatctatcaatcag	ggttttataaagccaggagc	caaatgtctcatttcctacc	gaccagctgaattcagtct	cggtgaacagctcctcgc
2	gacatcctcaccaaaactca	agaggtggggagggtttaag	cagattcgatgaacttccaa	cgaatccagaggctttctt	cagctcgaccaggatggg
3	tcgggtataaaggctcatca	agtgtctggaagcggatatg	cagccgtgatgctcttcat	gcaacgaaagccaccaagt	ttgtggccgtttacgtcg
4	tagctcgtcgataagcacag	cgaaggagtggaactgacgc	ccgtagatgttgacgacttt	atcacaagcgacgtttcca	ctcgccggacacgctgaa
5	ccaagagcacgagcaatgat	cgatcttaatcttgcccatg	gatggtgccaaagttgcttg	aggtccacattaaacccac	ttgccgtaggtggcatcg
6	atcaactcttttcttgtcct	atccttccgaaaccgttgat	gagtcacagaggaaagcagc	gtttctcggaggcacaatc	gtggtgcagatcagcttc
7	catcgtcattgttctcacta	tgatgaaggggtcgttgacg	aagatagggtcttgtagcgg	tccttttgtctgattccat	agggtggtcacgagggtg
8	atagccaaaagcacctcatc	catgtacgtcatgtactcgg	agaaagcaagcgatcatggc	atccacagccacacacaaa	aagcactgcaggccgtag
9	atacagaataaaaccccca	cgtgaactgtgtcgtacttg	cgaaccctttgcatcaaaca	cgagtttcttcgtaccgat	catgtggtcggggtagcg
10	caagtttcctcaacagtgga	atgtcactgtgcttccaatg	gatgcaggggatgaatcaca	tgaagctactacattgccc	tgaagaagtcgtgctgct
11	tcatctgagcaccaattcta	agcgtcttgtcgtctttgag	tatttccttagcgcattgag	gttcactccatttctggat	acgtagccttcgggcatg
12	tagccagaggagtgaaatgc	cctgacgccaaagacagtaa	tggcccattgcacacgctt	tgcttgtttgcatcactca	agaagatggtgcgctcct
13	cattcaccagctgaaagtcg	gtggactccacaacgtaatc	gaaacatgatttgtccgatg	atcctctgctgatcacatg	tcttgtagttgccgtcgt
14	ggaaaatcccacatgctgat	ttgtccttgtcagtgaagac	accagcaaaaccattgctcc	accattcctgaattgcagg	tcgaacttcacctcggcg
15	atattgatcttagctccgtc	cagagatgaccaccttcttg	atggcctaatcccaccggct	gagtcttggactagccata	ctcgatgcggttcaccag
16	attggcatgtcatcttgaca	aaacataggggcatctttgc	gatcagcaccaaatggaag	agatgtgcacgttcggtag	tgaagtcgatgcccttca
17	aaattagttgctgcagctct	ttgtcctcattgacaccaac	ttcttttgtcttcggatcga	ttcctgctagttacaagct	caggatgttgccgtcctc
18	gctgattcaattgtagcagc	atgttaacatccgaggtgta	aaactctcaatccctcgttt	aatagcatgtgcacgttcc	ttgtactccagcttgtgc
19	ccgaatcttgatcatcttgc	gtgcagctagcatttgagac	gtgaaggtgaagaaatacc	cccaaccttcacgaagatt	agacgttgtggctgttgt
20	caaccctcaacagccaataa	taatgaccttagctagggga	cgatgagggttaacgacacc	tgttattctcaggtcgctt	tgcttgtcggcggtgata
21	ctccaacaatttcccaagag	tcagaccctcaataatacca	cagctcacgtttgactgaac	aagaggaagaccctcacgt	gaagttggccttgatgcc
22	caaccatataacgcacacgc	tgatggcatgaacagtggtc	agctgggatggctaaacca	acatagctggcaactttcc	cgatgttgtggcggatct
23	agtagacgagcatatgcagg	catcaacggtcttctgtgtg	tgcaaccaagtagcattag	gcatatcctgataagcgca	gtagtggtcggcgagctg
24	gaacttctgcctcattatca	gctggggatgatgttaaagc	gagcttagaaccagcgaaga	tcttcagagacttccttct	cgatgggggtgttctgct
25	cacagggaagaatgtgctgg	cattcagctcaggaagaacc	ccactagctttaaccttgac	agttatctgcacggttctc	ttgtcgggcagcagcacg
26	tgacgtgctgagaagagtct	ccggaaagacataccggtaa	acacgagttatgctgtgaa	ttttgtagaagctgtccca	ggactggtagctcaggta
27	cccattataactgatgccaa	aactgacacatccacagtgg	cgcctcttcttgatcgcgac	attgttttcttttgccgga	ttggggtctttgctcagg
28	tggttcacttggtcaagttt	cggttctaacagtgagatca	aagctcgttaggcccaacgg	agccatgtgtaggtagttg	catgtgatcgcgcttctc
29	tctacaatggctggcagtaa	catcatatgatgcagccttc	tggcccaatttcgagttct	caatcgtgttgtttgcctt	ggtcacgaactccagcag
30	cgattatagccagacgtact	ccttgatagccttcttgatg	ttagtacctgaactgctctg	gcaatcagcttccaaagca	tccatgccgagagtgatc
31	gactggccaacaagggaata	catgataccсttgagctttc	actaagagaagtagtgagg	gtgatgattccatggcttc	
32	catcaaagaagcctacacct	accaaatcttcctcaacgta	ttggattgccgatttgtcaa	agacgccttccatggaaat	
33	ttgcatgcaaagagcaccaa	tgtcaccaacgaagtcggtg	catctttgttcagcttgtcg	acagcagttgtgagtcagg	
34	acggattgagtgaaccttgt	agccttagcatcaaagatgc	gtttccatgcatccaccggc	gaggctctgaagaggagga	
35	cttcagattgtttgcagcag	cgaaatggtcgttcagagca	ctccacttgttgcatactg	ccatggagtctccgagaaa	
36	ggaccaaactcttcagcaag	tgtcataccacgagacaagc	acccggatcacgcatttaac	cttggttttgttttcctct	
37	ggaactatatgctgcattgc	cgacaacacggttgctgtaa	taaggcggccgagagccaaa	tatgaggcttcctataggc	
38	gtgggttgttaatcatctct	ggaaagcagaacgctctact	gttgaatgtaagctaggtag	ggttgccatgtggtgaaac	
39	tgcacgaagaatcgtcatcc	catagacaaaggggcacctc	ctgaaagattgtgtaggttg	tttgtctggtagtgcttca	
40	ttactggagcgagaagcgat	gacaccatccacatttattc	aggcgtctatcggattgaag	gaagggaggcactaacctt	
41	gaacatgtgatctcggatcc	aacatgaaccaggcgtctag	ggatctggaaacttcctgga	tgtttgttcatgtgggtct	
42	ctctgtctttagatgcagtt	cagatggtaactcatgtcca	aagacggtataagaaccggc	ggcctccactgaaagaaca	
43	catcattttggccacgttaa	acttgactagcaactcggtg	tgaatatcgtcattccaagc	tgatttgcaagctgtcacc	
44	cgtatcatgttctccacaac	cacttactcaggcaaacaga	tacaaggacgcggtcataga	caaattctacacgcccact	
45	atcaacatctgggtcttcac	caaacacttactcaggctgt	gcctgtatactttctaagga	gagtccaactccattcttt	
46	ttggagagcttgatttgcga	ggatcgcagcatcaaacatc	tctctgcagttgtgtgatac	gtgacattccacagtcaca	
47	acacaattcgttgctgtctt	gacgggagtagtactcaaca	atgcatagaaacagtcctgc	cccgatgatgctttcgaat	
48	cgcccaacgaacaaatcaca	cgctccgtcctataatataa	tgcagacaccatcatactt	caagttccatccttagtct	
